# Transcriptomic Changes in Mouse Bone Marrow-Derived Macrophages Exposed to Neuropeptide FF

**DOI:** 10.3390/genes12050705

**Published:** 2021-05-09

**Authors:** Yulong Sun, Yuanyuan Kuang, Zhuo Zuo

**Affiliations:** 1 School of Life Sciences, Northwestern Polytechnical University, Xi’an 710072, China; kuangyuanyuan@mail.nwpu.edu.cn (Y.K.); zuozhuo@mail.nwpu.edu.cn (Z.Z.); 2 Key Laboratory for Space Biosciences & Biotechnology, Institute of Special Environmental Biophysics, School of Life Sciences, Northwestern Polytechnical University, Xi’an 710072, China

**Keywords:** neuropeptide, bone marrow-derived macrophage, RNA sequencing, bioinformatics, transcriptomic profiles

## Abstract

Neuropeptide FF (NPFF) is a neuropeptide that regulates various biological activities. Currently, the regulation of NPFF on the immune system is an emerging field. However, the influence of NPFF on the transcriptome of primary macrophages has not been fully elucidated. In this study, the effect of NPFF on the transcriptome of mouse bone marrow-derived macrophages (BMDMs) was explored by RNA sequencing, bioinformatics, and molecular simulation. BMDMs were treated with 1 nM NPFF for 18 h, followed by RNA sequencing. Differentially expressed genes (DEGs) were obtained, followed by GO, KEGG, and PPI analysis. A total of eight qPCR-validated DEGs were selected as hub genes. Subsequently, the three-dimensional (3-D) structures of the eight hub proteins were constructed by Modeller and Rosetta. Next, the molecular dynamics (MD)-optimized 3-D structure of hub protein was acquired with Gromacs. Finally, the binding modes between NPFF and hub proteins were studied by Rosetta. A total of 2655 DEGs were obtained (up-regulated 1442 vs. down-regulated 1213), and enrichment analysis showed that NPFF extensively regulates multiple functional pathways mediated by BMDMs. Moreover, the 3-D structure of the hub protein was obtained after MD-optimization. Finally, the docking modes of NPFF-hub proteins were predicted. Besides, NPFFR2 was expressed on the cell membrane of BMDMs, and NPFF 1 nM significantly activated NPFFR2 protein expression. In summary, instead of significantly inhibiting the expression of the immune-related gene transcriptome of RAW 264.7 cells, NPFF simultaneously up-regulated and down-regulated the gene expression profile of a large number of BMDMs, hinting that NPFF may profoundly affect a variety of cellular processes dominated by BMDMs. Our work provides transcriptomics clues for exploring the influence of NPFF on the physiological functions of BMDMs.

## 1. Introduction

In the process of exploring the regulation mechanism of the immune system, the bone marrow is an excellent research object as it is the natural mother of almost all immune cells, which can affect almost all physiological systems [1,2]. Among the many immune cell types derived from bone marrow, bone marrow-derived macrophages (BMDMs) have received continuous attention. BMDMs play a vital role in various immune events, including macrophage polarization, pathogen invasion, and natural immunity [3,4]. Although the regulatory mechanism for BMDMs has not yet been fully elucidated, there is increasing evidence that the differentiation and function of BMDMs are regulated by diverse molecules, including hormones, inflammatory factors, and neuropeptides [2,4,5].

Neuropeptide FF (FLFQPQRFamide, NPFF) is a neuropeptide originally isolated from the bovine brain [6]. NPFF belongs to a family of neuropeptides, which includes two ligands (NPFF and NPAF) and two receptors (NPFF receptor 1 and NPFF receptor 2) [7,8]. According to the structural characteristics of NPFF (share a similar C-terminal sequence), NPFF is considered to belong to a family called RF-amide, which includes gonadotropin-inhibitory hormone (GnIH), pyroglutamylated RFamide (QRFP), kisspeptin, and prolactin-releasing peptide (PrRP) [8]. RF9 is considered to be a pharmacological antagonist of NPFFR2 [9].

Although NPFF was originally discovered as an opioid regulatory peptide, the biological activity of NPFF in other physiological fields is currently being investigated, including water balance [10], corneal nerve injury repair [11], analgesia [12,13,14], food intake [15,16,17,18,19], placental development [20], cardiovascular modulation [21], adipose metabolism [22], anxiogenic activity [23,24,25], seizure attenuation [26], and body temperature balance [27]. Recently, the regulation of immune activity by the NPFF system has become an emerging field.

The regulatory role of NPFF in immune and inflammatory response has attracted recent attention. NPFFR2 and NPFF are activated at the spinal cord of a rat inflammatory hyperalgesia model [28,29]. Moreover, NPFF is expressed at the inflammation site of a rat model of carrageenan-induced inflammation [30]. In the same line, NPFF down-regulates the nitric oxide (NO) level in RAW 264.7 macrophages and mouse peritoneal macrophages and attenuates the inflammatory reaction of a mouse model of carrageenan-induced inflammation [31,32]. Furthermore, NPFF enhances M2 macrophage activation of adipose tissue macrophage [22]. Collectively, the above evidence suggests that NPFF has active activity in the fields of immunity and inflammation. However, the mechanism by which NPFF regulates the immune system has not been fully revealed.

**Figure 1:**
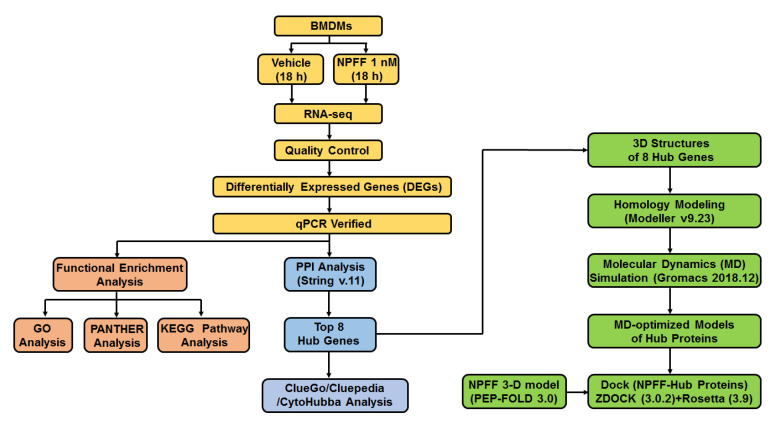
Schematic diagram of the present study.

Here, we present our efforts to explore the impact of NPFF on the transcriptomics of BMDMs by using methods including RNA-seq and bioinformatics, which may provide clues to investigate the regulation of NPFF on macrophages. RNA sequencing (RNA-seq) is a widely used technology that can provide valuable clues in revealing the mechanism of NPFF regulating immune cells. Therefore, identifying NPFF-triggered gene expression profile of macrophages will be helpful in investigating clues for NPFF to regulate macrophages. The aim of the present study is to: (1) acquire NPFF-sensitive differentially expressed genes (DEGs) in BMDMs by using RNA-seq, and investigate the pathways provoked by DEGs; (2) identify critical hub genes of DEGs, and construct the three-dimensional protein structure of hub genes; (3) investigate the structural changes of hub proteins on a microscopic time scale (at least 300 ns); and (4) predict the docking sites of NPFF and hub proteins using the peptide-protein docking module of the Rosetta program. By studying the effect of NPFF on the gene expression of BMDMs at the transcriptome level, our data provides clues for exploring the gene expression network of NPFF on macrophages, which will be helpful to investigate the immune-regulating function of NPFF (Figure 1). 

## 2. Materials and Methods

### 2.1. Ethical Statement

The study was conducted according to the guidelines of the Declaration of Helsinki and approved by the Ethics Committee of Northwestern Polytechnical University (protocol code 201900048, 2 January 2020). 

### 2.2. Mice

Male C57BL/6 mice (18–22 g) were housed in standard plastic cages with a temperature of 20–22 °C and humidity of 65–74%. Settled light cycle and access to water and food ad libitum were provided. Mice received gentle care, and all actions were taken to minimize mice suffering during the whole experiment process. The mice were handled under the 3Rs principle, and were sacrificed by CO_2_ inhalation.

### 2.3. Materials

Dulbecco’s modified Eagle’s medium (DMEM), TRIzol, Fetal bovine serum (FBS), and β-mercaptoethanol were acquired from Gibco™ and Invitrogen™ (Thermo Fisher Scientific, Inc., Waltham, MA, USA). Trypsin-EDTA solution (0.05% Trypsin—EDTA) and streptomycin (10,000 μg/mL)/penicillin (10,000 units/mL) antibiotics were purchased from Merck-Millipore.

Cell culture dishes were from Corning, Inc. (Corning, NY, USA). QiaQuick PCR extraction kit was acquired from Qiagen (Venlo, The Netherlands). SYBR^®^ Premix Ex Taq TM II kit and PrimeScript ^TM^ 1st Strand cDNA Synthesis Kit were from TaKaRa (Dalian, China). The red blood cell lysing buffer was from Beyotime (Beyotime, Shanghai, China). L-929 cells were provided by the Stem Cell Bank of the Chinese Academy of Sciences (Shanghai, China). A cell counting Kit-8 was purchased from Beyotime (Beyotime, Shanghai, China). All other reagents were purchased from commercial sources.

The Rabbit anti-NPFFR2 polyclonal antibody, which was used in previous reports [22,33], was provided by Biorbyt (No.orb31952, San Francisco, CA, USA). Anti-Rabbit IgG (Alexa Fluor 488 Conjugate) was provided from Cell Signaling Technology (Beverly, MA, USA). Horseradish Peroxidase conjugated-goat anti-rabbit IgG and goat anti-mouse IgG (H + L) were from Thermo Fisher Scientific (Beverly, MA, USA). Mouse anti-Actin monoclonal antibody was purchased from Sigma (St. Louis, MO, USA). BCA Protein Assay Kit was acquired from Thermo Scientific Pierce (Bedford, MA, USA). ECL detection kit, PVDF membrane, and protease inhibitor cocktail III (EDTA-free) were purchased from Millipore Corporation (Bedford, MA, USA). The PE rat-anti mouse F4/80 antibody and FITC rat anti-mouse CD11b antibody were from BD Pharmingen (San Diego, CA, USA).

NPFF was synthesized by GL Biochem Ltd. (Shanghai, China) using the solid-phase peptide synthesis method. The mass of NPFF was confirmed using a mass spectrometer (LCMS-2010EV, Shimadzu, Japan). NPFF was purified by HPLC, and peptides demonstrated > 98% purity.

### 2.4. Isolation of BMDMs

Femur and tibia were collected from six male mice and BMDMs were collected from the single-cell suspensions by flushing tibias and femurs with ice-cold PBS. Cell suspensions were centrifuged (1200 rpm/min, at 4 °C for 7 min), the supernatant was removed, and the pellet was resuspended with 3 mL of red blood cell lysing buffer (Beyotime, China) for 5 min. Next, the cell suspensions were centrifuged (800 rpm/min, at 4 °C for 5 min), the supernatant was removed, and the pellet was resuspended with 10 mL of PBS. Subsequently, the cell suspensions were filtered through a sterile 100 mesh filter to obtain a single-cell suspension.

The single-cell suspensions were cultured in DMEM (10% FBS, streptomycin (100 μg/mL)/penicillin (100 units/mL)) at 37 °C with 5% CO_2_ in a fully humidified incubator. After one night, the non-adherent cell supernatant was collected, centrifuged (800 rpm/min, at 4 °C for 5 min), and the pellet was resuspended in the complete cell culture medium. Subsequently, the cells were cultured in 100-mm culture dishes supplemented with complete DMEM with 20% L-929 conditioned media for 8 d (the medium was refreshed every 3 d). After 8 d, the cells were differentiated into BMDMs, where over 90% of the cells were double-positive for CD11b and F4/80.

### 2.5. Cell Sample Preparation and Microscope Detection

BMDMs were treated with NPFF (1 nM) for 18 h and subjected to RNA sequencing examination. Cells were rinsed with PBS three times and lysed with TRIzol (1 mL). Then, cell lysates were immediately stored in liquid nitrogen. Finally, the RNA-seq detection was conducted by the Novogene Co Ltd. (Beijing, China).

Cell images were taken by a microscope (Nikon 80i, Japan). Besides, the detailed structure of BMDMs was examined by a transmission electron microscope HT7700 (Hitachi High-Technologies, Japan).

### 2.6. Cell Viability Assay

Cell viability was examined using a cell counting Kit-8 (Beyotime, Shanghai, China). BMDMs were seeded in a 96-well plates at a density of 50,000/well and incubated with or without NPFF (1 nM) for 18 h. Cell viability was examined by quantitative colorimetric test with CCK-8. One hundred μL of CCK-8 solution was added to each well, and then incubated in the cell incubator for 3 h. Next, the absorbance (450 nm) was determined using a SYNERGY-HT multiwell plate reader (Synergy HT, Bio-Tek instruments, Winooski, VT, USA). 

### 2.7. Flow Cytometry Experiment

BMDMs were washed twice with pre-cooled buffer (BSA-PBS-1%) and gently washed once with trypsin (0.25%). The cells were resuspended in fresh medium to a uniform cell suspension, followed by centrifugation at 1000/rpm for 5 min. Subsequently, the pelleted cells obtained by centrifugation were resuspended in a buffer solution (BSA-PBS-1%) into a uniform cell suspension. After standing for 5 min, the cells were centrifuged at 1000/rpm for 5 min. Next, the pellet obtained by centrifugation was resuspended in the buffer and counted (1 × 10^6^/100 μL). The antibody was added to the cell suspension and incubated in the dark for 25 min. During the incubation, the cells were mixed every 2 min so that the antibody and cells could be fully combined. Then, the cell suspension was subjected to centrifugation at 1000 rpm/min for 5 min, and then centrifuged supernatant was removed and discarded. Buffer (400 μL) was added to the tube to resuspend the cell pellet, and the cell suspension was filtered using a 300-mesh sterile filter to ensure that only single cells were subjected to flow cytometry detection. The purity of macrophages was tested by flow cytometry (BD Calibur, Biosciences, CA, USA) and analyzed by Cellquest (BD) and Modfit software.

### 2.8. RNA-Seq Sample Collection and Preparation

#### 2.8.1. RNA Qualification and Quantification

BMDMs RNA degradation and contamination were tested on 1% agarose gels. RNA purity was detected with a NanoPhotometer^®^ spectrophotometer (IMPLEN, CA, USA). RNA integrity was assessed with the RNA Nano 6000 Assay Kit of the Bioanalyzer 2100 system (Agilent Technologies, CA, USA). RNA concentration was investigated with the Qubit^®^ RNA Assay Kit of Qubit^®^2.0 Flurometer (Life Technologies, CA, USA).

#### 2.8.2. Library Preparation for RNA Sequencing

A total of one µg RNA (per sample) was isolated as input material for the RNA sample detection. Sequencing libraries were prepared with a NEBNext^®^ UltraTM RNA Library Prep Kit for Illumina^®^ (Lincoln, NEB, USA) following the manufacturer’s protocols.

Briefly, mRNA was extracted from total RNA by using the poly-T oligo-attached magnetic beads. Fragmentation was conducted with the NEBNext First Strand Synthesis Reaction Buffer (5×). First-strand cDNA was synthesized with random hexamer primer and the M-MuLV Reverse Transcriptase (RNase H-). Second strand cDNA synthesis was then performed with the DNA Polymerase I and RNase H. Remaining overhangs were transformed into blunt ends with exonuclease/polymerase activities. Subsequently, the 3′ ends of DNA fragments were adenylated, and NEBNext Adaptor with hairpin loop structure was ligated for next hybridization. Next, the library fragments were purified with an AMPure XP system (Beckman Coulter, Beverly, USA) to obtain cDNA fragments of preferentially 250~300 bp in length. Next, USER Enzyme (3 µL) (NEB, USA) was used with adaptor-ligated, size-selected cDNA for 15 min (37 °C), followed by 5 min (95 °C) before a PCR test. Then, a PCR was performed with the Index (X) Primers, Phusion High-Fidelity DNA polymerase, and Universal PCR primers. Finally, PCR products were isolated with an AMPure XP system, and library quality was detected using an Agilent Bioanalyzer 2100 system.

#### 2.8.3. Sequencing and Clustering

The clustering of the index-coded samples was performed with the TruSeq PE Cluster Kit v3-cBot-HS (Illumia) and the cBot Cluster Generation System. After cluster generation was conducted, the library samples were sequenced using the Illumina Hiseq platform, which finally acquired 125 bp/150 bp paired-end reads.

### 2.9. RNA-Seq Data Interpretation

#### 2.9.1. Quality Control

Raw reads were processed using in-house Perl scripts. After low-quality reads were removed, clean reads were obtained. Then, Q20, Q30, and GC values of the clean reads were calculated. The clean data with high quality were used for the downstream analyses.

Musculus genome and gene model annotation files were acquired from the genome website directly. Index of the reference genome was built with the Hisat2 v2.0.5, and paired-end clean reads were aligned to the reference genome with the Hisat2 v2.0.5.

#### 2.9.2. Reads Mapping to the Musculus Reference Genome

Musculus genome and gene model annotation files were acquired from the genome website directly. Index of the reference genome was built with the Hisat2 v2.0.5, and paired-end clean reads were aligned to the reference genome with the Hisat2 v2.0.5.

#### 2.9.3. Quantification of Gene Expression

The FeatureCounts v1.5.0-p3 was employed to calculate the reads numbers of each gene. Then FPKM of each gene was counted, and the read count was mapped to each gene.

#### 2.9.4. Differential Expression Interpretation

Differential expression analysis was performed with the DESeq2 R package (1.16.1). The *p*-values were adjusted with the Benjamini and Hochberg’s method to assess the false discovery rate. Genes with an adjusted *p*-value < 0.05 were selected as differentially expressed. The heat map of DEGs was generated by the toolkit TBtools [34].

#### 2.9.5. GO and KEGG Enrichment Analysis

To explore the ontology (GO) enrichment of differentially expressed genes (DEGs), the ClusterProfiler R package [35] was used in this step. GO terms with adjusted *p* < 0.05 were selected as significantly enriched.

To investigate the pathways associated with DEGs, the enrichment analysis was conducted with Metascape online tool (http://metascape.org/, accessed on 11 November 2020) [36], and the remarkedly biological processes were acquired with the DEG lists. 

To further interpret the functions of DEGs, DEGs were analyzed with PANTHER (http://www.pantherdb.org/, accessed on 11 November 2020) [37]. Three main GO categories include biological process (BP), molecular function (MF), and cellular component (CC) were exhibited, respectively.

In addition, KEGG (http://www.genome.jp/kegg/, accessed on 11 November 2020) was employed to investigate the functional enrichment of DEGs. In order to show the KEGG pathway maps clearly, KEGGParser (a Cytoscape plug-in) was used to interpret biological networks.

#### 2.9.6. Protein–Protein Interaction (PPI) Network Analysis

The STRING database (http://string-db.org, accessed on 15 November 2020) aims to provide a comprehensive assessment and integration of protein–protein interactions (PPI), including indirect (functional) as well as direct (physical) associations [38]. STRING (version 11.0) covers 24,584,628 proteins from 5090 organisms (4445 bacteria, 477 eukaryotes, and 168 archaea) and 3,123,056,667 total interactions.

To investigate the interactive relationship among DEGs, all DEGs were subjected to STRING analysis. Only experimentally proved interactions with a combined score above 0.4 were selected as significant. Then, the PPI network was constructed with the Cytoscape software (Ver3.8.0). The Cytoscape plug-in Molecular Complex Detection (MCODE) was used to detect the modules of PPI network. Moreover, function enrichment analysis for DEGs of the modules was performed, and *p* < 0.05 was considered as statistically significant.

To further interpret the pathways DEGs, pathway enrichment analysis was performed by Cytoscape software and the clueGO (http://apps.cytoscape.org/apps/cluego, accessed on 11 November 2020) [39]+ Cluepedia (http://apps.cytoscape.org/apps/cluepedia, accessed on 15 November 2020) [40] plug-in. In the present study, KEGG pathway enrichment detection was performed with ClueGO and CluePedia tool kits, and *p* < 0.05 and kappa coefficient 0.4 were considered as threshold values.

To identify the top-ranked hub genes in the PPI network, a Cytoscape plug-in CytoHubba was used in the following study. A total of eight hub genes were acquired based on the scores of several methods including Radiality, MCC, MNC, DMNC, Betweenness, Degree, BottleNeck, EPC, Closeness, EcCentricity, Clustering Coefficient, and Stress [41,42].

### 2.10. Gene Expression Analysis

This method has been previously described [43]. Total RNA was isolated from BMDMs with the TRIzol following the manufacturer’s instruction. cDNA was transcribed from 1 μg of RNA by using a PrimeScript ^TM^ 1st Strand cDNA Synthesis Kit. Gene expression level was investigated with the SYBR^®^ Premix Ex Taq TM II system and the MX3000P Real-Time PCR System (Stratagene). Real-time PCR procedures were set as follows: 94 °C for 30 s, 95 °C for 5 s, 56 °C. Data were normalized to GAPDH gene expression with the comparative 2^−ΔΔ^CT approach. Primers of genes were listed in Table 1. Gene expression data were tested in duplicates three times.

### 2.11. Western Blot

The method has been previously described [31]. Briefly, cells were gently washed with phosphate-buffered saline three times and lysed with lysis solution (5 mM EGTA, 150 mM NaCl, 50 mM Tris/HCl, PH 7.4, 1% Nonidet P-40, 0.1% SDS, 0.5% sodium deoxycholate, 1 unit protease inhibitor cocktail III (EDTA-free)). Cell lysates were then centrifuged at 12,000× *g* for 12 min at 4 °C, and the protein concentration of the supernatants was determined using the BCA Protein Assay Kit. Samples for immunoblot (18–25 µg of protein/lane) were analyzed by 10% SDS-polyacrylamide gel electrophoresis with the Bio-Rad mini-gel system. Then, the proteins were blotted onto PVDF membranes with the Bio-Rad wet blotter system. After electro-transfer, the membranes were treated with 5% non-fat milk in the Tris-buffered saline containing 0.05% Tween-20 (TBST) for 1 h. Subsequently, the membranes were washed three times with TBST, and were incubated overnight at 4 °C with appropriate antibodies (anti-NPFFR2 was 1:1000, anti-Actin was 1:5000, and anti-second antibody was 1:10,000). After three rinses with TBST, membranes were treated with horseradish peroxidase-conjugated secondary antibodies for two h at room temperature. Finally, membranes were analyzed using an ECL detection kit. The analysis of the band intensity was conducted with ChemDocTM XRS (Bio-Rad, Hercules, CA, USA) and Image J [44].

### 2.12. Immunofluorescence Stain Assay

Immunofluorescence assay was performed as previously described [32]. Briefly, cells were maintained on the glass slides and then fixed with 4% paraformaldehyde for 15 min. After being rinsed with PBS three times, cells were exposed to 0.1% Triton X-100 for 12 min and followed by 5% normal rabbit serum for three h. Subsequently, cells were treated with rabbit anti-NPFFR2 polyclonal antibody (1:250) overnight at 4 °C, followed by 1.5 h of incubation with anti-rabbit IgG (Alexa Fluor 488 Conjugate). Then, cells were incubated with DAPI (5 min) to stain the nucleus. All pictures were obtained with a confocal microscope (Leica TCS SP5, Leica Microsystems, Wetzlar, Germany).

### 2.13. Homology Modeling of Hub Proteins

See Supplementary File 1.

### 2.14. Molecular Dynamics (MD) Simulation

See Supplementary File 1.

### 2.15. Dock

Preparation: The 3-D structure of NPFF was predicted by PEP-FOLD3 [45], and the 3-D structure of hub proteins were acquired from the MD-optimized protein structures.

Docking process:

(1) The primary docking complex (consisting of NPFF (ligand) and hub protein (receptor)) was generated by ZDOCK (3.02) [46,47,48].

(2) The primary docking complexes were submitted to Rosetta (3.9) (Flexible peptide docking module) program for further docking. A. Pre-pack mode: one model was produced. B. Low-resolution ab-initio mode: 100 models were generated, and one model with the best docking score among the 100 models was selected for subsequent research. C. Refinement mode: 100 docking models were obtained, and a docking model with the best total score was finally selected.

### 2.16. Statistical Analysis

Data were shown as means ± standard error of the mean (S.E.M). Data were analyzed using the t-test method or the one-way ANOVA followed by the Tukey post hoc tests. The statistical interpretation was conducted with the GraphPad Prism software version 8.0 (San Diego, CA, USA). *p* < 0.05 were considered as statistically significant.

## 3. Results

### 3.1. The Effect of NPFF on the Morphology and Viability of BMDMs

**Figure 2:**
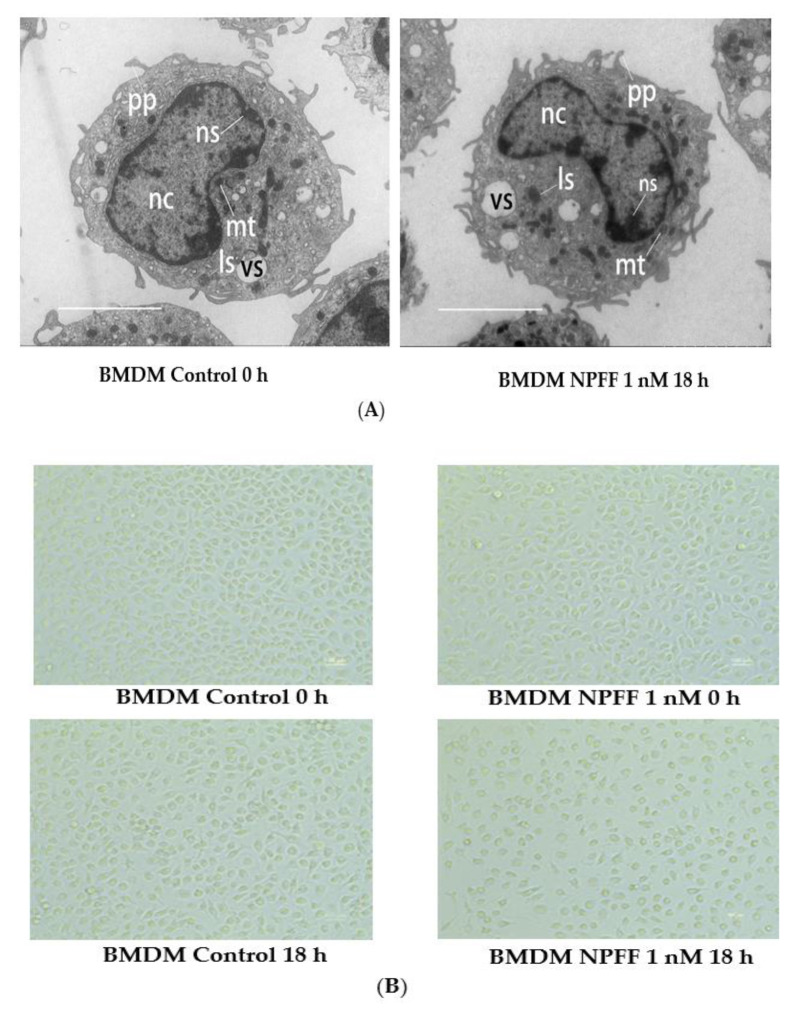

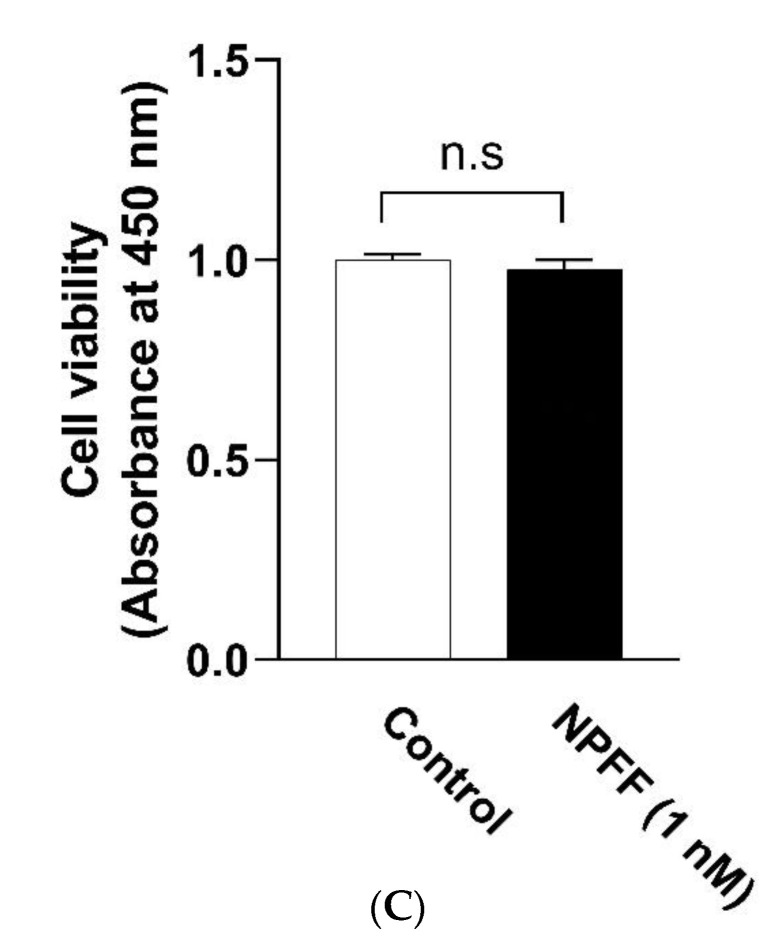
The effect of NPFF treatment on BMDMs. BMDMs were treated with or without NPFF (1 nM) for 18 h, followed by various examinations. (**A**) The detailed structure of the cells was obtained by electron microscopy. Scale bar, 50 μm. Nc = nucleus; ns = nucleolus; mt = mitochondria; vs = vesicle; ls = lysosome; pp = pseudopodia. (**B**) The morphology of the cells was acquired by optical microscope inspection. Scale bar, 100 μm. (**C**) The cell viability was investigated by CCK-8 assay. N.s, no significance. Each test was performed four times in duplicate. The data were shown as the means ± S.E.M. Statistical significance analysis was carried out using the t-test method.

As demonstrated in Figure 2A, the cell morphology of BMDMs was examined with electron microscopy. Subsequently, the purity of BMDMs was detected by flow cytometry with anti-F4/80 and anti-CD11b (double-positive ratio: 96.1%) (Figure S1A). The data from flow cytometry indicated that the double-positive rate of the BMDMs control group (no antibody was added) was 0.059% (left panel of Figure S1A), whereas the double-positive rate of BMDMs (treated with anti-CD11b and anti-F4/80) was 96.1% (right panel of Figure 2B). Hence, these data hinted that the purity of BMDMs was acceptable.

In order to detect the effect of NPFF on BMDMs, the morphological features of BMDMs before and after NPFF exposure were investigated with an optical microscope. The shape of BMDMs was oval before NPFF (1 nM) exposure, whereas the morphology of the cells did not change significantly after NPFF exposure for 18 h (Figure 2B). 

As shown in Figure 2A, NPFF did not exhibit a noticeable effect on the shape of the nucleus of macrophages. The nucleoli of the control group were unevenly distributed under the nuclear membrane of the nucleus. The treatment of NPFF failed to cause significant changes in the number, size, and shape of the nucleolus, hinting that NPFF may not affect a series of processes in the nucleus, including the transcription and processing of ribosomal RNA (rRNA), and the assembly of ribosomal subunits. Also, NPFF did not induce noticeable changes in the number and morphology of vesicles of macrophages, suggesting that NPFF has no significant effect on vesicles-the main organelles responsible for phagocytosis of pathogenic microorganisms in macrophages. Besides, mitochondria and lysosomes in macrophages did not demonstrate significant morphological and quantitative changes due to the treatment of NPFF, indicating that NPFF may not morphologically change the energy metabolism of BMDMs and the activity of degrading cellular contents. However, compared with the control group, NPFF treatment seemed to reduce the number of pseudopods in BMDMs, implying that NPFF may be involved in the migration and phagocytosis of macrophages. 

In addition, NPFF 1 nM treatment for 18 h did not significantly affect the viability of BMDMs (Figure 2C).

### 3.2. Identification of DEGs

**Figure 3:**
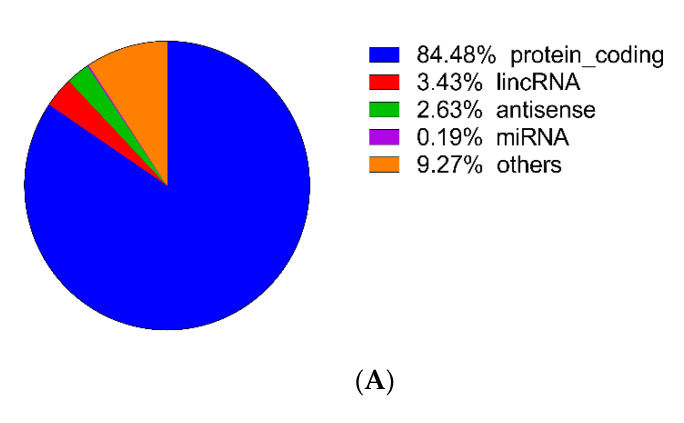

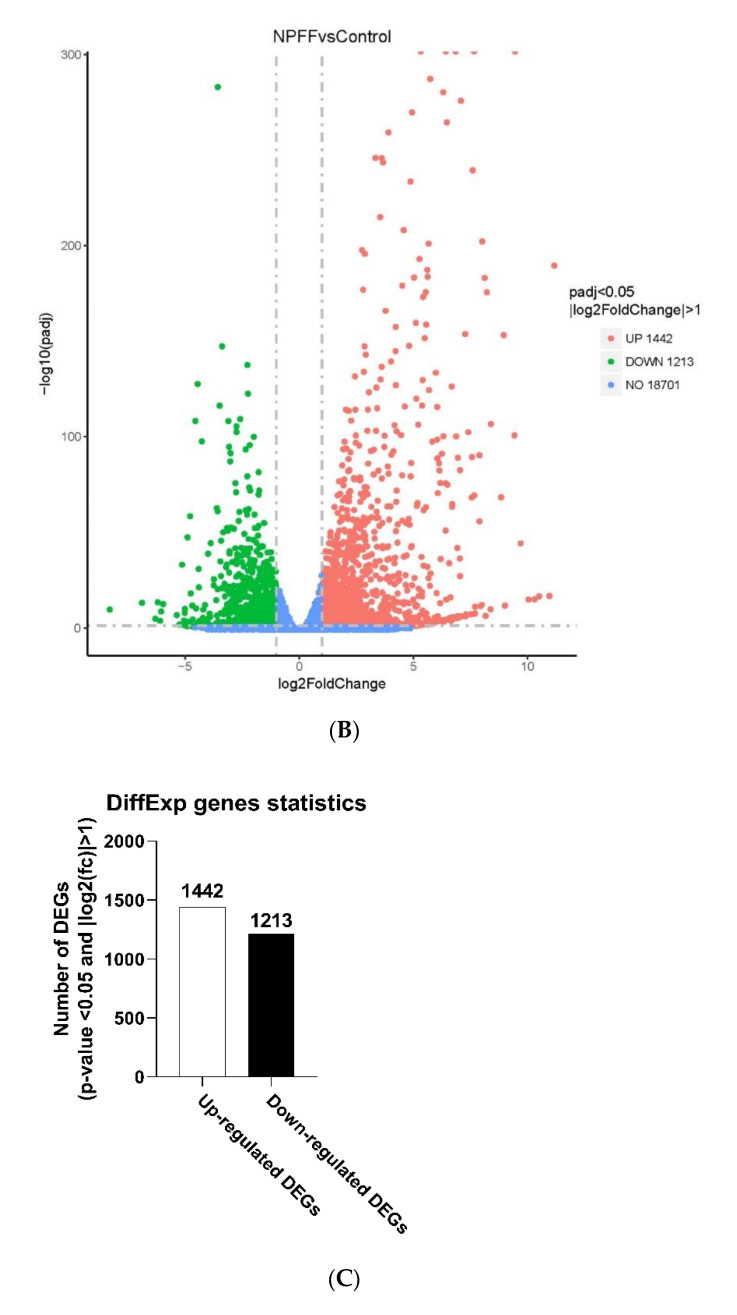

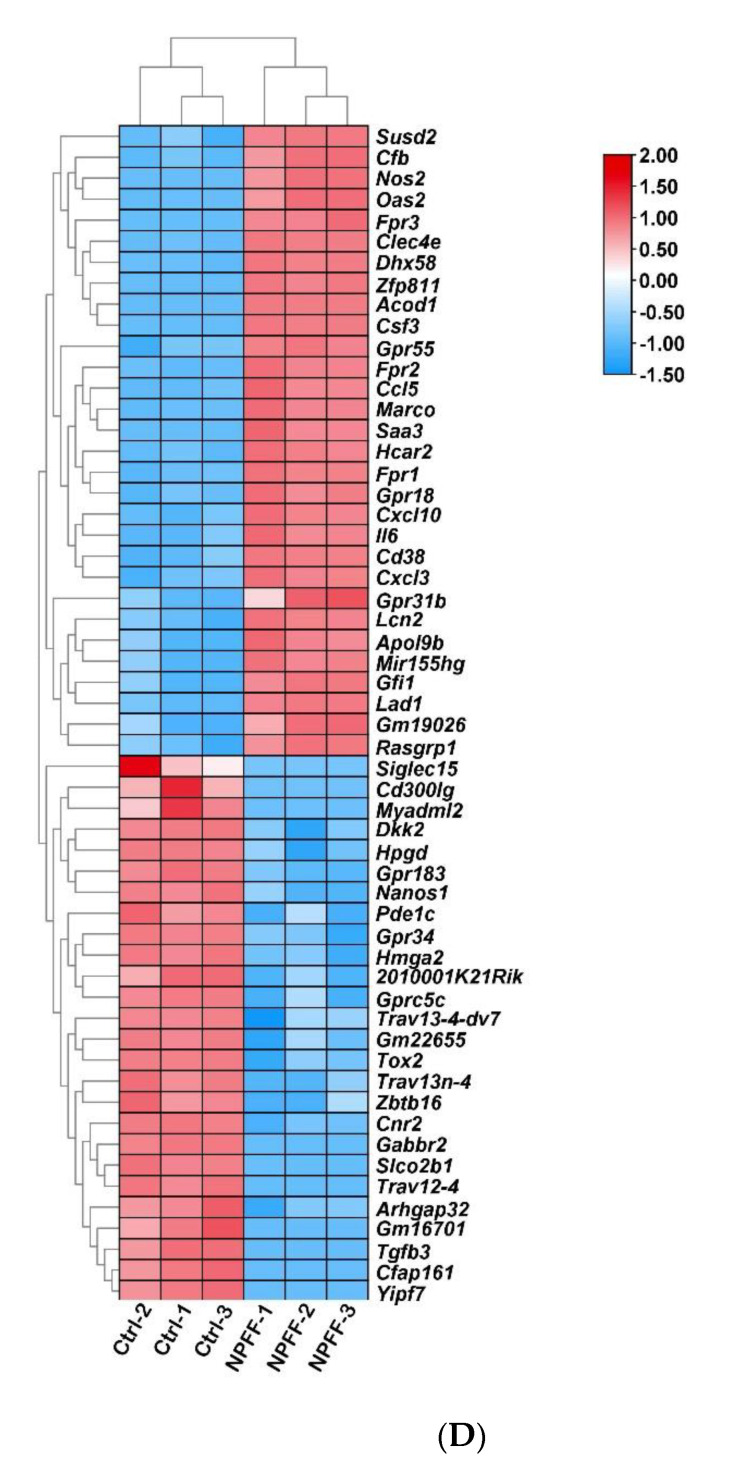
Basic information about RNA-seq results. (**A**) The proportion of differentiated expressed genes (DEGs) (*p*-value < 0.05 and |log2(fc)| > 1). (**B**) Volcano plot of RNA-seq results (green dots: down-regulated genes; red dots: up-regulated genes). (**C**) Differentially expressed genes statistics (*p*-value < 0.05 and |log2(fc)| > 1). (**D**) Heat map of the 48 DEGs with the largest absolute value of FoldChange (24 up-regulated DEGs and 24 down-regulated DEGs) and 8 hub genes. The red indicated the up-regulated genes and blue represented down-regulated genes.

To detect the influence of NPFF on the transcriptome of BMDMs, cell samples were investigated by RNA-seq sequencing (Figure 3A–D, Figure S1B,C and Table S1). The quality control test results demonstrated that our RNA-seq was qualified (Figure S1B,C). A total of 2655 DEGs were acquired, of which 1213 genes were down-regulated and 1442 genes were up-regulated (criteria: *p*-value < 0.05 and |log2(fc)| > 1) (Figure 3A and Table S1). A heatmap and volcano map demonstrated the distribution of genes in each group (Figure 3B,C). Overall, NPFF activated (1442 up-regulated genes) more genes than inhibiting genes (1213 down-regulated genes) on the transcriptional level of BMDMs (Table 2). In addition, NPFF regulated the expression of genes encoding antisense RNA and miRNA in the transcriptome of BMDMs (Tables S2–S5). DEGs activated by NPFF were composed of the following types of genes: protein_coding genes (84.48%) (Table S2), antisense genes (2.63%) (Table S3), lincRNA genes (3.43%) (Table S5), miRNA genes (0.19%) (Table S4), and other genes (9.27%) (Figure 3A).

### 3.3. Functional and Pathway Enrichment Interpretation of DEGs

To investigate the biological meanings of DEGs, a series of approaches were employed to explore the functions of DEGs, such as KEGG, Metascape, and PANTHER. 

The KEGG online approach was employed to explore the enrichment pathways of DEGs. As demonstrated in Table S6, DEGs were involved in the following biological pathways: autoimmune thyroid disease (mmu05320; gene count: 110), allograft rejection (mmu05330; gene count: 46), NOD-like receptor signaling pathway (mmu04621; gene count: 32), and Epstein–Barr virus infection (mmu05169; gene count: 24).

**Figure 4:**
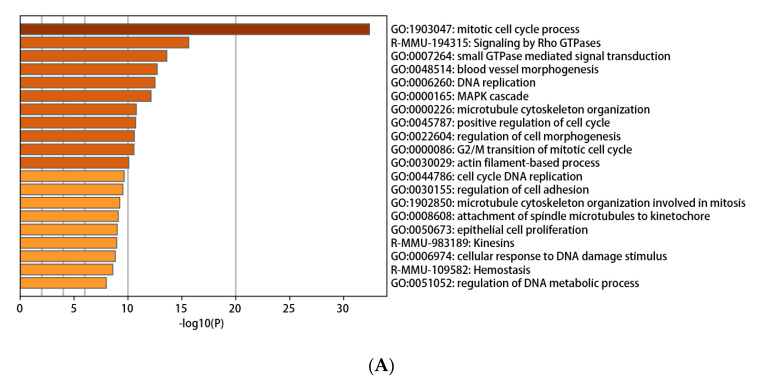

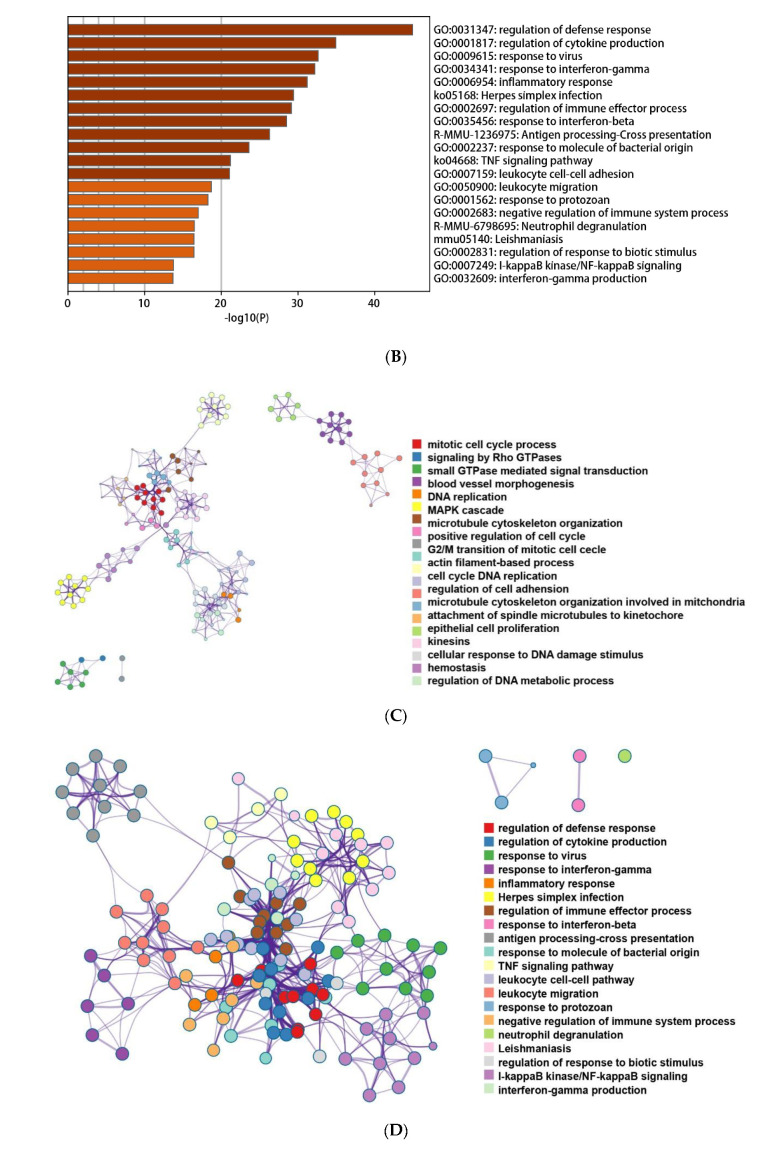

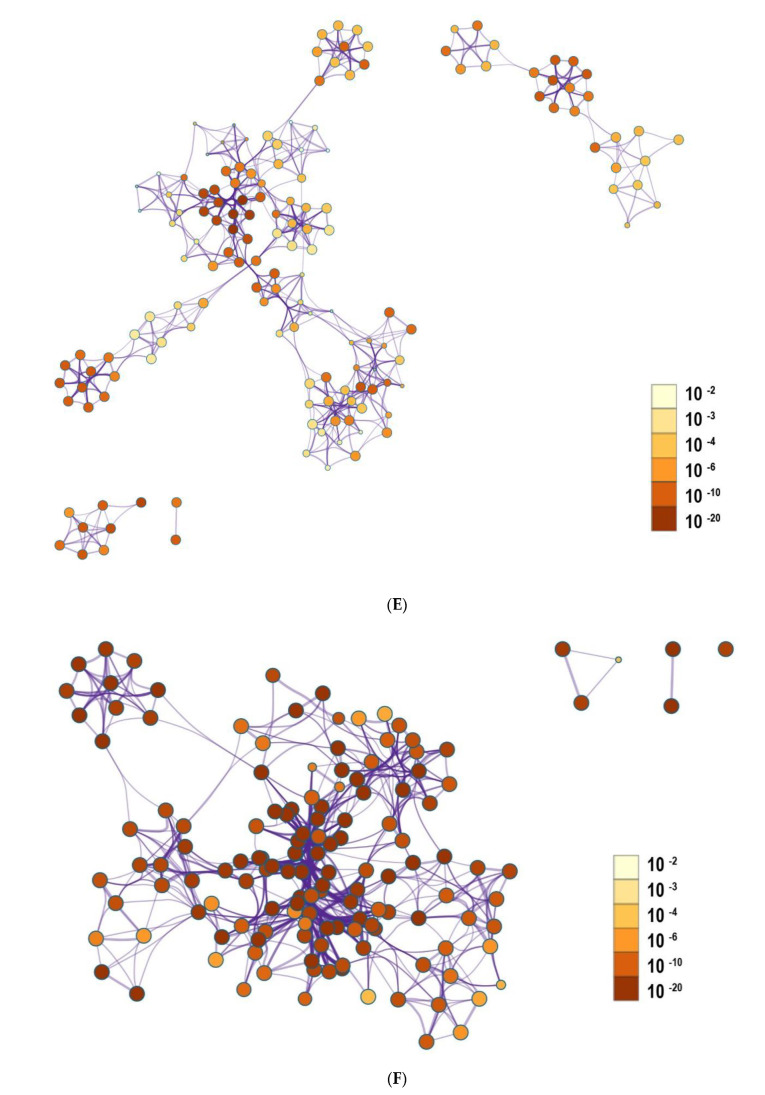
The enrichment analysis data from Metascape. (**A**,**B**) Bar plot of enriched terms (colored by cluster ID) for down-regulated DEGs (**A**) and up-regulated DEGs (**B**). (**C**,**D**) The network of enriched terms (colored by cluster) for down-regulated DEGs (**C**) and up-regulated DEGs (**D**). (**E**,**F**) Network plot of enriched terms (colored by *p*-value) for down-regulated DEGs (**E**) and up-regulated DEGs (**F**).

The Metascape online tool was used to detect the functional enrichment of DEGs. All DEGs were uploaded to Metascape to investigate the enrichment pathways. As shown in Figure 4A,B, enriched pathways provoked by up-regulated DEGs mainly included the regulation of defense response, regulation of cytokine production, response to the virus, and response to interferon-γ. Meanwhile, the down-regulated DEGs stimulated the mitotic cell cycle process, signaling by Rho GTPases, and small GTPase-mediated signal transduction. These enrichment pathways were clustered and connected to various network diagrams (Figure 4C–F).

The online website PANTHER was also used to explore the enrichment processes of DEGs. All DEGs were classified into the following three categories: biological process (BP), molecular function (MF), and cellular compartment (CC). 

**Figure 5:**
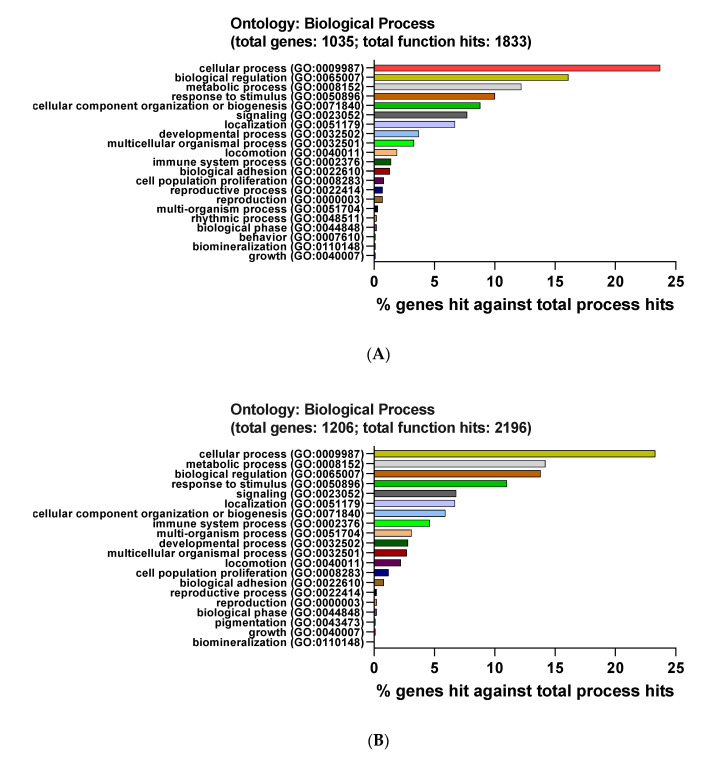

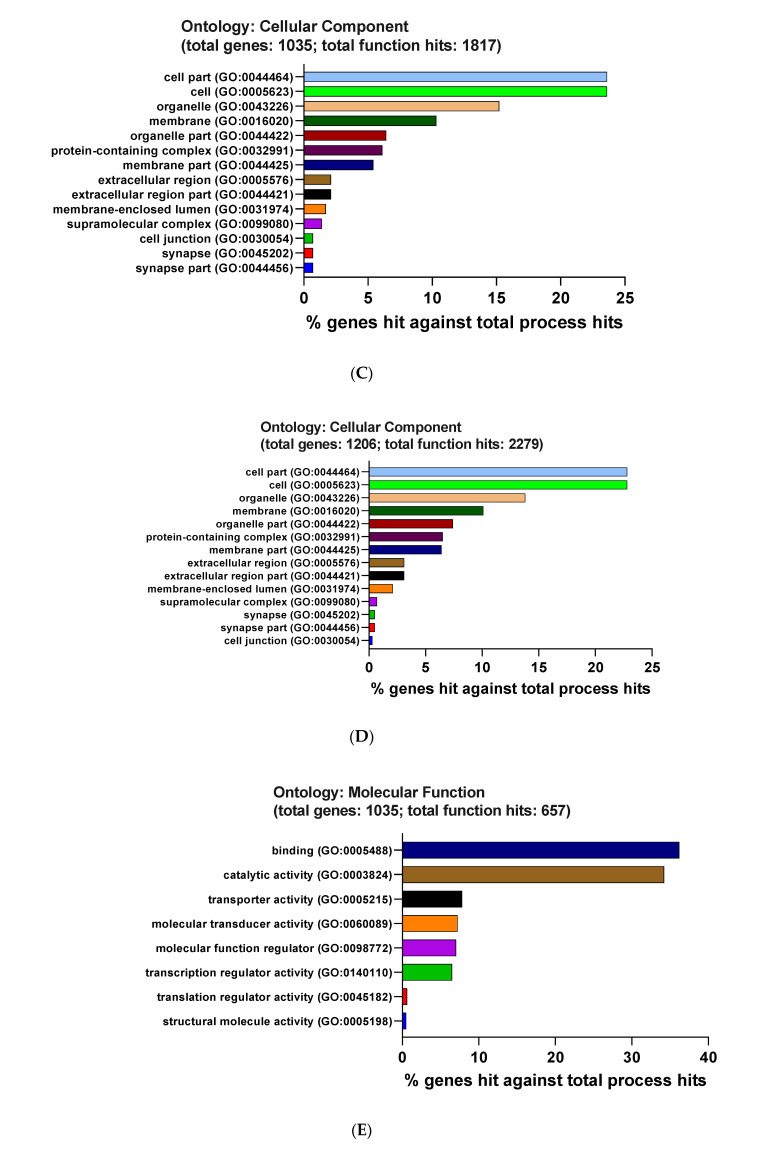

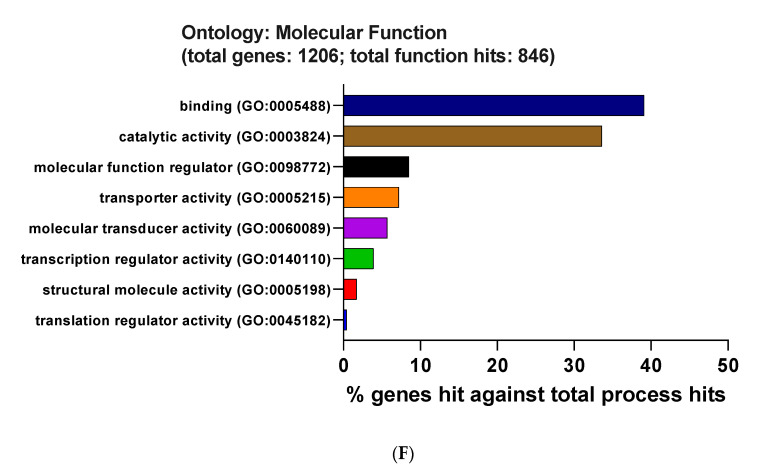
Functional enrichment analysis of DEGs from PANTHER. The DEGs were subjected to Gene Ontology (GO) classification with the PANTHER GO classification. (**A**,**B**) Biological process (BP) of down-regulated (**A**) and up-regulated (**B**). (**C**,**D**) Cellular component (CC) of down-regulated (**C**) and up-regulated (**D**). (**C**) Molecular function (MF) of down-regulated (**E**) and up-regulated (**F**).

As demonstrated in Figure 5A, the DEG-caused biological processes were mainly composed of the following processes: cellular process (GO:0009987, 23.7%), biological regulation (GO:0065007, 16.1%), and metabolic process (GO:0008152, 12.2%). Meanwhile, the biological processes provoked by up-regulated DEGs were cellular process (GO:0009987, 23.3%), metabolic process (GO:0008152, 14.2%), and biological regulation (GO:0065007, 13.8%) (Figure 5B).

For the cellular compartment pathway, the main proteins activated by down-regulated DEGs were cell part (GO:0044464, 23.6%), cell (GO:0005623, 23.6%), and organelle (GO:0043226, 15.2%) (Figure 5C). Besides, up-regulated DEGs activated the following pathways: cell part (GO:0044464, 22.8%), cell (GO:0005623, 22.8%), and organelle (GO:0043226, 13.8%) (Figure 5D). 

As for the molecular function, down-regulated DEGs were involved in the following pathways: binding (GO:0005488, 36.2%), catalytic activity (GO:0003824, 34.2%), and transporter activity (GO:0005215, 7.8%) (Figure 5E). In addition, up-regulated DEGs stimulated following pathways: binding (GO:0005488, 39.1%), catalytic activity (GO:0003824, 33.6%), and molecular function regulator (GO:0098772, 8.5%) (Figure 5F). 

### 3.4. Identification of Hub Genes from Protein–Protein Interaction (PPI) Network

**Figure 6:**
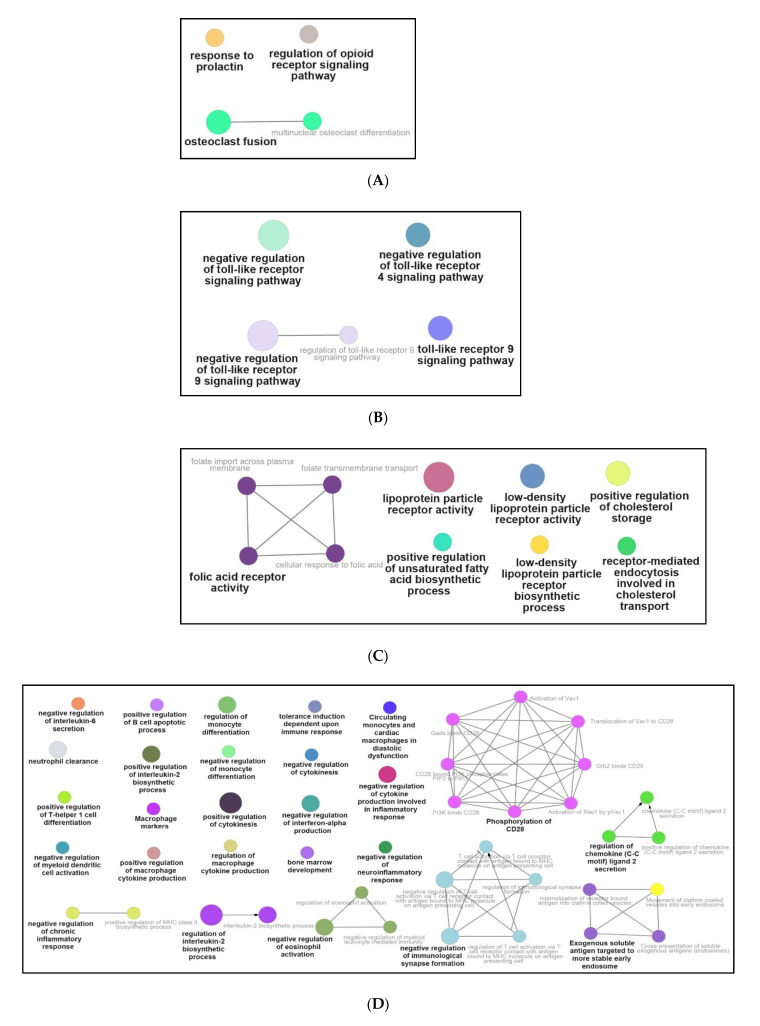

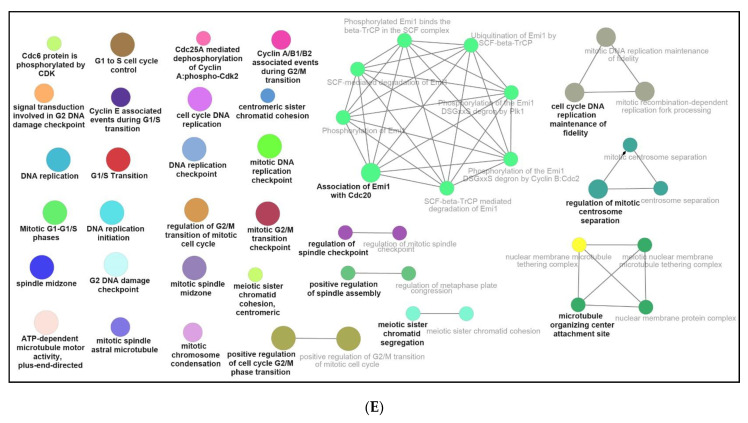
Functional enrichment analysis of down-regulated DEGs from ClueGO. (**A**) Prolactin, opioid signaling pathway, and osteoclast fusion; (**B**) Toll-like receptor signaling pathway; (**C**) fatty acid metabolism; (**D**) inflammation and cytokine; and (**E**) cell checkpoints, cell cycle and cell structure.

To further interpret the protein–protein interactions of DEGs, DEGs were investigated with the online STRING database, followed by visualizing with Cytoscape. Based on the results from Cytoscape’s plug-in cyto-Hubba, a total of eight hub genes (*CNR2, GPR55, GPR18, HCAR2, GPR31B, GPR183, OAS2,* and *DHX58*) were obtained with the highest scores (Table 2 ant Table S7). In addition, the Cytoscape plug-in ClueGO was employed to investigate the functional processes of DEGs. Functional enrichment pathways of down-regulated DEGs from ClueGO were divided into seven different groups (Figure 6): prolactin, opioid signaling pathway, and osteoclast fusion (Figure 6A); Toll-like receptor signaling pathway (Figure 6B); fatty acid metabolism (Figure 6C); inflammation and cytokine (Figure 6D); cell checkpoints; cell cycle and cell structure (Figure 6E); (Figure S2A) cell migration; (Figure S2B) signaling pathways; and (Figure S2C) GPCR and cell channel activity. 

**Figure 7:**
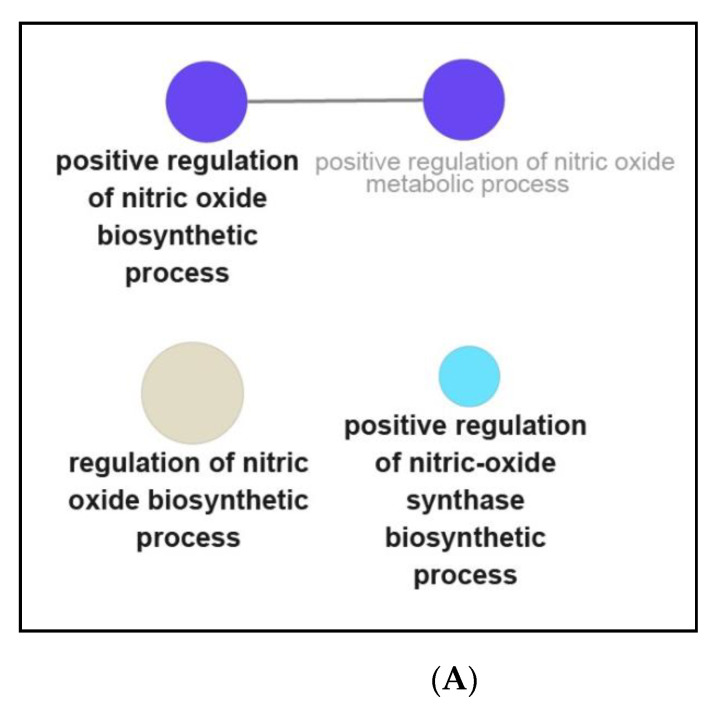

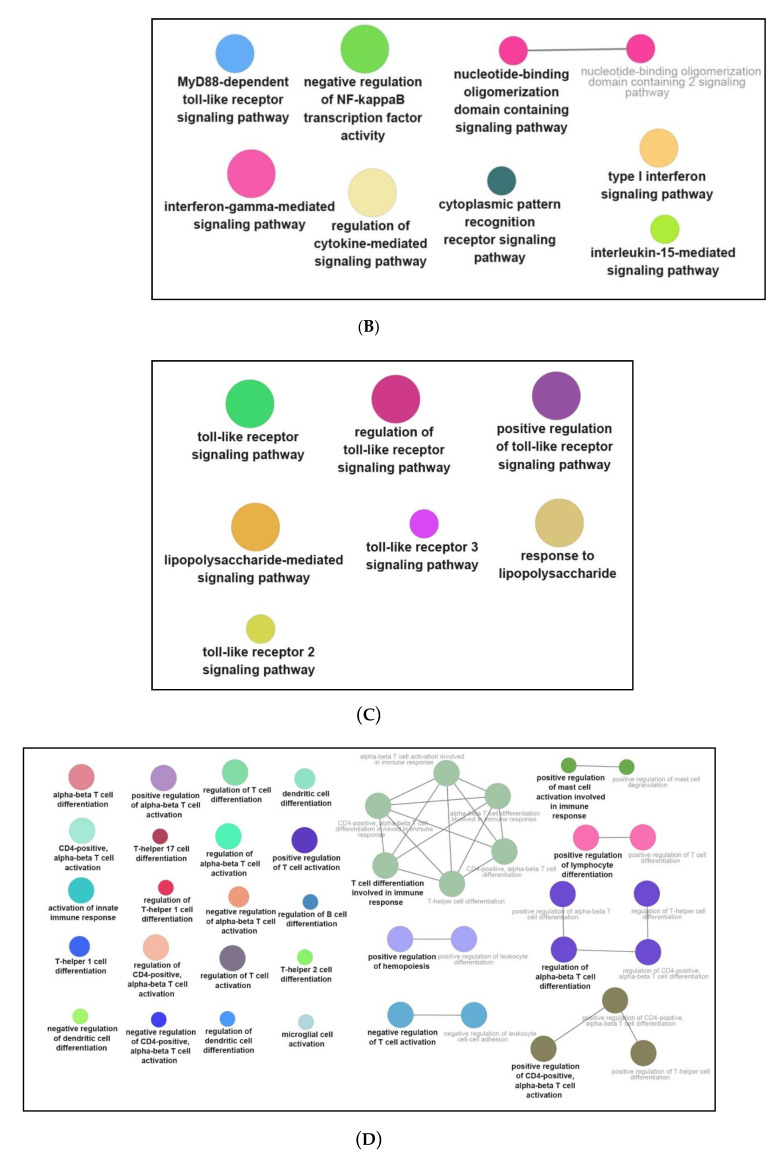

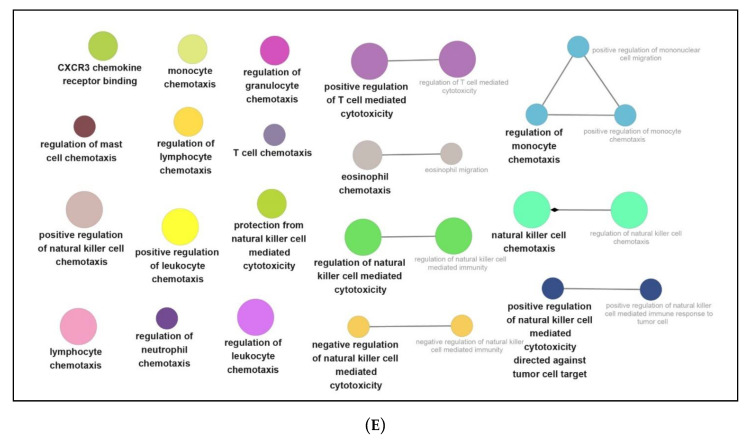
Functional enrichment analysis of up-regulated DEGs from ClueGO. (**A**) Nitric oxide production; (**B**) immune signaling pathways; (**C**) Toll-like signaling pathways; (**D**) activation and differentiation of immune cells; and (**E**) chemotaxis and cytotoxicity of immune cells.

Meanwhile, the up-regulated DEGs activated the following pathways: nitric oxide production (Figure 7A); immune signaling pathways (Figure 7B); Toll-like signaling pathways (Figure 7C); activation and differentiation of immune cells (Figure 7D); chemotaxis and cytotoxicity of immune cells (Figure 7E); (Figure S3A) fatty acid metabolism; (Figure S3B) mitochondrial function; (Figure S3C) proliferation of immune cells; (Figure S3D) cell proliferation; (Figure S3E) cytokine production or activity; (Figure S3F) inflammation and immune activity, and (Figure S3G) other biological activities. Besides, the detailed information and GO analysis of eight hub genes were summarized in Supplementary Tables S6 and S7.

### 3.5. Common Transcription Factors Tied to Genes Down-Regulated by NPFF

In order to analyze the transcription factors of genes regulated by NPFF, TRRUST (version 2) was employed. As shown in Table S8, 23 transcription factors were up-regulated (screening criterion: *p* < 0.05), which included *Nfkb1, Stat1, Jun, Rela, Irf1, Cebpb, Ikbkb, Fos, Irf8, Hdac1, Rel, Ep300, Egr1, Pou2f2, Stat3, Klf4, Ahr, Cebpa, Spi1, Crebbp, Ppara, Sp3,* and *Trp53*. Meanwhile, down-regulated DEGs activated the following 22 transcription factors: *Sp3, Esr1, Atf2, Gtf2i, Esr2, Sp1, Nr0b2, Smad4, Ehmt2, Egr1, Sp4, Ncoa3, Gfi1, Ppard, Ep300, Xbp1, Cebpb, Stat3, Foxo1, Smad3, Pparg,* and *Pitx2* (Table S9).

### 3.6. Verification of Hub Genes with qPCR

**Figure 8:**
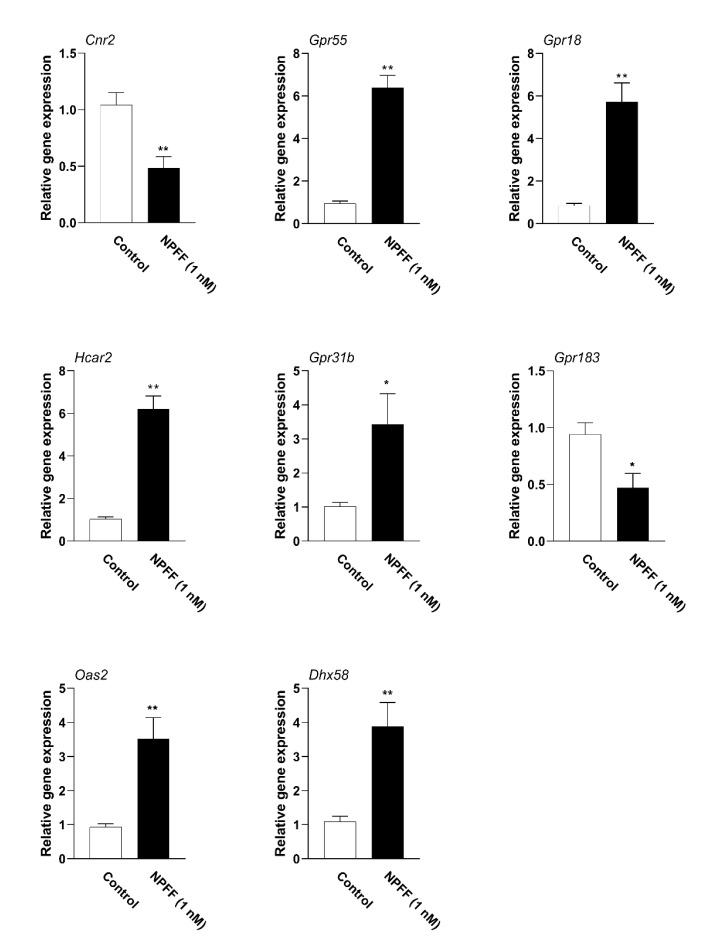
qPCR data for hub genes of BMDMs. BMDMs were incubated with NPFF (1nM) for 18 h, followed by a qPCR examination. Total RNA was isolated, and a qPCR test was conducted to identify eight hub genes. The mRNA level was normalized by the expression of GAPDH. *, significantly different from the control group; * *p* < 0.05; ** *p* < 0.01. Each test was performed three times in duplicate. The data were demonstrated as the means ±S.E.M. Statistical significance analysis was carried out using the t-test method.

Next, qPCR was performed to verify the accuracy of the RNA-seq results. These hub genes were all protein-coding genes (*CNR2, GPR55, GPR18, HCAR2, GPR31B, GPR183, OAS2,* and *DHX58*). As shown in Figure 8, NPFF (1 nM) down-regulated the mRNAs of two hub genes, whereas it up-regulated six hub genes significantly, which were consistent with the RNA-seq results.

### 3.7. Protein Modeling of Hub Proteins

See Supplementary File 1.

### 3.8. Molecular Dynamics Simulation of Hub Proteins

See Supplementary File 1. 

### 3.9. Peptide-Hub Protein Docking

**Figure 9:**
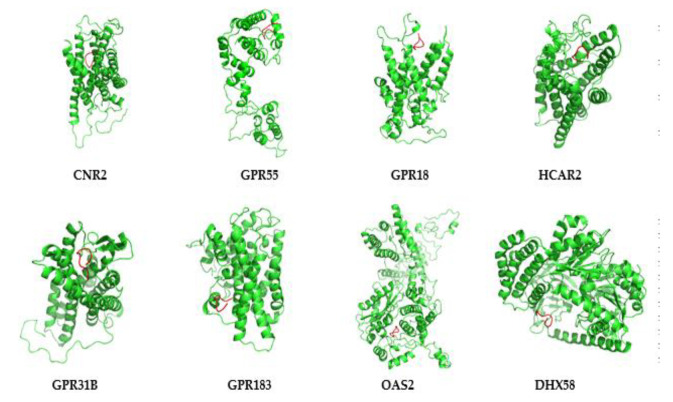
The docking analysis of NPFF and hub proteins. There dimensional structures of NPFF (red)-hub proteins (green) were presented, including CNR2, GPR55, GPR18, HCAR2, GPR31B, GPR183, OAS2, and DHX58. The picture was generated using the Pymol software (Delano, W.L. The Pymol Molecular Graphics System (2002) DeLano Scientific, SanCarlos, CA, USA. http://www.pymol.org, accessed on 11 November 2020).

In order to predict the possible mode of NPFF-hub proteins, the Rosetta program was used to predict the possible dock binding sites of NPFF-hub proteins. As shown in Figure 9, there are two types of binding modes between NPFF and hub protein. Type one: NPFF as a whole entered the region of the N-terminal region of the hub protein, where it is completely embedded in the protein structure (CNR2, GPR55, GPR18, HCAR2, and GPR31B). Type two: NPFF binds to the C-terminal region of the hub protein, which binds to the outside of the protein structure (GPR183, OAS2, and DHX58).

### 3.10. Expression of NPFFR2 on BMDMs

**Figure 10:**
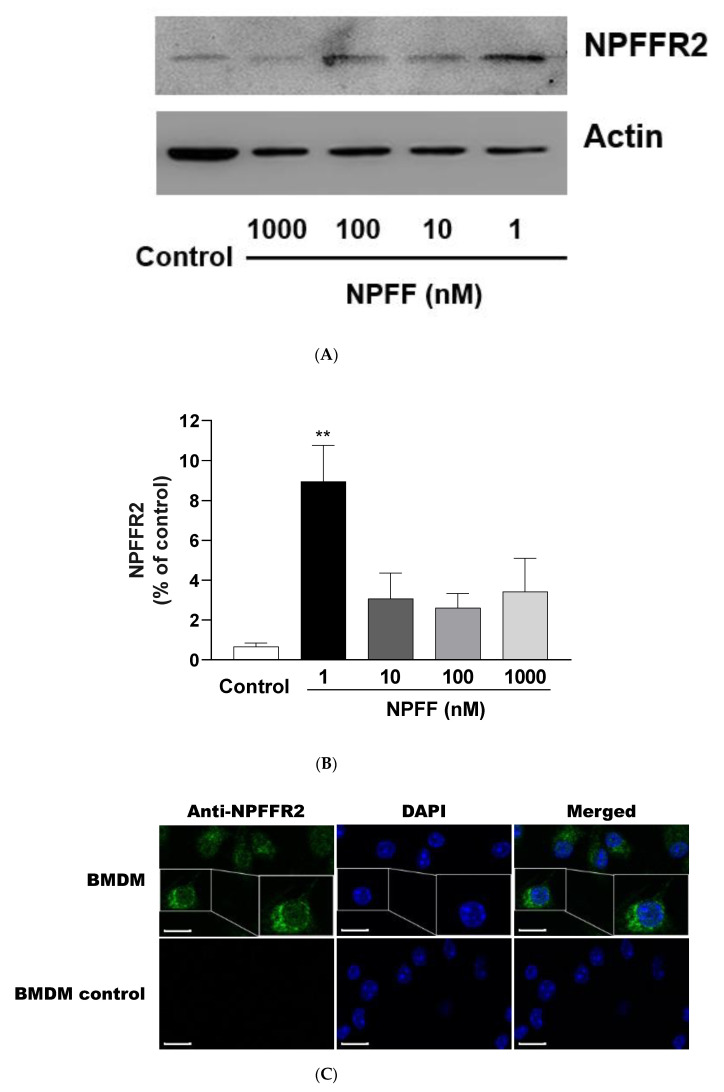
The expression of NPFFR2 protein in BMDMs was examined by Western blot and immunofluorescence staining. (**A**) BMDMs were incubated with NPFF (1 nM) for 18 h, followed by immunoblot analysis of NPFFR2 protein level (n = 3). (**B**) quantification of the bands in (**A**). (**C**) The expression of NPFFR2 in BMDMs was examined by immunofluorescence staining. NPFFR2 stained with anti-NPFFR2 antibody showed green, and the nucleus stained with DAPI showed blue. Scale bar, 10 μm. The data were demonstrated as the means ± S.E.M. *, significantly different from the control group; * *p* < 0.05; ** *p* < 0.01. Statistical significance analysis was conducted using the one-way ANOVA followed by the Tukey post hoc tests approach.

The expression of NPFFR2 was detected in BMDMs by Western blot and immunofluorescence stain (Figure 10). As demonstrated in Figure 10C, NPFFR2 protein was expressed on the cell membrane. Interestingly, anti-NPFFR2 signals are present even in some cytoplasmic regions of BMDMs. In addition, BMDMs showed no signals with the IgG control. Compared with the control group, NPFF 1 nM treatment for 18 h caused a significant increase in the expression of NPFFR2 protein (Figure 10A,B).

## 4. Discussion

### 4.1. The Effect of NPFF on the Morphology and Viability of BMDMs

The effect of NPFF on the viability of macrophages is a question worthy of concern. Our previous studies show that NPFF can effectively enhance the viability of RAW 264.7 cells, a tumor-derived macrophage cell line [31]. However, in this study, NPFF (1 nM, 18 h) did not significantly change the viability of BMDMs (Figure 2C). As for the different activities of NPFF on these two macrophage models (RAW 264.7 and BMDMs), we speculate that the following factors may be worth considering.

(1) The difference in cell lines may be responsible for the regulation of cell viability by NPFF. On the one hand, RAW 264.7 is a leukemia-derived monocyte/macrophage-like cell, so the RAW 264.7 cell line has the characteristics of both tumor cells and macrophages in terms of cytological behavior [57]. On the other hand, BMDMs belong to the non-reproductive cell group, which has some unique cytological characteristics, including the ability to be kept alive for 2–3 weeks under suitable conditions, is mainly used for primary culture, and is difficult to keep alive for a long time [1,4]. Therefore, the above essential cytological characteristics may be responsible for the influence of NPFF on them.

(2) The effect of NPFF on the activity of BMDMs may be concentration-dependent. The regulation of NPFF on BMDMs may also have some characteristics of the structure–activity relationship. However, in our current experimental system, we do have difficulties in detecting the effects of NPFF in a wide range of concentrations on BMDMs. In the present study, we aim to explore the regulatory role of NPFF on the transcriptomic profiles of BMDMs, hoping to provide clues for NPFF to regulate the immune response controlled by BMDMs.

(3) The unique physiological functions of macrophages may also be a problem worth considering. As a critical cell type in the immune system, macrophages play a fundamental role in the entire neuro–endocrine–immune network [3,4]. In a physiological environment, macrophages can be induced to differentiate into multiple cell types, which means that macrophages are in a dynamic equilibrium [5]. Therefore, NPFF, a neuropeptide with hormone-like effects, may regulate BMDMs in multiple ways, which needs to be revealed by further experiments.

### 4.2. NPFF Regulated Different Functional Enrichment Pathways of BMDMs

In this study, NPFF regulated the gene expression profile of BMDM cells, which provided clues to understand various experimental results of previous reports.

NPFF affected the opioid signaling pathway (Figure 6A), which provided clues to the reported regulation of opioid analgesic activity by the NPFF system [58,59]; NPFF inhibited osteoclast activity (Figure 6A), which was consistent with the report that NPFF suppresses the differentiation of monocytes into osteoclasts [60,61]; NPFF regulated fatty acid metabolism (Figure 6C), which provided basis for the modulatory function of NPFF on lipid metabolism [22]; NPFF adjusted the cell checkpoints of macrophages (Figure 6E), which was consistent with the recent study that NPFF modulates the cell checkpoints-related gene (*PDL1*) of RAW 264.7 macrophages [33]; NPFF modulated the Toll-like receptor signaling pathway (Figure 6B and Figure 7C), which might explain the previous report that NPFF inhibits the TLR4-induced inflammatory response of macrophages [31,32]. Besides, NPFF regulated the nitric oxide signaling pathway of macrophages (Figure 7A), which might provide a basis for the previous report that NPFF suppresses the nitric oxide level of macrophages [31,32].

In addition, NPFF had a regulatory effect on the inflammation and immune-related signal pathways of macrophages (Figure 6D and Figure 7B,D,E), indicating that NPFF may be deeply involved in the immune regulation activities of macrophages. Therefore, given that macrophages are widely anticipated in various physiological processes in the body [2], NPFF may be widely involved in multiple physiological processes mastered by macrophages.

### 4.3. Common Transcription Factors Tied to NPFF-Regulated DEGs in BMDMs

In this study, NPFF caused the up-regulation and down-regulation of many genes (up-regulated 1442 DEGs *vs* down-regulated 1213 DEGs) (criteria: *p*-value < 0.05 and log2(fc) > 1). In order to capture the effects of these DEGs on the gene expression of macrophages at the transcription factor level, the up-regulated and down-regulated DEGs were subject to TRRUST (version 2) for transcription factor analysis, respectively.

As shown in Table S8, NPFF activated a series of commonly used transcription factors, including *Nfkb1* (nuclear factor of kappa light polypeptide gene enhancer in B cells 1, p105), *Stat1* (signal transducer and activator of transcription 1), *Ep300* (E1A binding protein p300), *Stat3* (signal transducer and activator of transcription 3), and *Cebpa* (CCAAT/enhancer binding protein (C/EBP), α). These data suggested that NPFF may activate the expression of related gene networks controlled by these transcription factors. In addition, NPFF also stimulated the activity of some immune-related transcription factors, including *Irf1* (interferon regulatory factor 1) and *Irf8* (interferon regulatory factor 8), implying that the immune-related gene signaling pathways controlled by these transcription factors in macrophages may be affected by NPFF.

It is worth noting that NPFF also inhibited the activity of a series of commonly used transcription factors (Table S9), including *Sp3* (trans-acting transcription factor 3), *Atf2* (activating transcription factor 2), *Gtf2i* (general transcription factor II I), *Sp1* (trans-acting transcription factor 1), *Nr0b2* (nuclear receptor subfamily 0, group B, member 2), *Sp4* (trans-acting transcription factor 4), *Ncoa3* (nuclear receptor coactivator 3), *Ep300* (E1A binding protein p300), *Cebpb* (CCAAT/enhancer binding protein (C/EBP), β), and *Stat3* (signal transducer and activator of transcription 3), suggesting that NPFF inhibited the gene expression network controlled by these transcription factors. Interestingly, *Stat3* and *Ep300* were activated by both up-regulated and down-regulated DEGs, hinting that these two transcription factor networks were deeply involved in the regulation of BMDM by NPFF.

In addition, our recent work on the effect of NPFF on the transcriptome of RAW 264.7 cells also showed that *Stat3* is the “driver” of NPFF regulating gene expression in RAW 264.7 cells [33]. Taken together, *Stat3* may be a universal “driver” rather than a “responder” for NPFF to regulate gene expression in macrophages (RAW 264.7 and BMDMs).

### 4.4. The Concentration of NPFF in the Experimental System for High-Throughput Sequencing

In a series of studies using high-throughput sequencing methods to investigate NPFF’s gene expression profiles of various types of cells, the concentration of NPFF was around 1 nM. Waqas team treated mouse 3T3-L1 preadipocytes and J774A.1 macrophages with NPFF 1 nM for 18 h and detected the changes in the gene expression profile of these cells [22,62]. Very recently, our group also applied the same treatment (1 nM, 18 h) to explore the influences of NPFF on the gene expression profile of mouse macrophages RAW 264.7 [33]. Hence, in the present study, BMDMs were treated with 1 nM NPFF for 18 h and were subsequently subject to RNA-seq examination.

### 4.5. Possible Modes of Interaction between NPFF and Hub Proteins

Limited by the current experimental conditions, we have difficulties in using biological experiments to verify the mechanism of action of NPFF on hub proteins. We speculate that NPFF may affect the expression of hub protein in the following ways. (1) NPFF regulates the gene expression of hub protein by binding to NPFFR2; (2) NPFF regulates the expression of a series of differential genes, which in turn affects related transcription factors and ultimately regulates the expression of hub proteins; (3) NPFF directly binds to hub protein to exert biological activities.

In our present study, with the help of molecular simulation methods, the possible binding modes between NPFF and hub proteins were predicted. There are two main ways to bind NPFF to the hub protein. (1) NPFF was embedded into the spatial structure of the hub protein and bound to certain regions; (2) NPFF bound to the outer regions of the hub proteins. (Figure 9). However, it should be noticed that the actual binding modes between NPFF and other proteins still depends on solid evidence from biochemistry and structure–activity relationship studies.

### 4.6. Expression of NPFFR2 on BMDMs

Recently, a series of studies have shown that NPFFR2 is expressed on various macrophages. Waqas et al. show that human and mouse adipose tissue macrophages (ATMs) expressed NPFFR2, which increases after interleukin-4 (IL-4) treatment [22]. In our recent work, NPFFR2 is expressed on the cell membrane of the mouse macrophage cell line RAW 264.7 [33]. In the present study, NPFFR2 was found to be expressed on the cell membrane of BMDM (Figure 10C). Moreover, NPFF 1 nM significantly activated the expression of NPFFR2 protein (Figure 10A,B). Taken together, NPFFR2 is expressed on a variety of macrophages, suggesting that NPFFR2 may be deeply involved in various physiological activities controlled by macrophages.

It is worth noting that NPFF also seems to affect the expression of Actin protein in BMDMs, as NPFF exposure caused a decrease in actin intensities upon a decrease in NPFF concentration (Figure 10A). In our opinion, the following points could be considered.

(1) NPFF may affect the cytoskeleton protein Actin. NPFF is likely to penetrate the cell membrane and act on the protein Actin directly or indirectly, which ultimately causes changes in the cytoskeleton.

(2) The interaction between NPFFR2 and Actin. Since the discovery of NPFF and NPFFR2, NPFFR2 has been studied as a membrane protein of the GPCR family. However, considering the universality of various protein networks in cells, protein–protein interaction (PPI) may also exist between the membrane protein NPFFR2 and the cytoskeletal protein Actin which is distributed adjacent to the cell membrane. Moreover, our immunofluorescence data showed that there was also a small amount of positive signal of NPFFR2 in the cytoplasm of BMDMs near the cell membrane (Figure 10C), which provided the possibility for NPFFR2 to affect Actin. In summary, the possible interaction between NPFFR2 and cytoskeleton Actin is needed to be revealed by subsequent experiments.

### 4.7. The Effects of Neuropeptides on Immune Cells

Recently, the regulation of the vitality of macrophages by neuropeptides has attracted increasing attention. Using human peripheral polymorphonuclear neutrophils and murine polymorphonuclear neutrophils as models, the B. Kofler group systematically explored Galanin, a 29-amino acid neuropeptide, to regulate immune cells [63]. Their data showed that galanin and its three receptors (GAL1-GAL3) have different expression characteristics on neutrophils. GAL1 receptor is not expressed on all tested neutrophils, while GAL2 receptor is naturally expressed in both human and murine polymorphonuclear neutrophils. In particular, the GAL3 receptor is exclusively expressed in murine bone marrow polymorphonuclear neutrophils. In functional experiments, galanin significantly enhanced the response of polymorphonuclear neutrophils of both species to interleukin-8.

Given galanin has shown both pro- and anti-inflammatory activities in immune cells and inflammatory animal models. B. Kofler’s group recently explored the regulation of galanin on human and murine polymorphonuclear neutrophils [64]. Their data shows that galanin and its receptors are deeply involved in the polarization process of macrophages as galanin can activate different immune cell types and regulate the production of essential chemokines/cytokines in macrophages.

It is worth noting that in the field of NPFF, the study of NPFF’s regulation of macrophages has just started. The way in which the immunomodulatory peptide galanin regulates immune cells provides us with valuable clues for subsequent exploration of NPFF’s regulation of macrophages.

In summary, the regulation of neuropeptides (such as galanin and NPFF) on macrophages may be more complicated than we previously assumed. These data provide a cytological basis for neuropeptides to participate in the macrophage regulatory network. Hence, the regulation of neuropeptides on macrophages needs to be further explored, which may pave the way for revealing the profound and complex regulatory functions of neuropeptides in the neuroimmune system.

## 5. Conclusions

Our work shows that, rather than significantly inhibiting the expression of immune-related gene transcriptome on RAW 264.7 cells, NPFF simultaneously up-regulated and down-regulated the gene expression profile of a large number of BMDMs, indicating that NPFF may profoundly affect a variety of cellular processes governed by BMDMs. Our work provides transcriptomics clues for exploring the influence of NPFF on the biological functions of BMDMs.

## Figures and Tables

**Table 1:** Primers for qPCR analysis.

Genes	Primers	Sequences (5′ to 3′)	Products (bp)
*Gapdh*	Forward	TGTGTCCGTCGTGGATCTGA	150
	Reverse	TTGCTGTTGAAGTCGCAGGAG	
*Cnr2*	Forward	GCTCTGTTCATATCAAATC	84
	Reverse	ATCCTTTAGTATCATCTCTG	
*Gpr55*	Forward	CTCTGTTCTTTATACTCCTAC	133
	Reverse	GGTTCTTCTGCTTCATAC	
*Gpr18*	Forward	ATTACCTTCGCAGTGTTC	90
	Reverse	CTCTGACTCAAAGCATCTC	
*Hcar2*	Forward	CTGTCCACCTCCTCTATAC	90
	Reverse	GCCACCTGAAGTTGTAAC	
*Gpr31b*	Forward	CTGTCTACCTGTTCAACC	76
	Reverse	AGATAGAAGGCAGCAAAG	
*Gpr183*	Forward	TGCTGCGATTCTCTGTAATG	84
	Reverse	GTGCTTAGGAACTTAGGAAGAC	
*Oas2*	Forward	CATTGTTGTGTTCCTCTC	96
	Reverse	AATTCTTCTAACTGCTTCTG	
*Dhx58*	Forward	AACCAAATCCACCAACAAC	75
	Reverse	CACTTGCTGCTCATACATC	

**Table 2:** Hub genes of NPFF-treated BMDMs.

Gene Name Ensembl ID	Species Gene Type	Location Length	Expression Changes (NPFF vs. Control)	Function	Refs
*Cnr2* (Cannabinoid Receptor 2) (ENSEMBL: ENSG00000188822)	Mus musculus Protein coding	Chr 4 (4084 bp)	Down-regulated	Is the receptor for cannabinoids, and is involved in the diseases such as polyarticular juvenile idiopathic arthritis, and cannabis abuse. Besides, it regulates the GPCR pathway and peptide ligand-binding receptors.	[49]
*Gpr55* (G protein-coupled receptor 55) (ENSG00000135898)	Mus musculus Protein coding	Chr 1 (2550 bp)	Up-regulated	Involved in the cannabinoid receptor pathway, GPCR signaling pathway, and diseases include cannabis abuse and lysinuric protein intolerance.	[50]
*Gpr18* (G protein-coupled receptor 18) (ENSG00000125245)	Mus musculus Protein coding	Chr 14 (1424 bp)	Up-regulated	Involved in the GPCR signaling pathways and GPCRs.	[51]
*Hcar2* (Hydroxycarboxylic Acid Receptor 2) (ENSG00000182782)	Mus musculus Protein coding	Chr 5 (1947 bp)	Up-regulated	Involved in the pathways such as peptide ligand-binding receptors and GPCR signaling pathway, and is closely associated with diseases include diversion colitis and pellagra.	[52]
*Gpr31b* (G Protein-Coupled Receptor 31) (ENSG00000120436)	Mus musculus Protein coding	Chr 17 (960 bp)	Up-regulated	Anticipates in the process of ischemia, and regulates the pathways of free fatty acid receptors and GPCR.	[53]
*Gpr183* (G Protein-Coupled Receptor 183) (ENSG00000169508)	Mus musculus Protein coding	Chr 14 (2942 bp)	Down-regulated	Involved in the immune response upon Epstein–Barr virus infection of primary B lymphocytes and regulates the function of thrombin receptor.	[54]
*Oas2* (2′-5′-Oligoadenylate Synthetase 2) (ENSG00000111335)	Mus musculus Protein coding	Chr 5 (3957 bp)	Up-regulated	Modulates the innate immune system and interferon γ signaling and is involved in disorders such as microphthalmia with limb anomalies and tick-borne encephalitis.	[55]
*Dhx58* (DExH-Box Helicase 58) (ENSG00000108771)	Mus musculus Protein coding	Chr 11 (2427 bp)	Up-regulated	Involved in the diseases such as rabies and measles and regulates several pathways include IFN-α/β pathways and innate immune system.	[56]

## Data Availability

Not applicable.

## Supplementary Materials

The following are available online at https://www.mdpi.com/article/10.3390/genes12050705/s1, Supplementary file 1_Materials Manuscript; Figure S1: Information of RNA-seq; Figure S2: Protein–protein interaction for down-regulated DEGs; Figure S3: Protein–protein interaction for up-regulated DEGs; Figure S4: Ramachandran plot; Figure S5: 3D protein alignment; Figure S6 RMSD; Figure S7 RMSF; Figure S8: Gyration radius; Figure S9: The 3-D structure of hub proteins. Table S1_NPFFvsControl_deg_all; Table S2: Details of DEGs-protein coding; Table S3: Details of DEGs-antisense; Table S4 Details of DEGs-miRNA; Table S5: Details of DEGs-lincRNA; Table S6: KEGG pathway; Table S7: GO analysis of hub genes; Table S8: transcriptor factors-up DEGs; Table S9: transcriptor factors-down DEGs; Table S10: Proteins modelling; Table S11: Ramachandran plot analysis; Table S12_RMSD-RMSF-Rg.
