# Supplementary material for: Transcriptomic Changes in Mouse Bone Marrow-Derived Macrophages Exposed to Neuropeptide FF
Source: Genes (Basel). 2021 May 9;12(5):705. doi: 10.3390/genes12050705 (PMC8151073; doi:10.3390/genes12050705)
Supplement: Supplementary file 1 [file genes-12-00705-s001.zip › genes-1147651-supplementary/Figure S1_Information of RNA-seq.pdf]

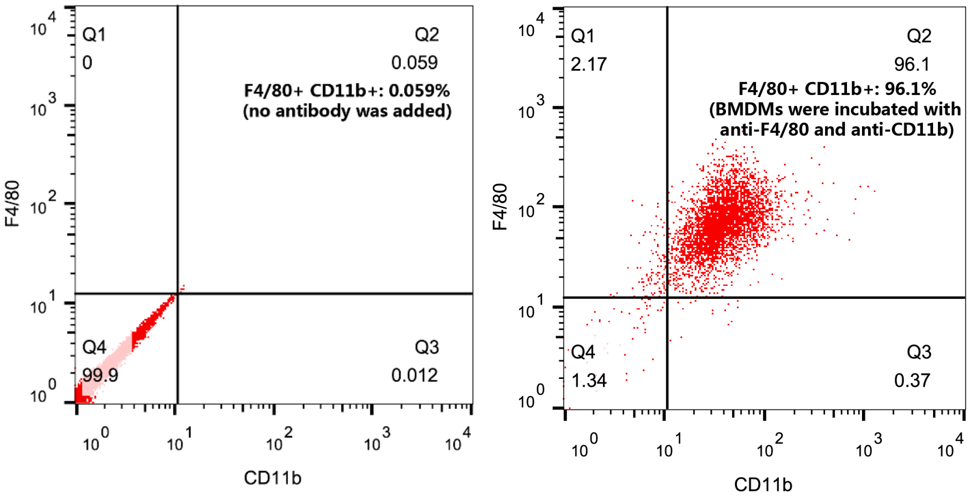

Q1
0
Q2
0.059
F4/80+ CD11b+: 0.059%
(no antibody was added)
Q4
99.9
Q3
0.012
F4/80
CD11b
Q1
2.17
Q2
96.1
F4/80+ CD11b+: 96.1%
(BMDMs were incubated with
anti-F4/80 and anti-CD11b)
Q4
1.34
Q3
0.37

(A)

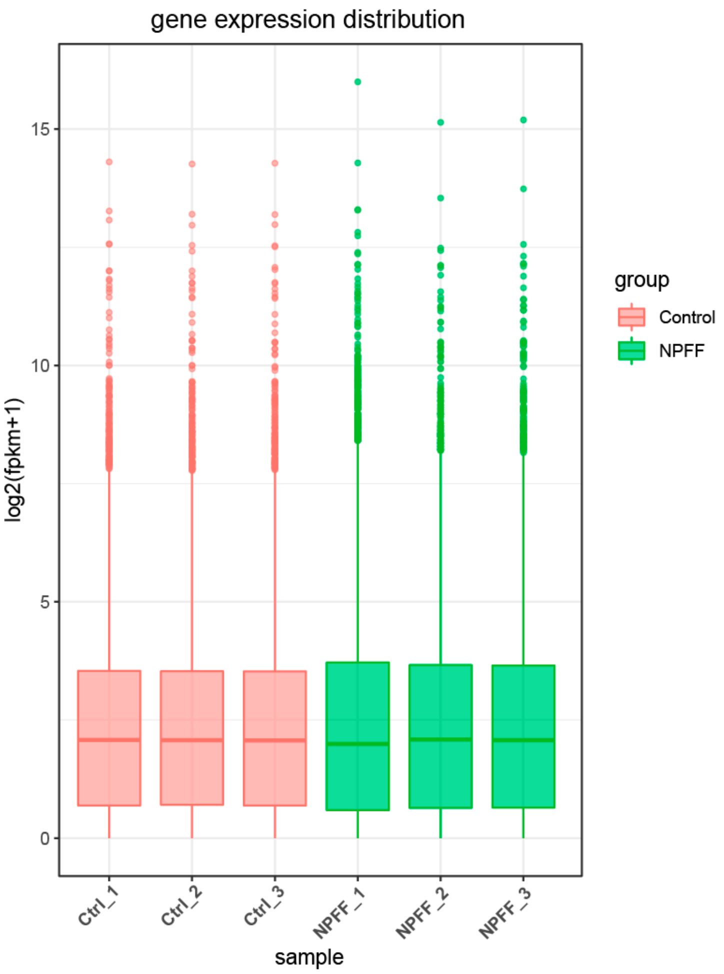

gene expression distribution
log2(fpkm+1)
sample
group
Control
NPFF
Ctrl_1
Ctrl_2
Ctrl_3
NPFF_1
NPFF_2
NPFF_3

(B)

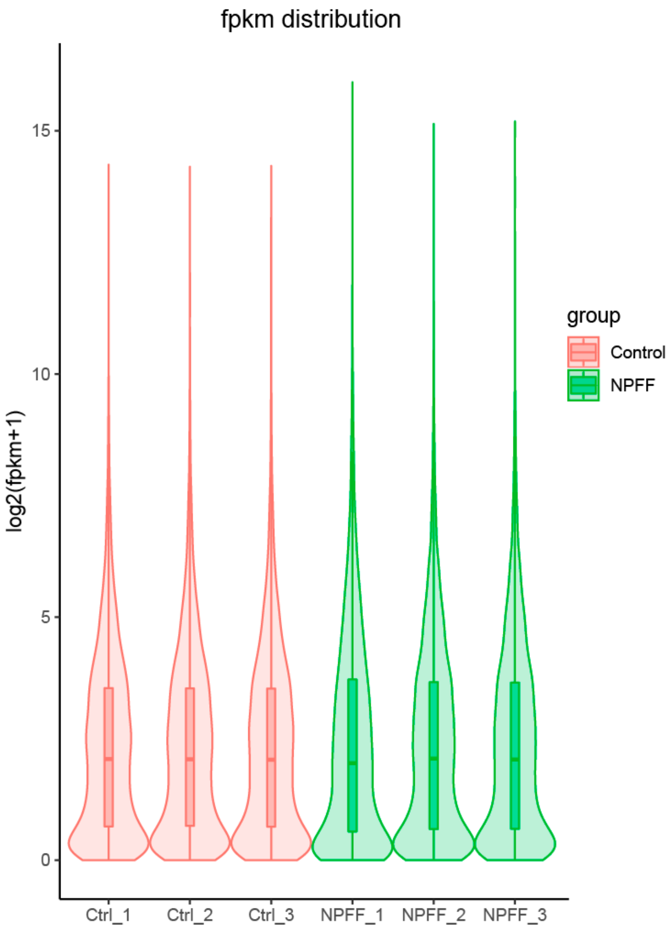

(C)

Figure S1. Information of the FACS and RNA-seq detection. (A) BMDMs were analyzed by FACS with anti-F4/80 and anti-CD11b (double-positive ratio: 96.1%). (B) The boxplot of gene expression distribution. (C) Violin plot.
