# Supplementary material for: Transcriptomic Changes in Mouse Bone Marrow-Derived Macrophages Exposed to Neuropeptide FF
Source: Genes (Basel). 2021 May 9;12(5):705. doi: 10.3390/genes12050705 (PMC8151073; doi:10.3390/genes12050705)
Supplement: Supplementary file 1 [file genes-12-00705-s001.zip › genes-1147651-supplementary/Figure S2 DEG PPI cluego-new-down-all.pdf]

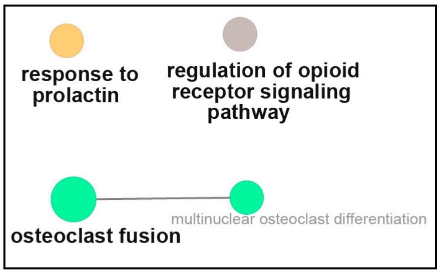
response to prolactin
regulation of opioid receptor signaling pathway
osteoclast fusion
multinuclear osteoclast differentiation

(A)

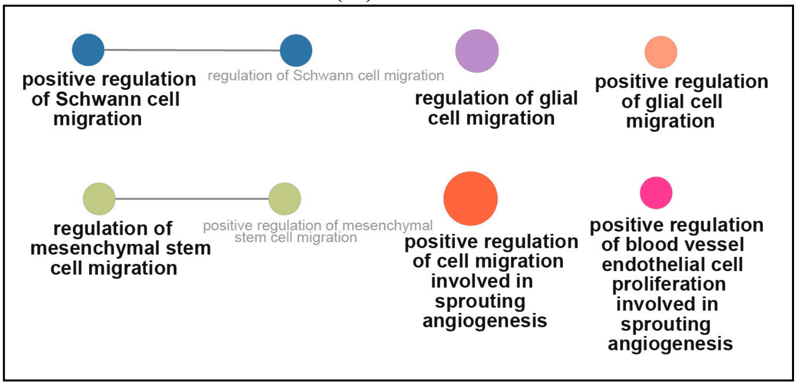
positive regulation of Schwann cell migration
regulation of Schwann cell migration
regulation of glial cell migration
positive regulation of glial cell migration
regulation of mesenchymal stem cell migration
positive regulation of mesenchymal stem cell migration
positive regulation of cell migration involved in sprouting angiogenesis
positive regulation of blood vessel endothelial cell proliferation involved in sprouting angiogenesis

(B)

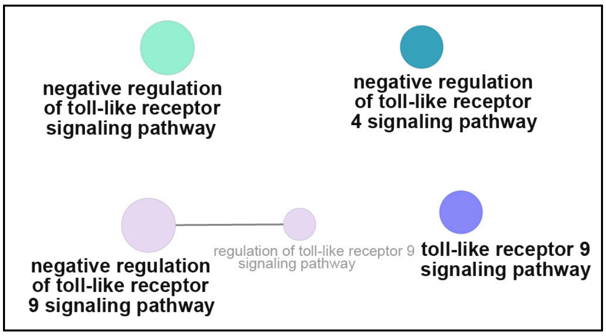
negative regulation of toll-like receptor signaling pathway
negative regulation of toll-like receptor 4 signaling pathway
negative regulation of toll-like receptor 9 signaling pathway
regulation of toll-like receptor 9 signaling pathway
toll-like receptor 9 signaling pathway

(C)

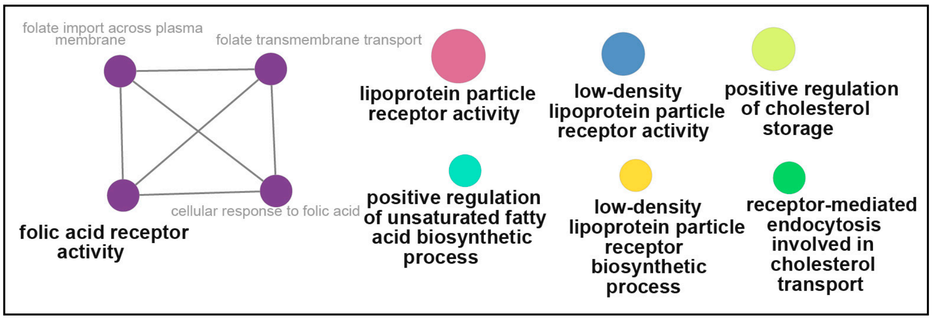
folate import across plasma membrane
folate transmembrane transport
cellular response to folic acid
folic acid receptor activity
lipoprotein particle receptor activity
low-density lipoprotein particle receptor activity
positive regulation of cholesterol storage
positive regulation of unsaturated fatty acid biosynthetic process
low-density lipoprotein particle receptor biosynthetic process
receptor-mediated endocytosis involved in cholesterol transport

(D)

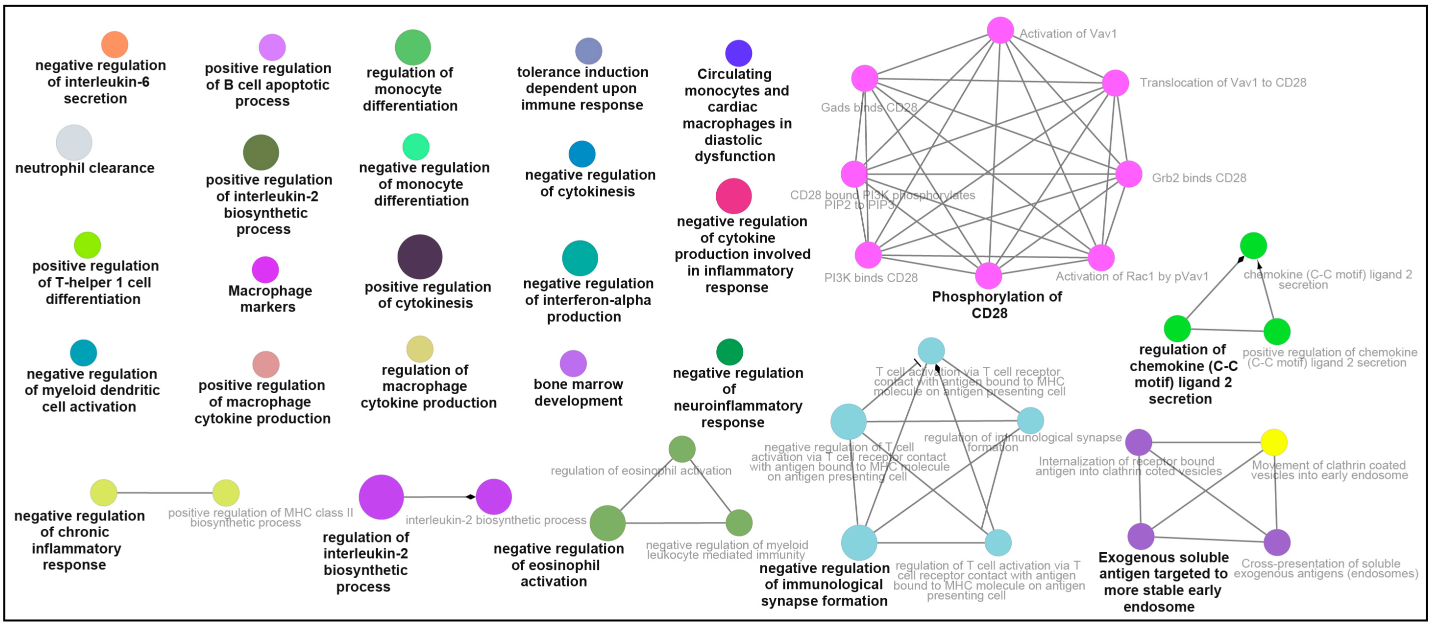

negative regulation of interleukin-6 secretion
positive regulation of B cell apoptotic process
regulation of monocyte differentiation
tolerance induction dependent upon immune response
Circulating monocytes and cardiac macrophages in diastolic dysfunction
Activation of Vav1
Translocation of Vav1 to CD28
Gads binds CD28
Grb2 binds CD28
CD28 bound PI3K phosphorylates PIP2 to PIP3
PI3K binds CD28
Activation of Rac1 by pVav1
Phosphorylation of CD28
neutrophil clearance
positive regulation of interleukin-2 biosynthetic process
negative regulation of monocyte differentiation
negative regulation of cytokinesis
negative regulation of cytokine production involved in inflammatory response
positive regulation of T-helper 1 cell differentiation
Macrophage markers
positive regulation of cytokinesis
negative regulation of interferon-alpha production
chemokine (C-C motif) ligand 2 secretion
regulation of chemokine (C-C motif) ligand 2 secretion
positive regulation of chemokine (C-C motif) ligand 2 secretion
negative regulation of myeloid dendritic cell activation
positive regulation of macrophage cytokine production
regulation of macrophage cytokine production
bone marrow development
negative regulation of neuroinflammatory response
T cell activation via T cell receptor contact with antigen bound to MHC molecule on antigen presenting cell
negative regulation of T cell activation via T cell receptor contact with antigen bound to MHC molecule on antigen presenting cell
regulation of immunological synapse formation
Internalization of receptor bound antigen into clathrin coted vesicles
Movement of clathrin coated vesicles into early endosome
regulation of eosinophil activation
negative regulation of chronic inflammatory response
positive regulation of MHC class II biosynthetic process
regulation of interleukin-2 biosynthetic process
interleukin-2 biosynthetic process
negative regulation of eosinophil activation
negative regulation of myeloid leukocyte mediated immunity
negative regulation of immunological synapse formation
regulation of T cell activation via T cell receptor contact with antigen bound to MHC molecule on antigen presenting cell
Exogenous soluble antigen targeted to more stable early endosome
Cross-presentation of soluble exogenous antigens (endosomes)

(E)

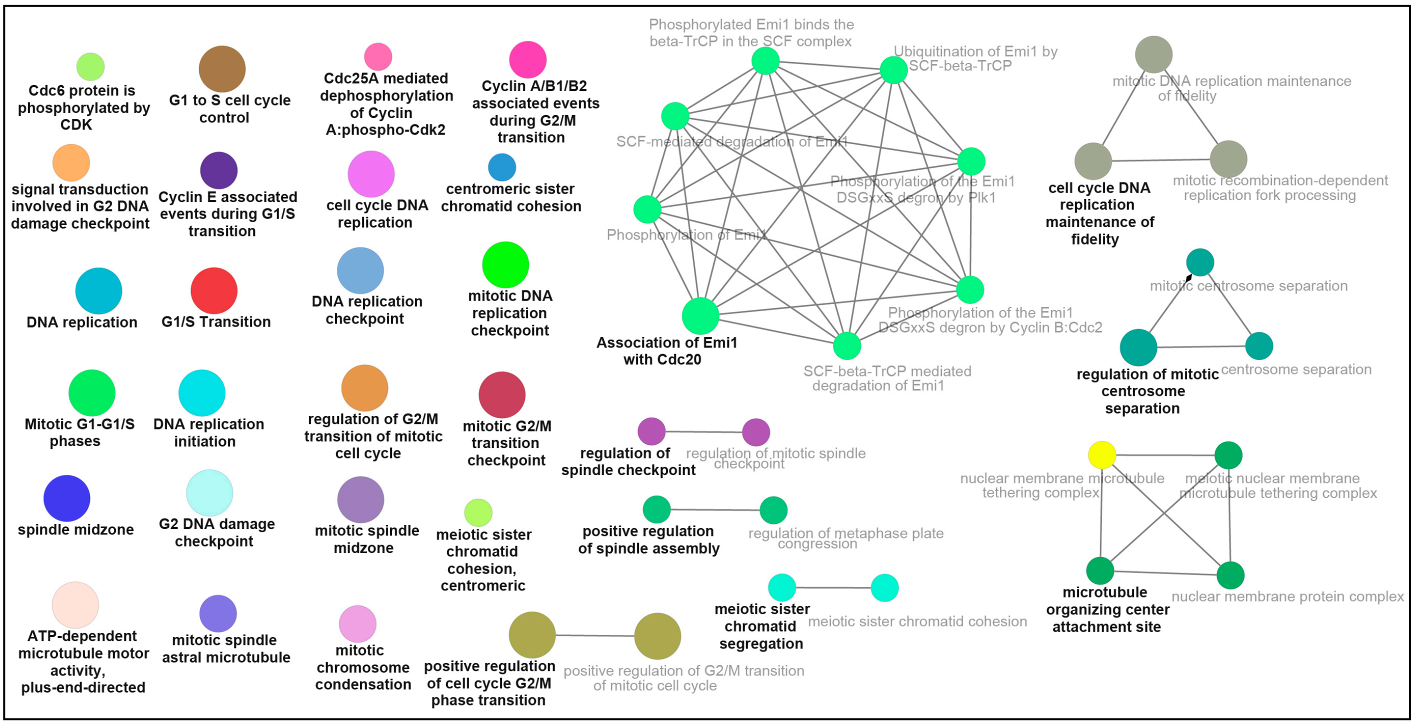

Cdc6 protein is phosphorylated by CDK
G1 to S cell cycle control
Cdc25A mediated dephosphorylation of Cyclin A:phospho-Cdk2
Cyclin A/B1/B2 associated events during G2/M transition
Phosphorylated Emi1 binds the beta-TrCP in the SCF complex
Ubiquitination of Emi1 by SCF-beta-TrCP
mitotic DNA replication maintenance of fidelity
signal transduction involved in G2 DNA damage checkpoint
Cyclin E associated events during G1/S transition
cell cycle DNA replication
centromeric sister chromatid cohesion
SCF-mediated degradation of Emi1
Phosphorylation of the Emi1 DSGxxS degron by Plk1
Phosphorylation of Emi1
cell cycle DNA replication maintenance of fidelity
mitotic recombination-dependent replication fork processing
DNA replication
G1/S Transition
DNA replication checkpoint
mitotic DNA replication checkpoint
mitotic centrosome separation
Association of Emi1 with Cdc20
Phosphorylation of the Emi1 DSGxxS degron by Cyclin B:Cdc2
SCF-beta-TrCP mediated degradation of Emi1
regulation of mitotic centrosome separation
centrosome separation
Mitotic G1-G1/S phases
DNA replication initiation
regulation of G2/M transition of mitotic cell cycle
mitotic G2/M transition checkpoint
regulation of spindle checkpoint
regulation of mitotic spindle checkpoint
nuclear membrane microtubule tethering complex
meiotic nuclear membrane microtubule tethering complex
spindle midzone
G2 DNA damage checkpoint
mitotic spindle midzone
meiotic sister chromatid cohesion, centromeric
positive regulation of spindle assembly
regulation of metaphase plate congression
microtubule organizing center attachment site
nuclear membrane protein complex
meiotic sister chromatid segregation
meiotic sister chromatid cohesion
ATP-dependent microtubule motor activity, plus-end-directed
mitotic spindle astral microtubule
mitotic chromosome condensation
positive regulation of cell cycle G2/M phase transition
positive regulation of G2/M transition of mitotic cell cycle

(F)

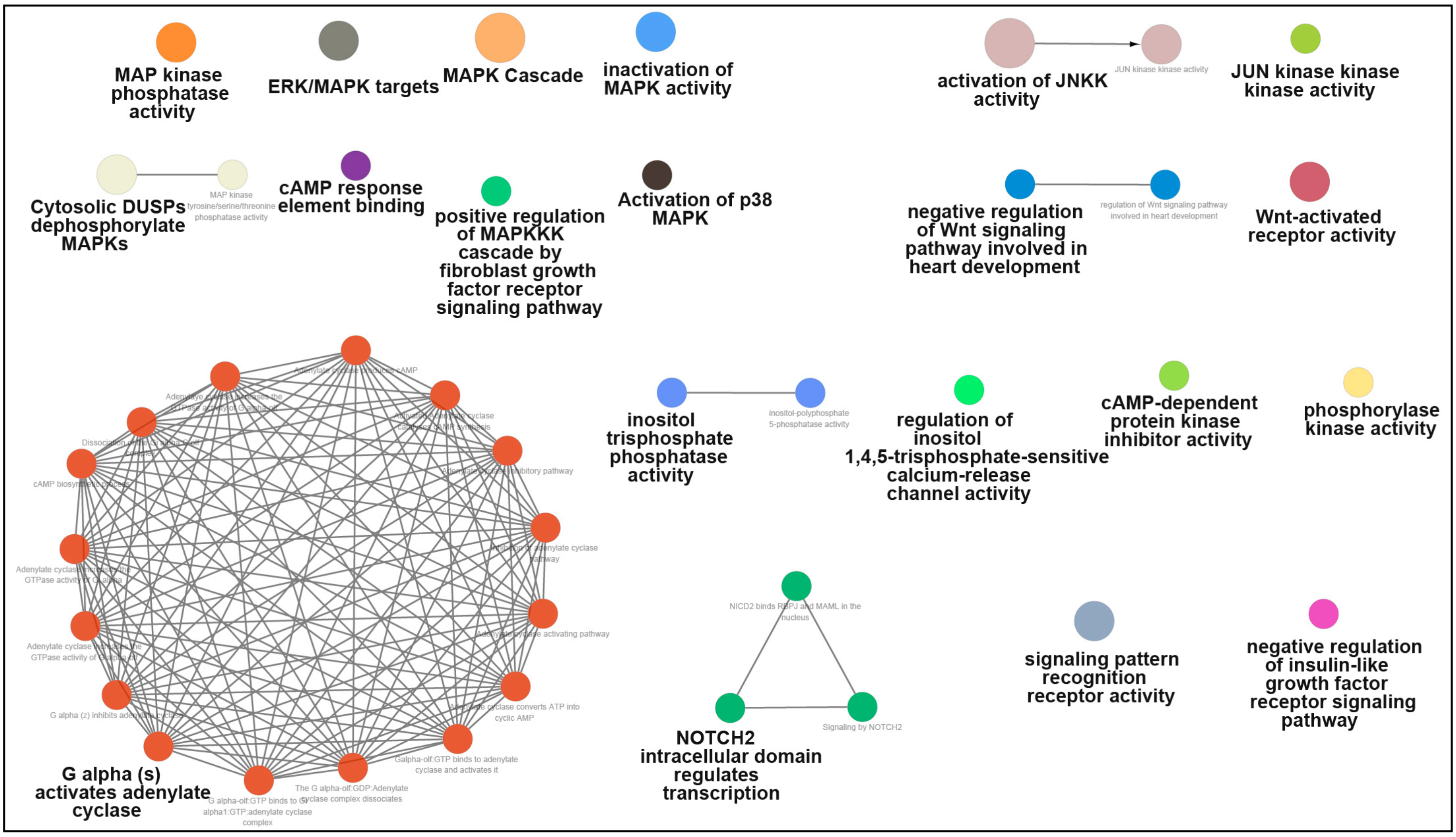

(G)

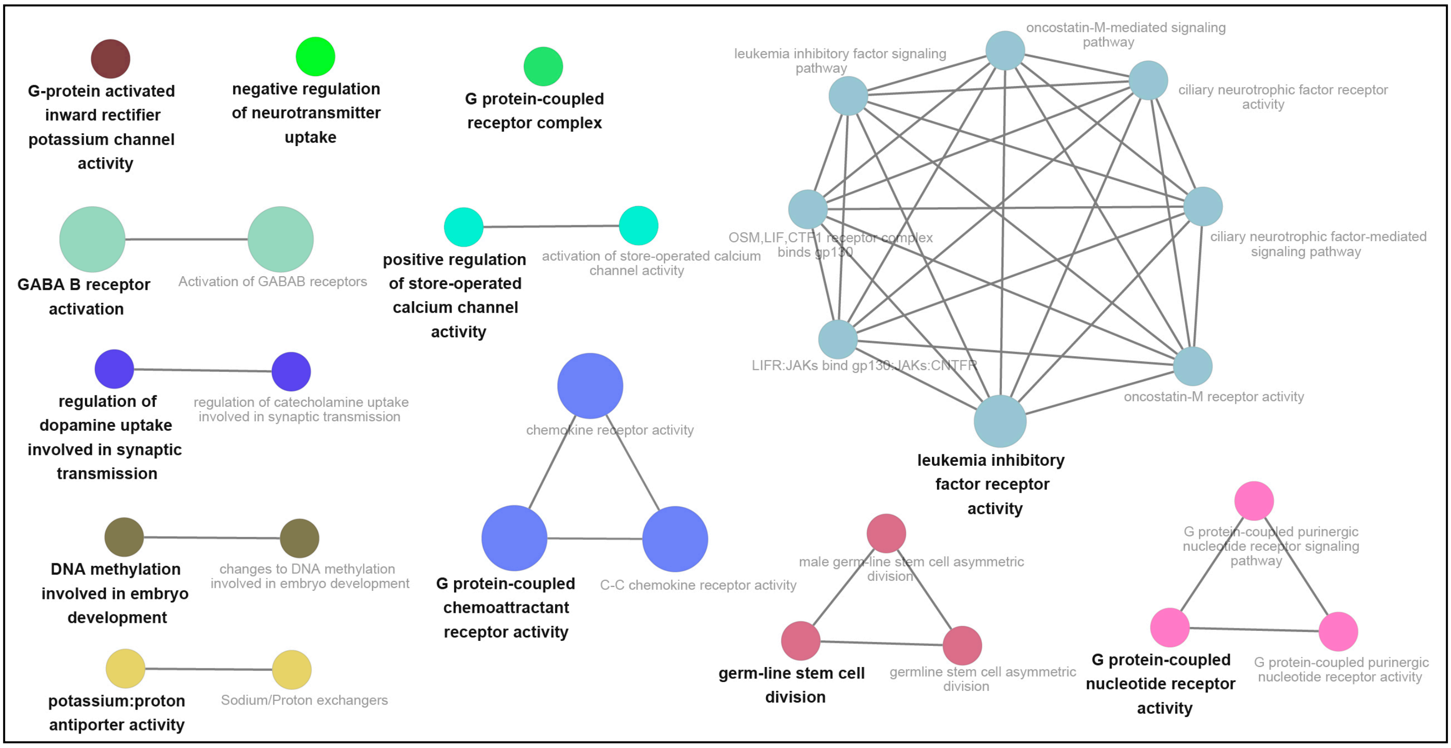

(H) GPCR and cell channel

Figure S2. Functional enrichment analysis of down-regulated DEGs from ClueGO. (A) prolactin, opioid signaling pathway and osteoclast fusion; (B) toll-like receptor signaling pathway; (C) cell migration; (D) fatty acid metabolism; (E) inflammation and cytokine; (F) cell checkpoints, cell cycle and cell structure; (G) signaling pathways; (H) GPCR and cell channel activity.
