# Supplementary material for: Transcriptomic Changes in Mouse Bone Marrow-Derived Macrophages Exposed to Neuropeptide FF
Source: Genes (Basel). 2021 May 9;12(5):705. doi: 10.3390/genes12050705 (PMC8151073; doi:10.3390/genes12050705)
Supplement: Supplementary file 1 [file genes-12-00705-s001.zip › genes-1147651-supplementary/Figure S2 DEG PPI cluego-new-down-ok.pdf]

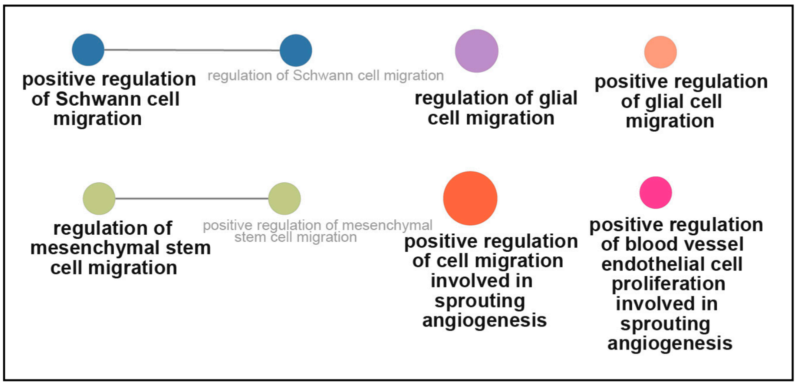
positive regulation of Schwann cell migration
regulation of Schwann cell migration
regulation of glial cell migration
positive regulation of glial cell migration
regulation of mesenchymal stem cell migration
positive regulation of mesenchymal stem cell migration
positive regulation of cell migration involved in sprouting angiogenesis
positive regulation of blood vessel endothelial cell proliferation involved in sprouting angiogenesis

(A)

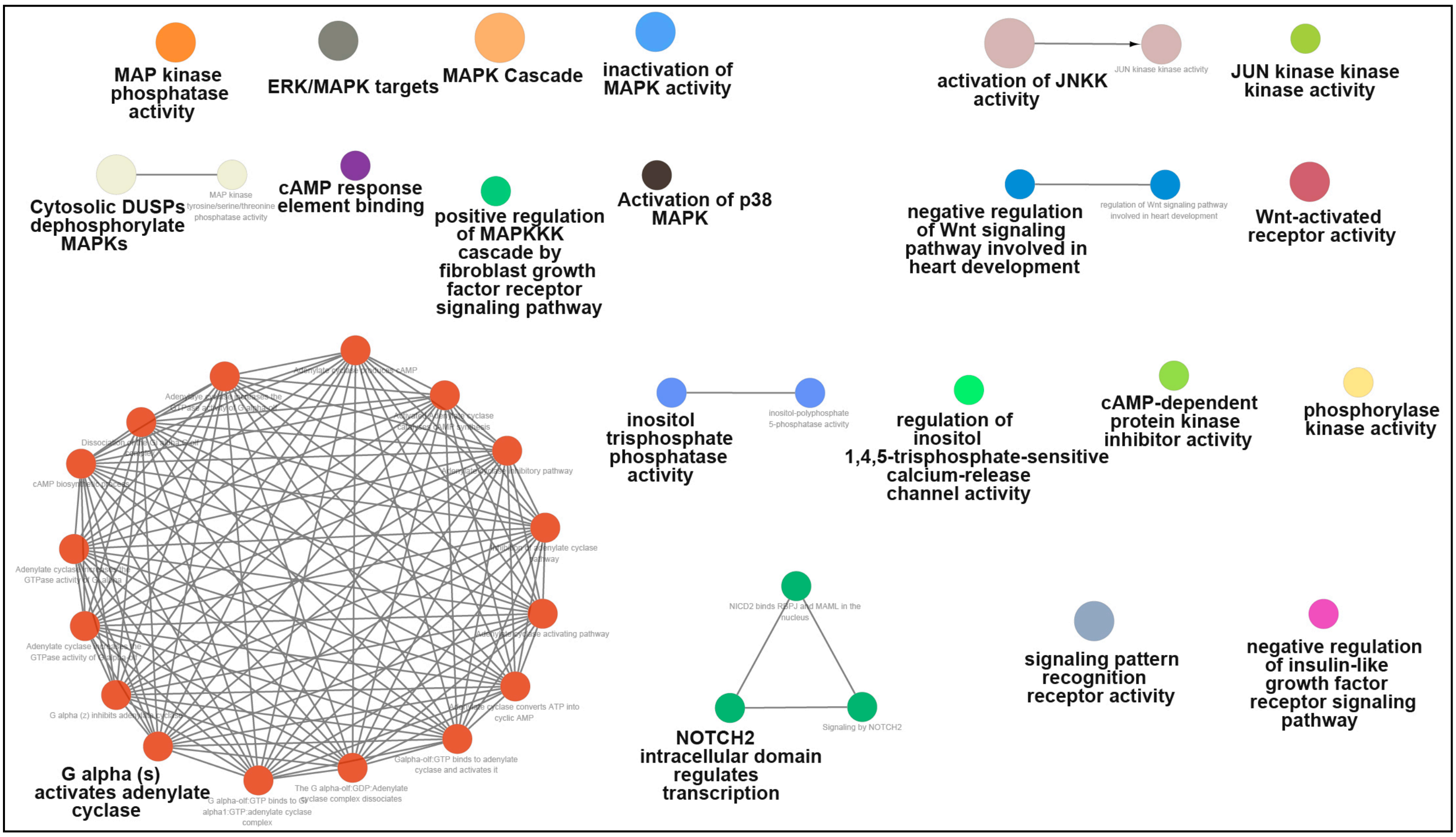
MAP kinase phosphatase activity
ERK/MAPK targets
MAPK Cascade
inactivation of MAPK activity
activation of JNKK activity
JUN kinase kinase activity
JUN kinase kinase kinase activity
Cytosolic DUSPs dephosphorylate MAPKs
MAP kinase tyrosine/serine/threonine phosphatase activity
cAMP response element binding
positive regulation of MAPKKK cascade by fibroblast growth factor receptor signaling pathway
Activation of p38 MAPK
negative regulation of Wnt signaling pathway involved in heart development
regulation of Wnt signaling pathway involved in heart development
Wnt-activated receptor activity
inositol trisphosphate phosphatase activity
inositol-polyphosphate 5-phosphatase activity
regulation of inositol 1,4,5-trisphosphate-sensitive calcium-release channel activity
cAMP-dependent protein kinase inhibitor activity
phosphorylase kinase activity
G alpha (s) activates adenylate cyclase
Signaling by NOTCH2
NOTCH2 intracellular domain regulates transcription
signaling pattern recognition receptor activity
negative regulation of insulin-like growth factor receptor signaling pathway

(B)

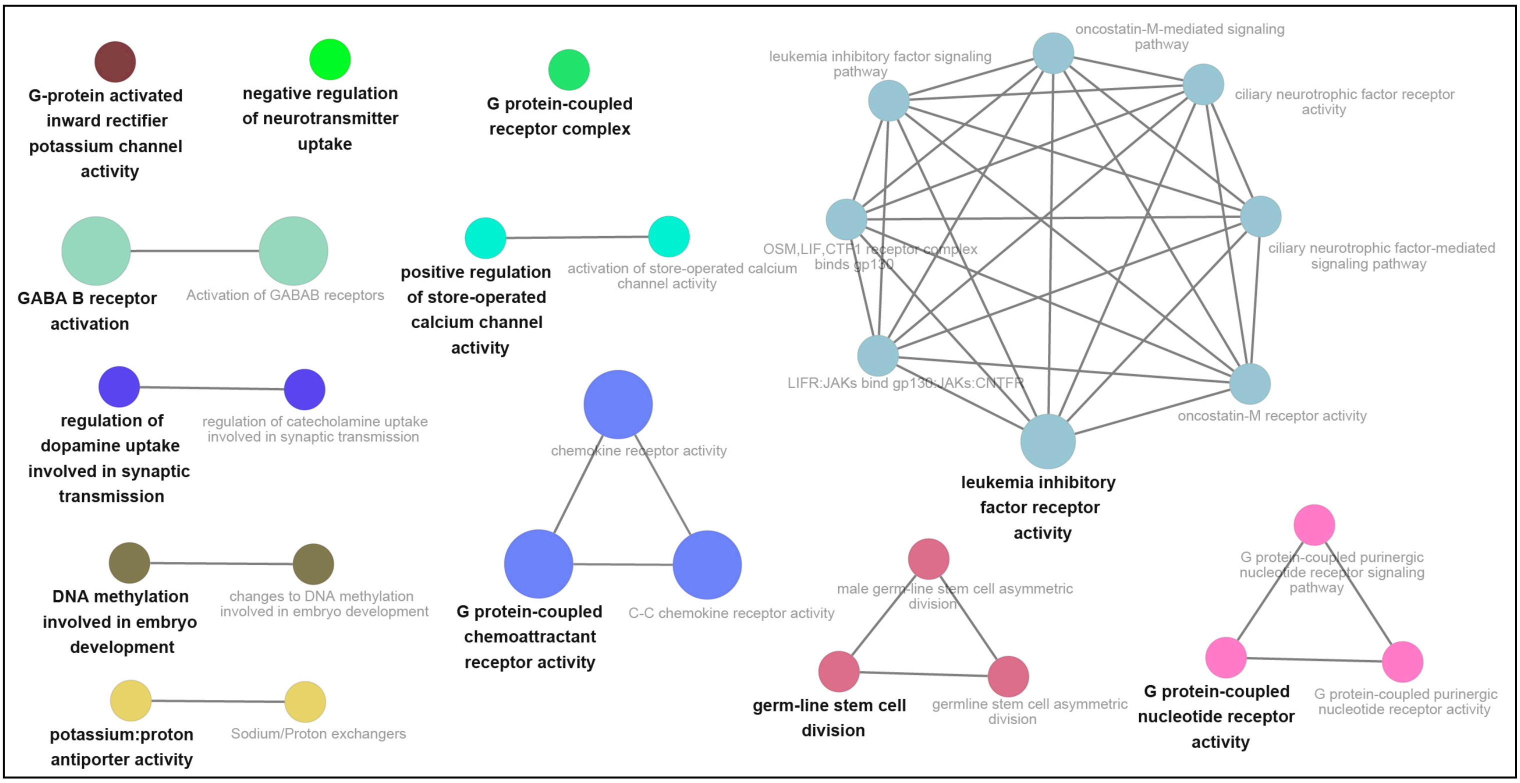

(C) GPCR and cell channel

Figure S2. Functional enrichment analysis of down-regulated DEGs from ClueGO. (A) cell migration; (B) signaling pathways, and (C) GPCR and cell channel activity.
