# Supplementary material for: Transcriptomic Changes in Mouse Bone Marrow-Derived Macrophages Exposed to Neuropeptide FF
Source: Genes (Basel). 2021 May 9;12(5):705. doi: 10.3390/genes12050705 (PMC8151073; doi:10.3390/genes12050705)
Supplement: Supplementary file 1 [file genes-12-00705-s001.zip › genes-1147651-supplementary/Figure S3 DEG PPI cluego-new-up-all.pdf]

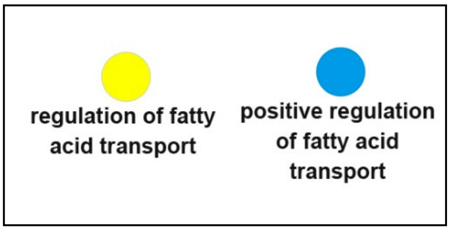
regulation of fatty acid transport
positive regulation of fatty acid transport

(A)

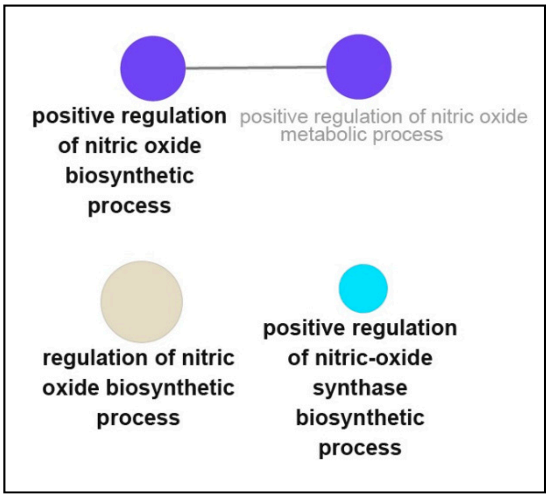
positive regulation of nitric oxide biosynthetic process
positive regulation of nitric oxide metabolic process
regulation of nitric oxide biosynthetic process
positive regulation of nitric-oxide synthase biosynthetic process

(B)

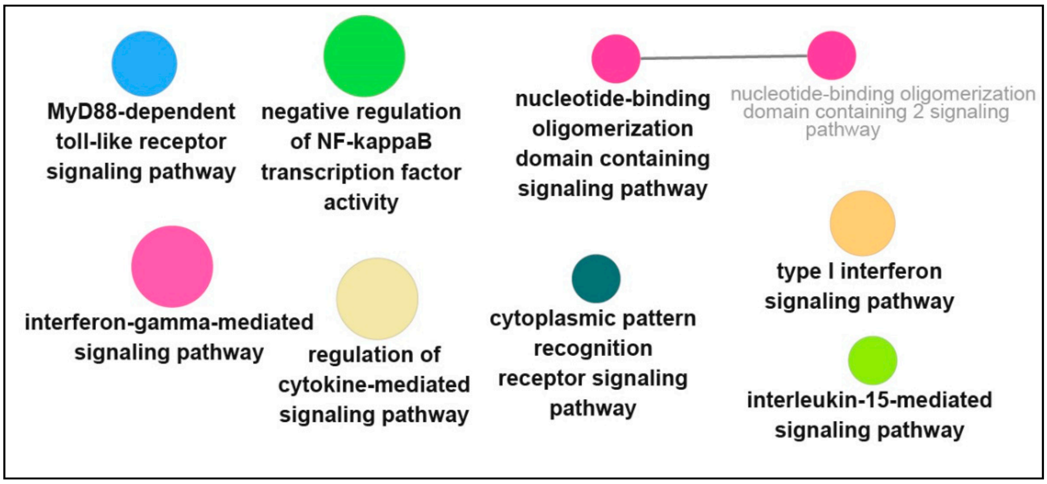
MyD88-dependent toll-like receptor signaling pathway
negative regulation of NF-kappaB transcription factor activity
nucleotide-binding oligomerization domain containing signaling pathway
nucleotide-binding oligomerization domain containing 2 signaling pathway
interferon-gamma-mediated signaling pathway
regulation of cytokine-mediated signaling pathway
cytoplasmic pattern recognition receptor signaling pathway
type I interferon signaling pathway
interleukin-15-mediated signaling pathway

(C)

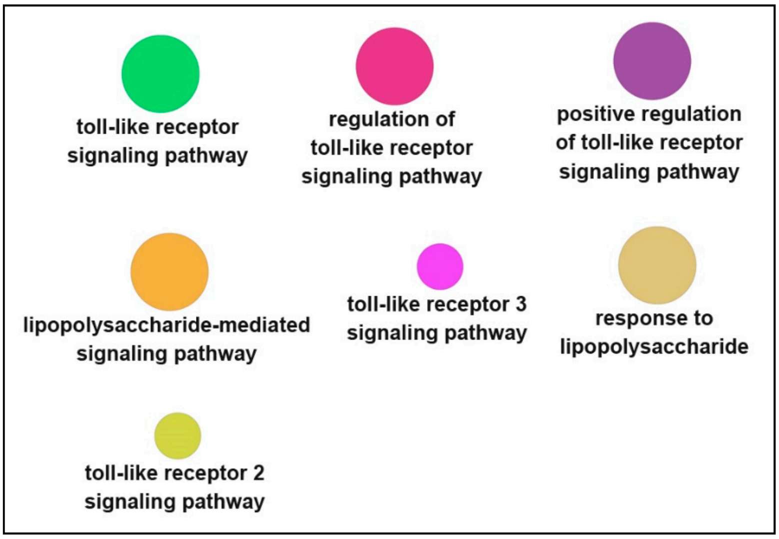

toll-like receptor signaling pathway
regulation of toll-like receptor signaling pathway
positive regulation of toll-like receptor signaling pathway
lipopolysaccharide-mediated signaling pathway
toll-like receptor 3 signaling pathway
response to lipopolysaccharide
toll-like receptor 2 signaling pathway

(D)

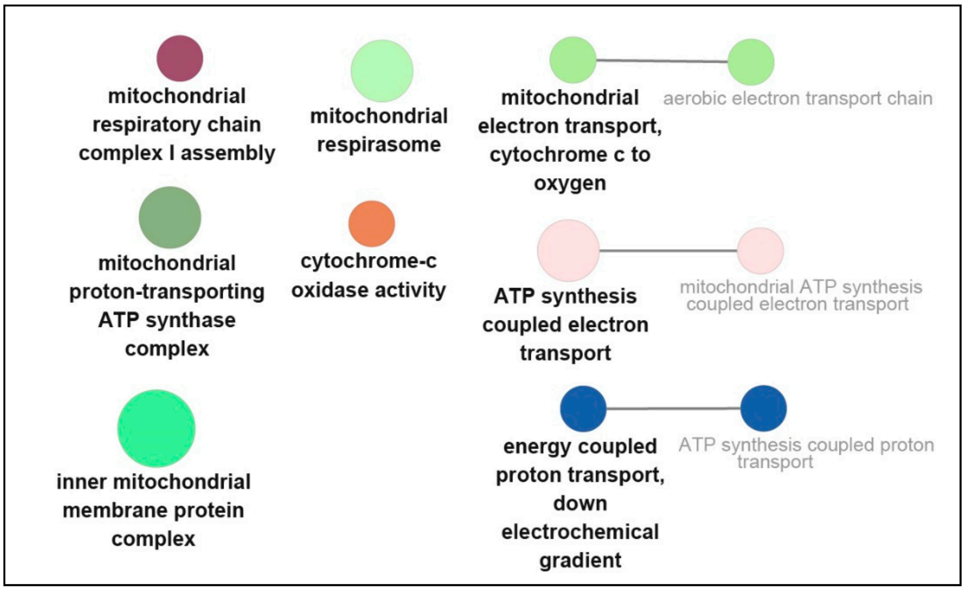

mitochondrial respiratory chain complex I assembly
mitochondrial respirasome
mitochondrial electron transport, cytochrome c to oxygen
aerobic electron transport chain
mitochondrial proton-transporting ATP synthase complex
cytochrome-c oxidase activity
ATP synthesis coupled electron transport
mitochondrial ATP synthesis coupled electron transport
inner mitochondrial membrane protein complex
energy coupled proton transport, down electrochemical gradient
ATP synthesis coupled proton transport

(E)

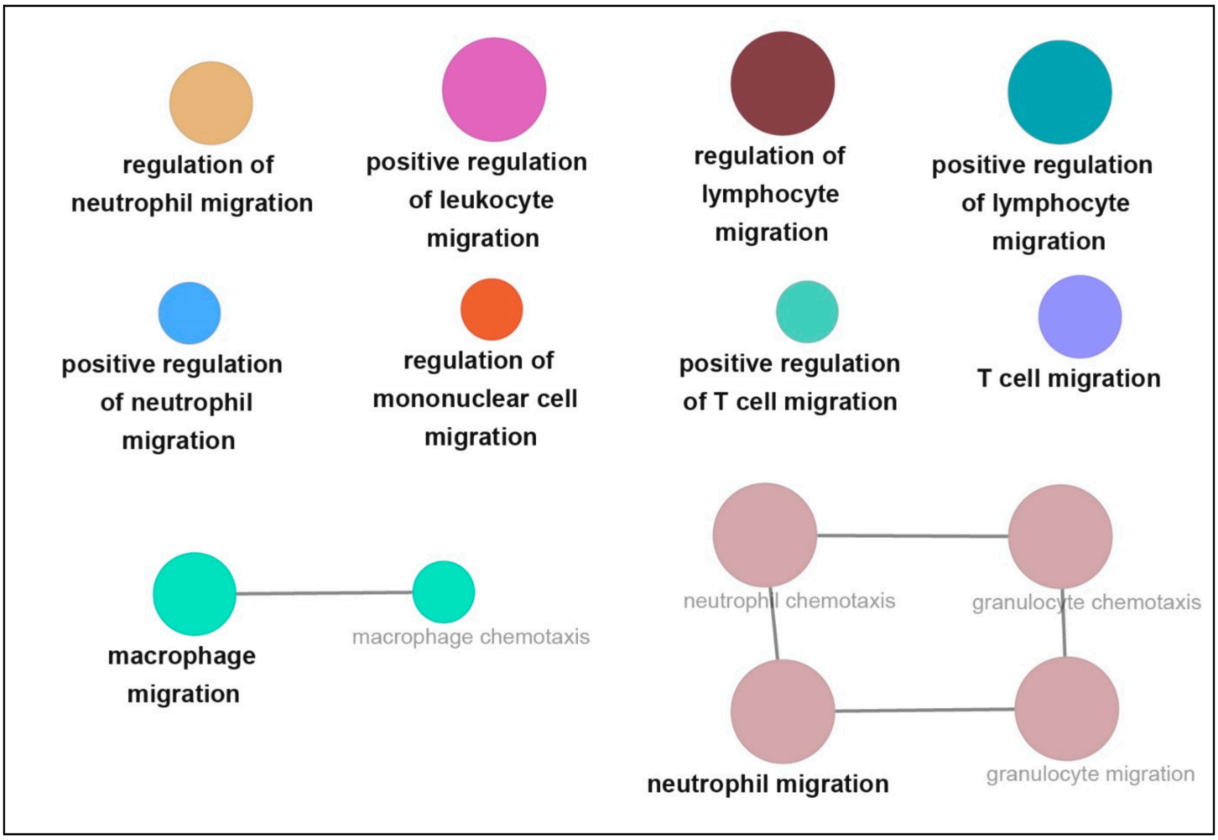

regulation of neutrophil migration
positive regulation of leukocyte migration
regulation of lymphocyte migration
positive regulation of lymphocyte migration
positive regulation of neutrophil migration
regulation of mononuclear cell migration
positive regulation of T cell migration
T cell migration
macrophage migration
macrophage chemotaxis
neutrophil chemotaxis
granulocyte chemotaxis
neutrophil migration
granulocyte migration

(F)

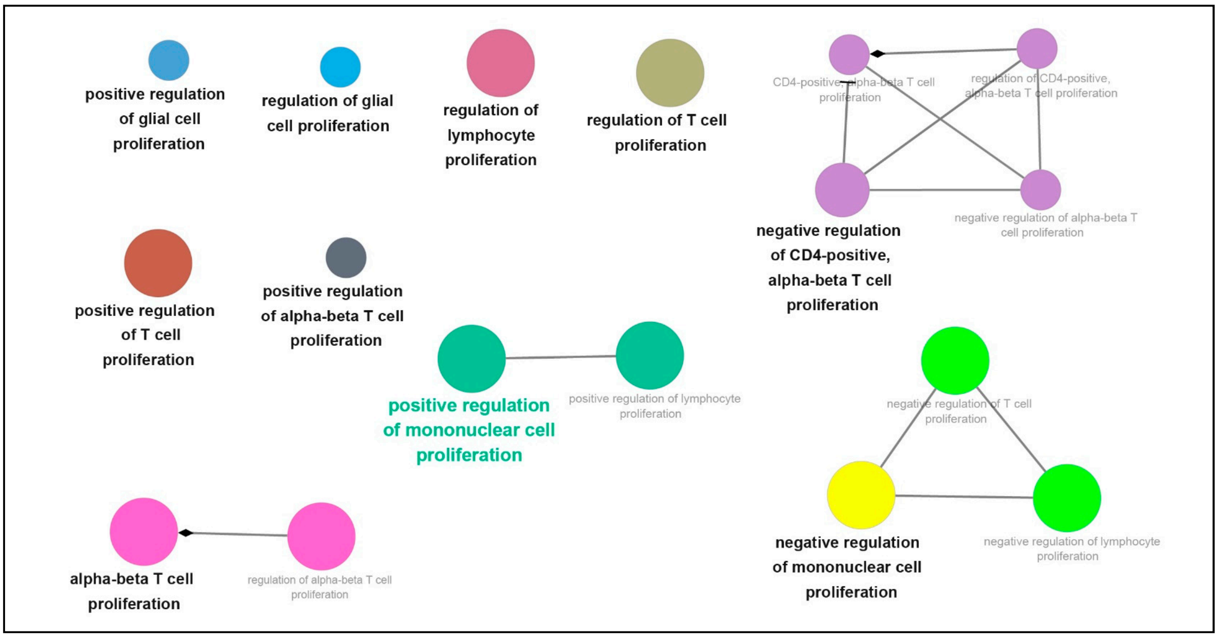

positive regulation of glial cell proliferation
regulation of glial cell proliferation
regulation of lymphocyte proliferation
regulation of T cell proliferation
CD4-positive, alpha-beta T cell proliferation
regulation of CD4-positive, alpha-beta T cell proliferation
negative regulation of CD4-positive, alpha-beta T cell proliferation
negative regulation of alpha-beta T cell proliferation
positive regulation of T cell proliferation
positive regulation of alpha-beta T cell proliferation
positive regulation of mononuclear cell proliferation
positive regulation of lymphocyte proliferation
negative regulation of T cell proliferation
negative regulation of mononuclear cell proliferation
negative regulation of lymphocyte proliferation
alpha-beta T cell proliferation
regulation of alpha-beta T cell proliferation

(G)

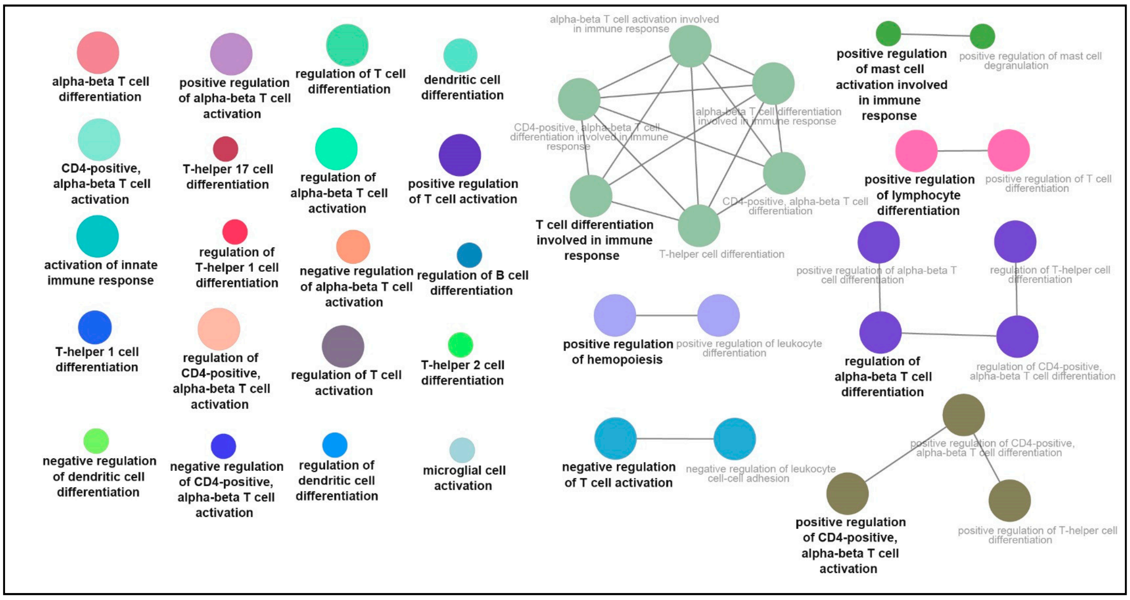
alpha-beta T cell differentiation
positive regulation of alpha-beta T cell activation
regulation of T cell differentiation
dendritic cell differentiation
alpha-beta T cell activation involved in immune response
positive regulation of mast cell activation involved in immune response
positive regulation of mast cell degranulation
CD4-positive, alpha-beta T cell activation
T-helper 17 cell differentiation
regulation of alpha-beta T cell activation
positive regulation of T cell activation
CD4-positive, alpha-beta T cell differentiation involved in immune response
alpha-beta T cell differentiation involved in immune response
positive regulation of lymphocyte differentiation
positive regulation of T cell differentiation
activation of innate immune response
regulation of T-helper 1 cell differentiation
negative regulation of alpha-beta T cell activation
regulation of B cell differentiation
T cell differentiation involved in immune response
T-helper cell differentiation
CD4-positive, alpha-beta T cell differentiation
positive regulation of alpha-beta T cell differentiation
regulation of T-helper cell differentiation
T-helper 1 cell differentiation
regulation of CD4-positive, alpha-beta T cell activation
regulation of T cell activation
T-helper 2 cell differentiation
positive regulation of hemopoiesis
positive regulation of leukocyte differentiation
regulation of alpha-beta T cell differentiation
regulation of CD4-positive, alpha-beta T cell differentiation
positive regulation of CD4-positive, alpha-beta T cell differentiation
negative regulation of dendritic cell differentiation
negative regulation of CD4-positive, alpha-beta T cell activation
regulation of dendritic cell differentiation
microglial cell activation
negative regulation of T cell activation
negative regulation of leukocyte cell-cell adhesion
positive regulation of CD4-positive, alpha-beta T cell activation
positive regulation of T-helper cell differentiation

(H)

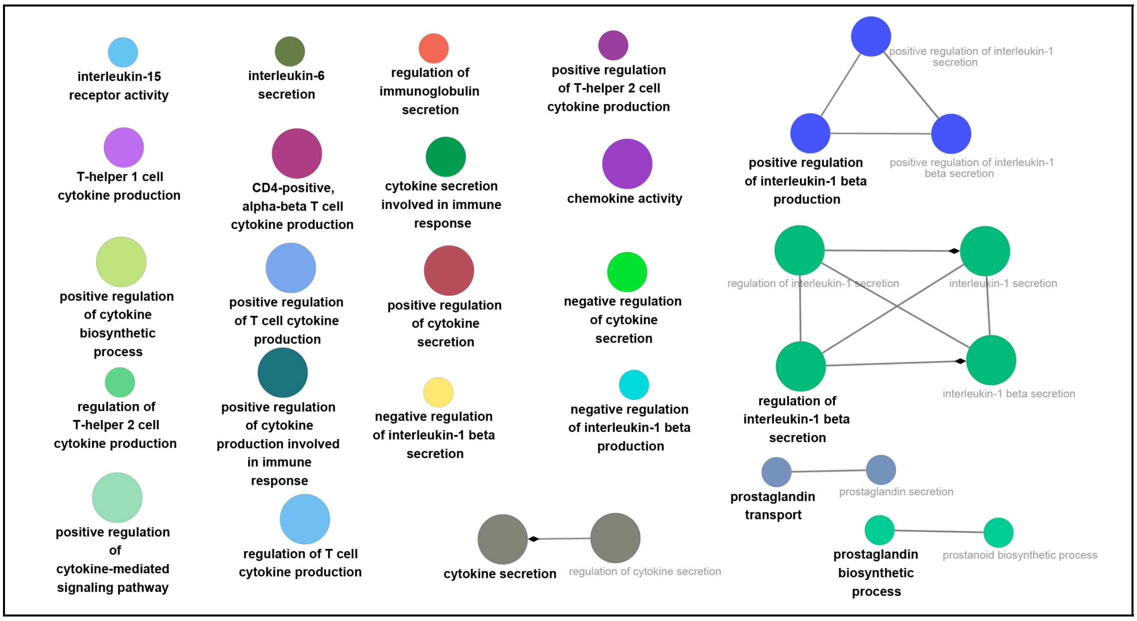
interleukin-15 receptor activity
interleukin-6 secretion
regulation of immunoglobulin secretion
positive regulation of T-helper 2 cell cytokine production
positive regulation of interleukin-1 secretion
T-helper 1 cell cytokine production
CD4-positive, alpha-beta T cell cytokine production
cytokine secretion involved in immune response
chemokine activity
positive regulation of interleukin-1 beta production
positive regulation of interleukin-1 beta secretion
regulation of interleukin-1 secretion
interleukin-1 secretion
positive regulation of cytokine biosynthetic process
positive regulation of T cell cytokine production
positive regulation of cytokine secretion
negative regulation of cytokine secretion
regulation of T-helper 2 cell cytokine production
positive regulation of cytokine production involved in immune response
negative regulation of interleukin-1 beta secretion
negative regulation of interleukin-1 beta production
regulation of interleukin-1 beta secretion
interleukin-1 beta secretion
prostaglandin transport
prostaglandin secretion
positive regulation of cytokine-mediated signaling pathway
regulation of T cell cytokine production
cytokine secretion
regulation of cytokine secretion
prostaglandin biosynthetic process
prostanoid biosynthetic process

(I)

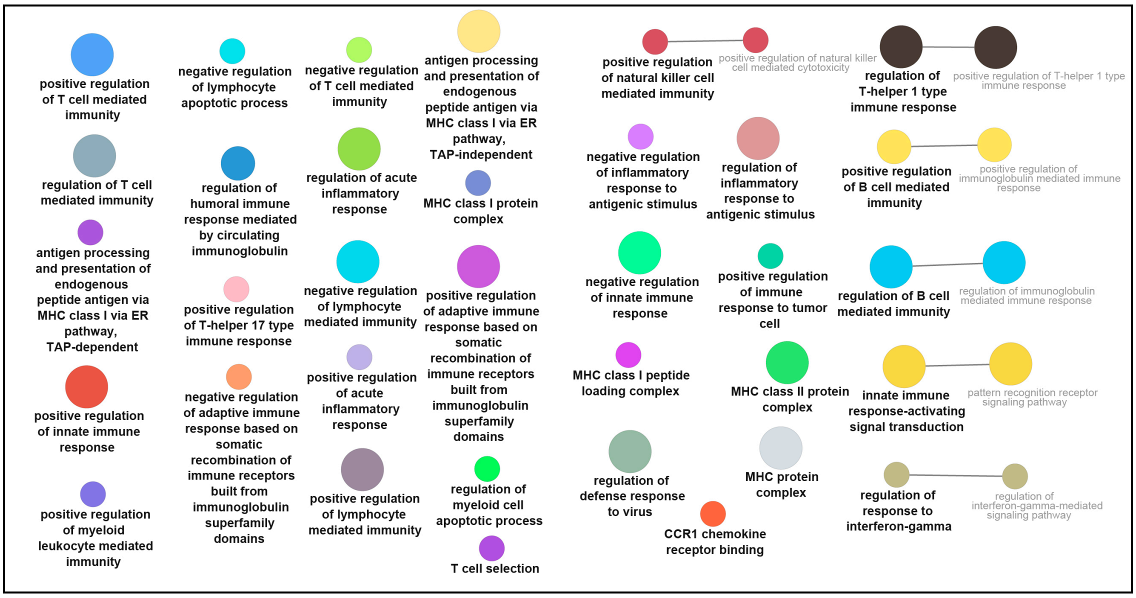
positive regulation of T cell mediated immunity
regulation of T cell mediated immunity
antigen processing and presentation of endogenous peptide antigen via MHC class I via ER pathway, TAP-dependent
positive regulation of innate immune response
positive regulation of myeloid leukocyte mediated immunity
negative regulation of lymphocyte apoptotic process
regulation of humoral immune response mediated by circulating immunoglobulin
positive regulation of T-helper 17 type immune response
negative regulation of adaptive immune response based on somatic recombination of immune receptors built from immunoglobulin superfamily domains
negative regulation of T cell mediated immunity
regulation of acute inflammatory response
negative regulation of lymphocyte mediated immunity
positive regulation of acute inflammatory response
positive regulation of lymphocyte mediated immunity
antigen processing and presentation of endogenous peptide antigen via MHC class I via ER pathway, TAP-independent
MHC class I protein complex
positive regulation of adaptive immune response based on somatic recombination of immune receptors built from immunoglobulin superfamily domains
regulation of myeloid cell apoptotic process
T cell selection
positive regulation of natural killer cell mediated immunity
positive regulation of natural killer cell mediated cytotoxicity
negative regulation of inflammatory response to antigenic stimulus
regulation of inflammatory response to antigenic stimulus
negative regulation of innate immune response
positive regulation of immune response to tumor cell
MHC class I peptide loading complex
MHC class II protein complex
regulation of defense response to virus
MHC protein complex
CCR1 chemokine receptor binding
regulation of T-helper 1 type immune response
positive regulation of T-helper 1 type immune response
positive regulation of B cell mediated immunity
positive regulation of immunoglobulin mediated immune response
regulation of B cell mediated immunity
regulation of immunoglobulin mediated immune response
innate immune response-activating signal transduction
pattern recognition receptor signaling pathway
regulation of response to interferon-gamma
regulation of interferon-gamma-mediated signaling pathway

(J)

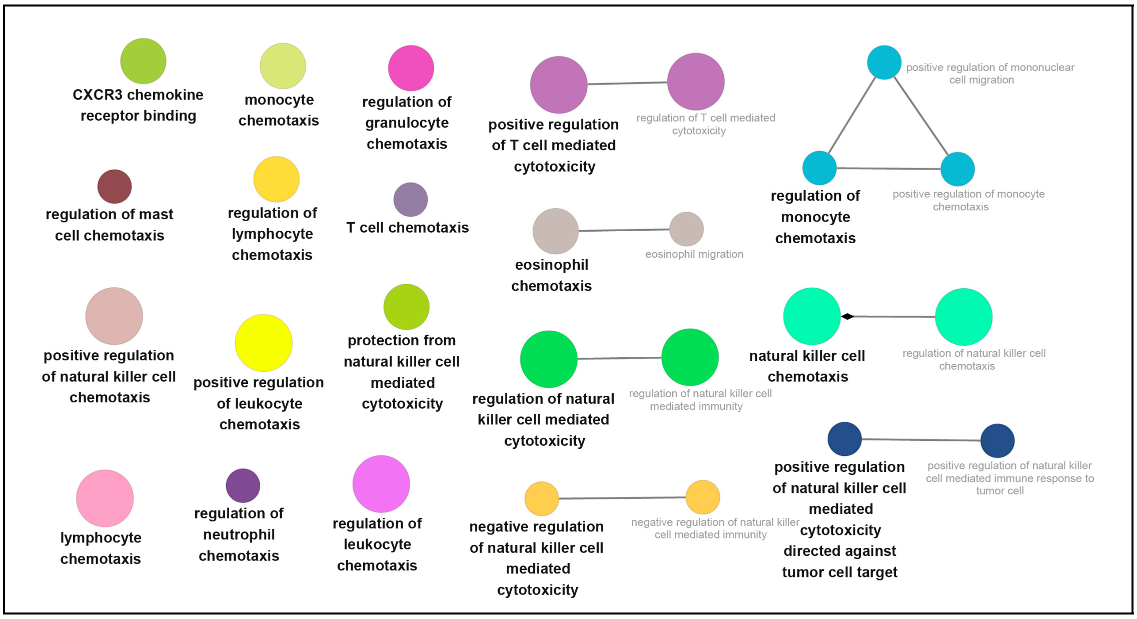
CXCR3 chemokine receptor binding
monocyte chemotaxis
regulation of granulocyte chemotaxis
regulation of mast cell chemotaxis
regulation of lymphocyte chemotaxis
T cell chemotaxis
positive regulation of natural killer cell chemotaxis
positive regulation of leukocyte chemotaxis
protection from natural killer cell mediated cytotoxicity
lymphocyte chemotaxis
regulation of neutrophil chemotaxis
regulation of leukocyte chemotaxis
positive regulation of T cell mediated cytotoxicity
regulation of T cell mediated cytotoxicity
eosinophil chemotaxis
eosinophil migration
regulation of natural killer cell mediated cytotoxicity
regulation of natural killer cell mediated immunity
negative regulation of natural killer cell mediated cytotoxicity
negative regulation of natural killer cell mediated immunity
positive regulation of mononuclear cell migration
regulation of monocyte chemotaxis
positive regulation of monocyte chemotaxis
natural killer cell chemotaxis
regulation of natural killer cell chemotaxis
positive regulation of natural killer cell mediated cytotoxicity directed against tumor cell target
positive regulation of natural killer cell mediated immune response to tumor cell

(K)

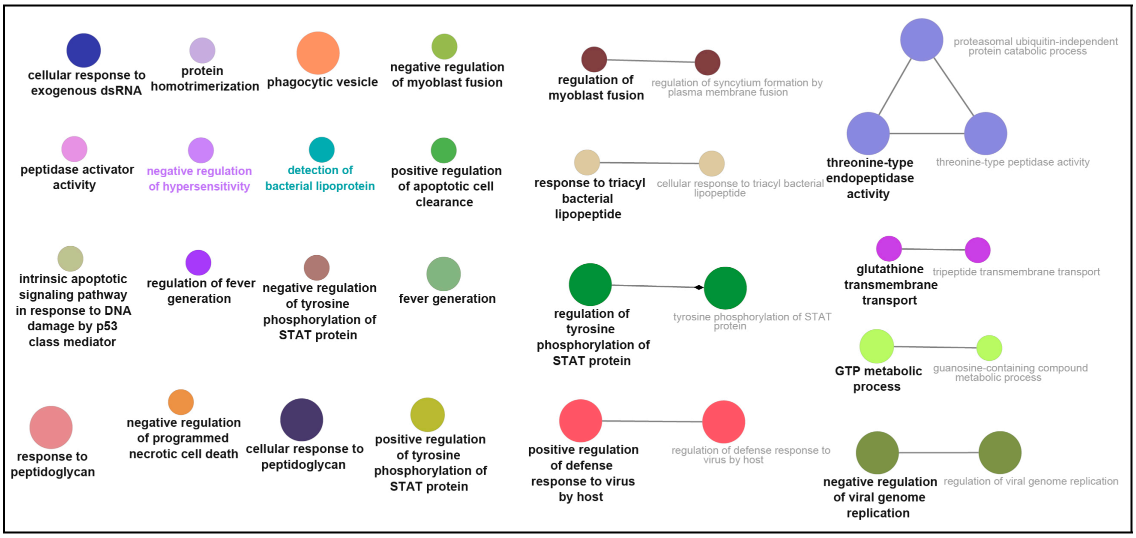

(L)

Figure S3. Functional enrichment analysis of up-regulated DEGs from ClueGO. (A) fatty acid metabolism; (B) nitric oxide production; (C) immune signaling pathways; (D) toll-like signaling pathways; (E) mitochondrial function; (F) proliferation of immune cells; (G) cell proliferation; (H) activation and differentiation of immune cells; (I) cytokine production or activity; (J) inflammation and immune activity; (K) chemotaxis and cototoxicity of immune cells; (L) other biological activities.
