# Supplementary material for: Transcriptomic Changes in Mouse Bone Marrow-Derived Macrophages Exposed to Neuropeptide FF
Source: Genes (Basel). 2021 May 9;12(5):705. doi: 10.3390/genes12050705 (PMC8151073; doi:10.3390/genes12050705)
Supplement: Supplementary file 1 [file genes-12-00705-s001.zip › genes-1147651-supplementary/Figure S3 DEG PPI cluego-new-up-ok.pdf]

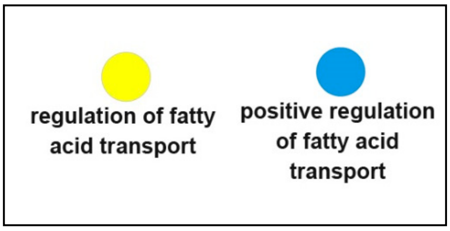
regulation of fatty acid transport
positive regulation of fatty acid transport

(A)

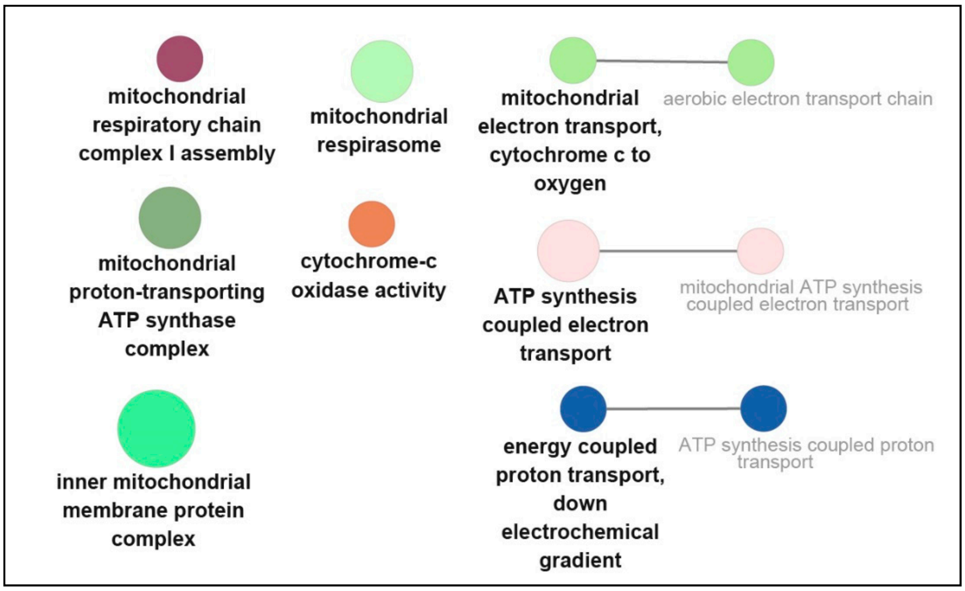
mitochondrial respiratory chain complex I assembly
mitochondrial respirasome
mitochondrial electron transport, cytochrome c to oxygen
aerobic electron transport chain
mitochondrial proton-transporting ATP synthase complex
cytochrome-c oxidase activity
ATP synthesis coupled electron transport
mitochondrial ATP synthesis coupled electron transport
inner mitochondrial membrane protein complex
energy coupled proton transport, down electrochemical gradient
ATP synthesis coupled proton transport

(B)

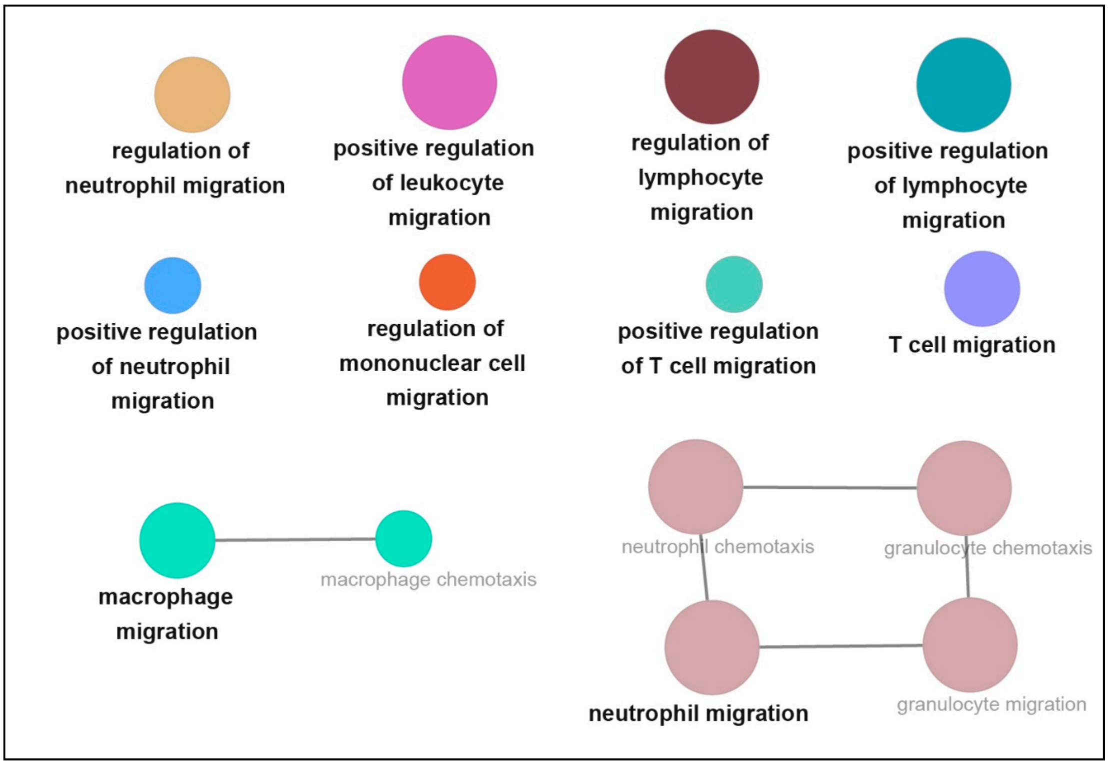
regulation of neutrophil migration
positive regulation of leukocyte migration
regulation of lymphocyte migration
positive regulation of lymphocyte migration
positive regulation of neutrophil migration
regulation of mononuclear cell migration
positive regulation of T cell migration
T cell migration
macrophage migration
macrophage chemotaxis
neutrophil chemotaxis
granulocyte chemotaxis
neutrophil migration
granulocyte migration

(C)

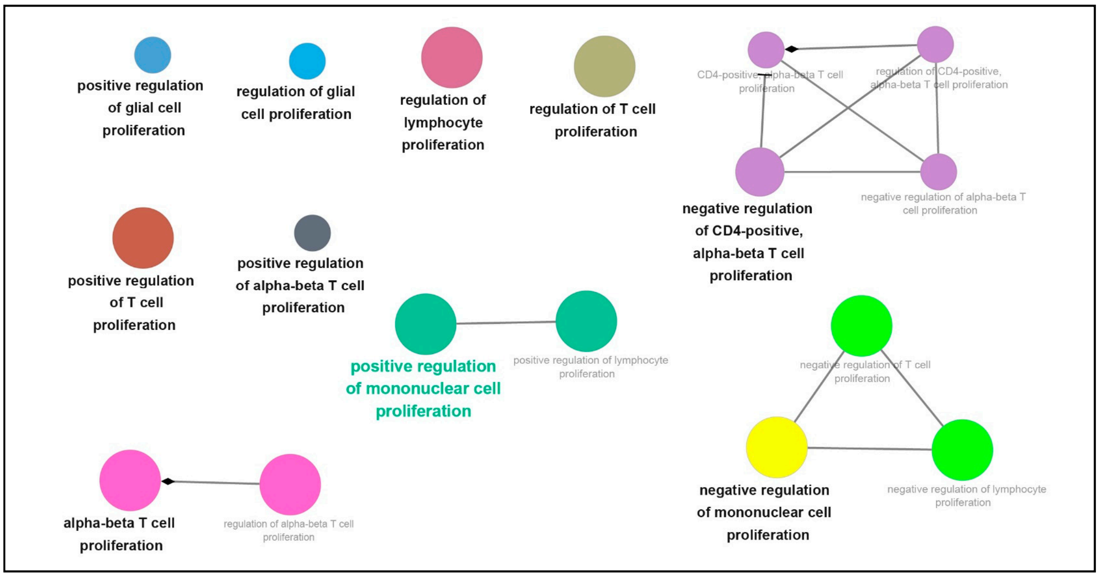
positive regulation of glial cell proliferation
regulation of glial cell proliferation
regulation of lymphocyte proliferation
regulation of T cell proliferation
CD4-positive, alpha-beta T cell proliferation
regulation of CD4-positive, alpha-beta T cell proliferation
negative regulation of CD4-positive, alpha-beta T cell proliferation
negative regulation of alpha-beta T cell proliferation
positive regulation of T cell proliferation
positive regulation of alpha-beta T cell proliferation
positive regulation of mononuclear cell proliferation
positive regulation of lymphocyte proliferation
negative regulation of T cell proliferation
negative regulation of mononuclear cell proliferation
negative regulation of lymphocyte proliferation
alpha-beta T cell proliferation
regulation of alpha-beta T cell proliferation

(D)

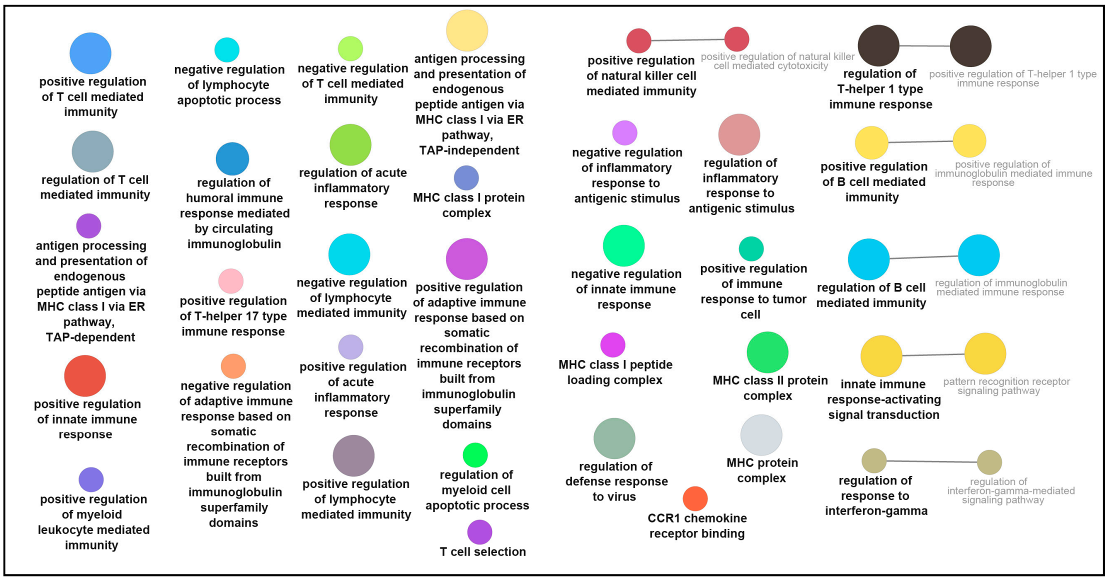
positive regulation of T cell mediated immunity
negative regulation of lymphocyte apoptotic process
negative regulation of T cell mediated immunity
antigen processing and presentation of endogenous peptide antigen via MHC class I via ER pathway, TAP-independent
positive regulation of natural killer cell mediated immunity
positive regulation of natural killer cell mediated cytotoxicity
regulation of T-helper 1 type immune response
positive regulation of T-helper 1 type immune response
regulation of T cell mediated immunity
regulation of humoral immune response mediated by circulating immunoglobulin
regulation of acute inflammatory response
MHC class I protein complex
negative regulation of inflammatory response to antigenic stimulus
regulation of inflammatory response to antigenic stimulus
positive regulation of B cell mediated immunity
positive regulation of immunoglobulin mediated immune response
antigen processing and presentation of endogenous peptide antigen via MHC class I via ER pathway, TAP-dependent
positive regulation of T-helper 17 type immune response
negative regulation of lymphocyte mediated immunity
positive regulation of adaptive immune response based on somatic recombination of immune receptors built from immunoglobulin superfamily domains
negative regulation of innate immune response
positive regulation of immune response to tumor cell
regulation of B cell mediated immunity
regulation of immunoglobulin mediated immune response
positive regulation of innate immune response
negative regulation of adaptive immune response based on somatic recombination of immune receptors built from immunoglobulin superfamily domains
positive regulation of acute inflammatory response
MHC class I peptide loading complex
MHC class II protein complex
innate immune response-activating signal transduction
pattern recognition receptor signaling pathway
positive regulation of myeloid leukocyte mediated immunity
positive regulation of lymphocyte mediated immunity
regulation of myeloid cell apoptotic process
regulation of defense response to virus
MHC protein complex
regulation of response to interferon-gamma
regulation of interferon-gamma-mediated signaling pathway
CCR1 chemokine receptor binding
T cell selection

(E)

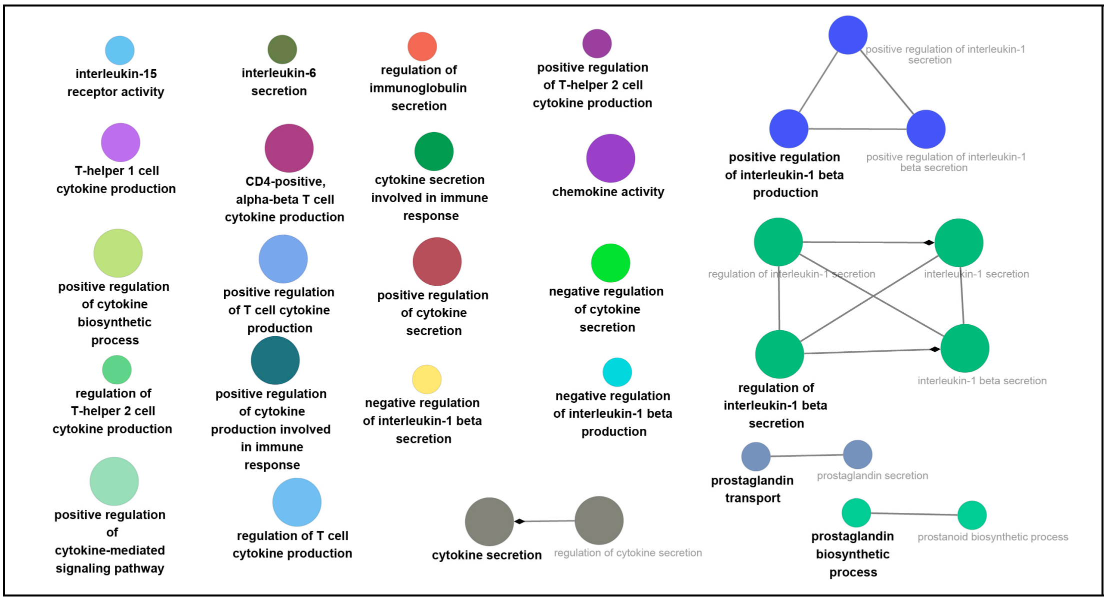

(F)

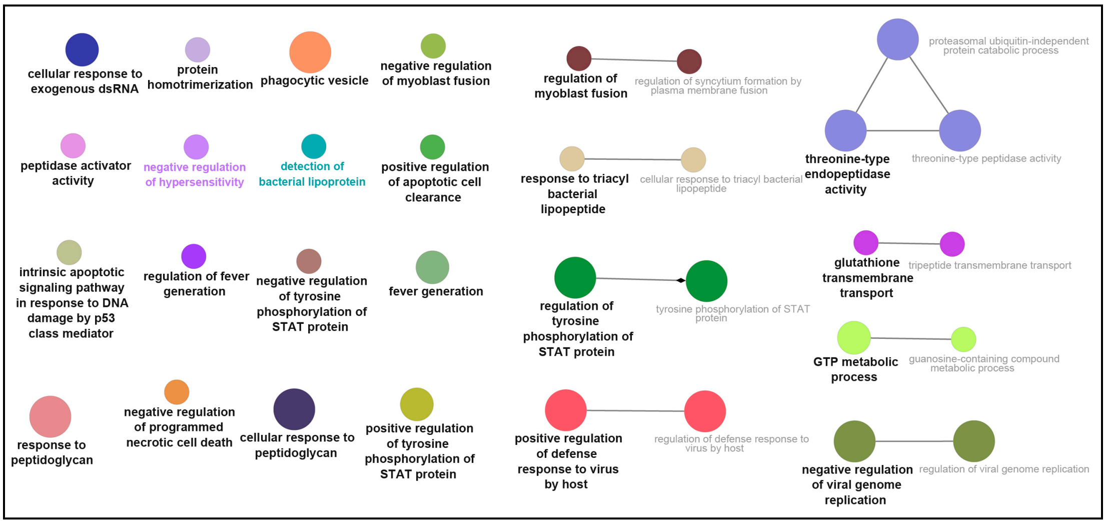

(G)

Figure S3. Functional enrichment analysis of up-regulated DEGs from ClueGO. (A) fatty acid metabolism; (B) mitochondrial function; (C) proliferation of immune cells; (D) cell proliferation; (E) cytokine production or activity; (F) inflammation and immune activity, and (G) other biological activities.
