# Supplementary material for: Transcriptomic Changes in Mouse Bone Marrow-Derived Macrophages Exposed to Neuropeptide FF
Source: Genes (Basel). 2021 May 9;12(5):705. doi: 10.3390/genes12050705 (PMC8151073; doi:10.3390/genes12050705)
Supplement: Supplementary file 1 [file genes-12-00705-s001.zip › genes-1147651-supplementary/Figure S4 Ramachandran plot.pdf]

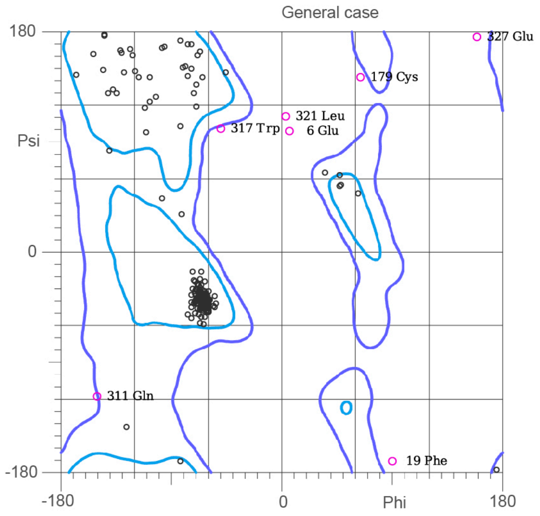

CNR2

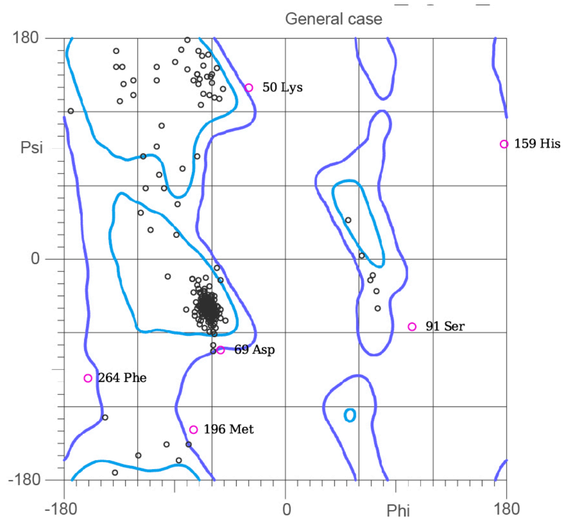

GPR55

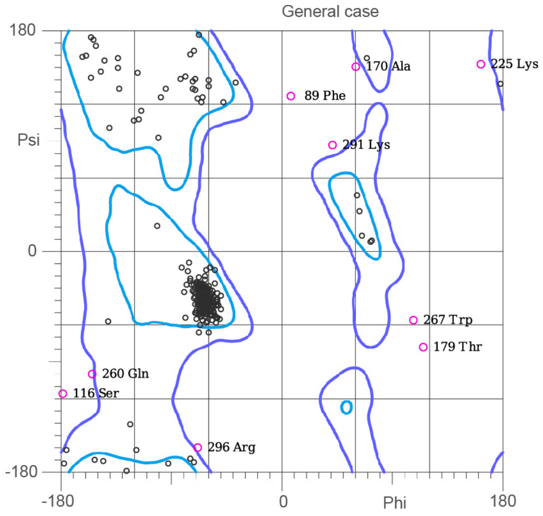

GPR18

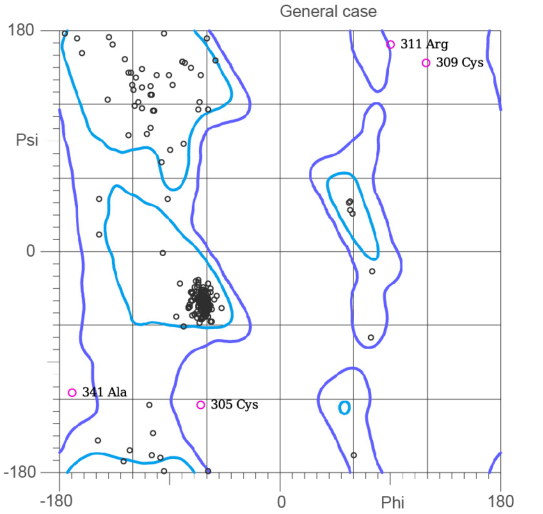

HCAR2

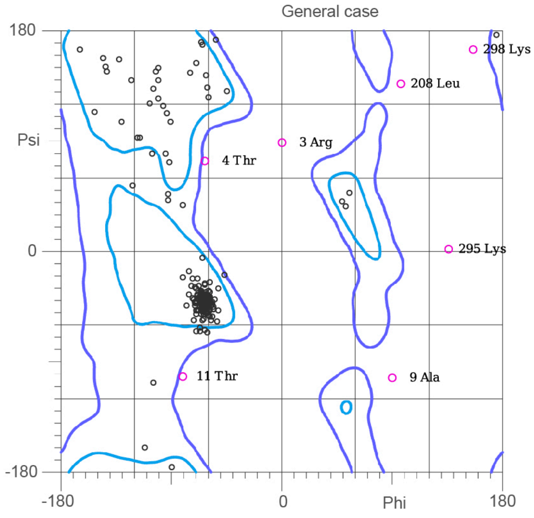

GPR31B

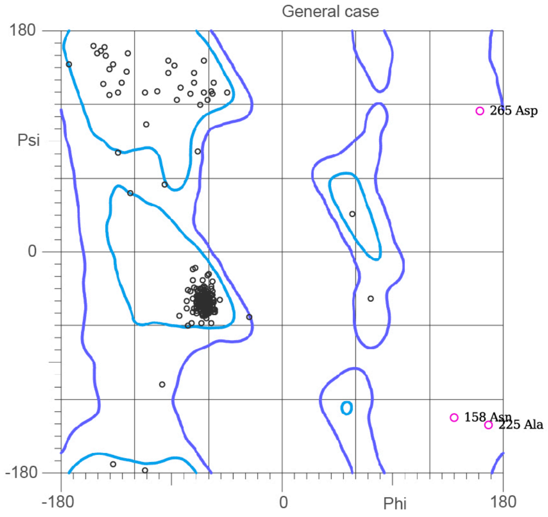

GPR183

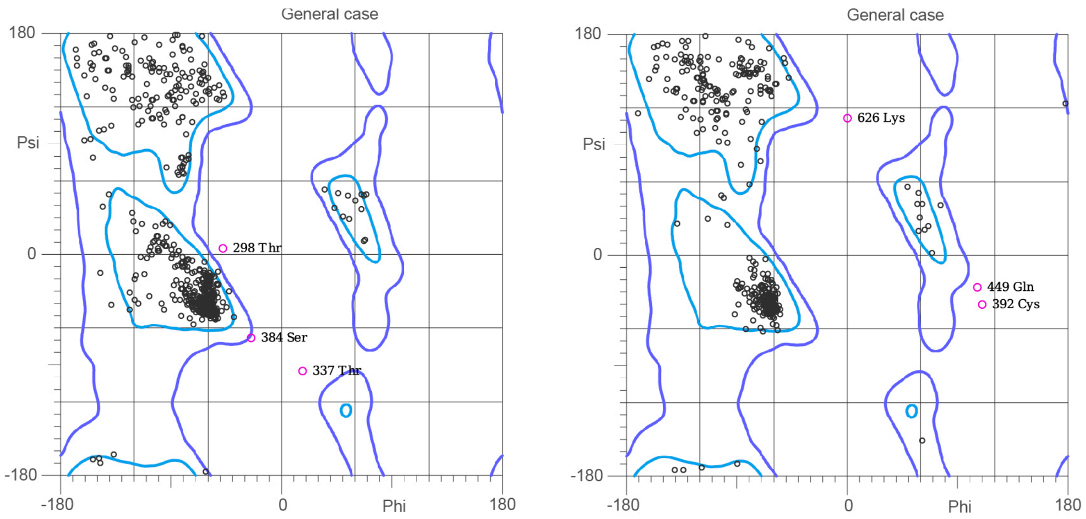

OAS2 DHX58

Figure S4. Ramachandran analysis of the Modeller-generated models of hub proteins (CNR2, GPR55, GPR18, HCAR2, GPR31B, GPR183, OAS2, and DHX58). The different colored areas: 'most favored' (light blue), 'generously allowed' (blue), and 'disallowed' (violet).
