# Supplementary material for: Transcriptomic Changes in Mouse Bone Marrow-Derived Macrophages Exposed to Neuropeptide FF
Source: Genes (Basel). 2021 May 9;12(5):705. doi: 10.3390/genes12050705 (PMC8151073; doi:10.3390/genes12050705)
Supplement: Supplementary file 1 [file genes-12-00705-s001.zip › genes-1147651-supplementary/Figure S5 3D protein alignment.pdf]

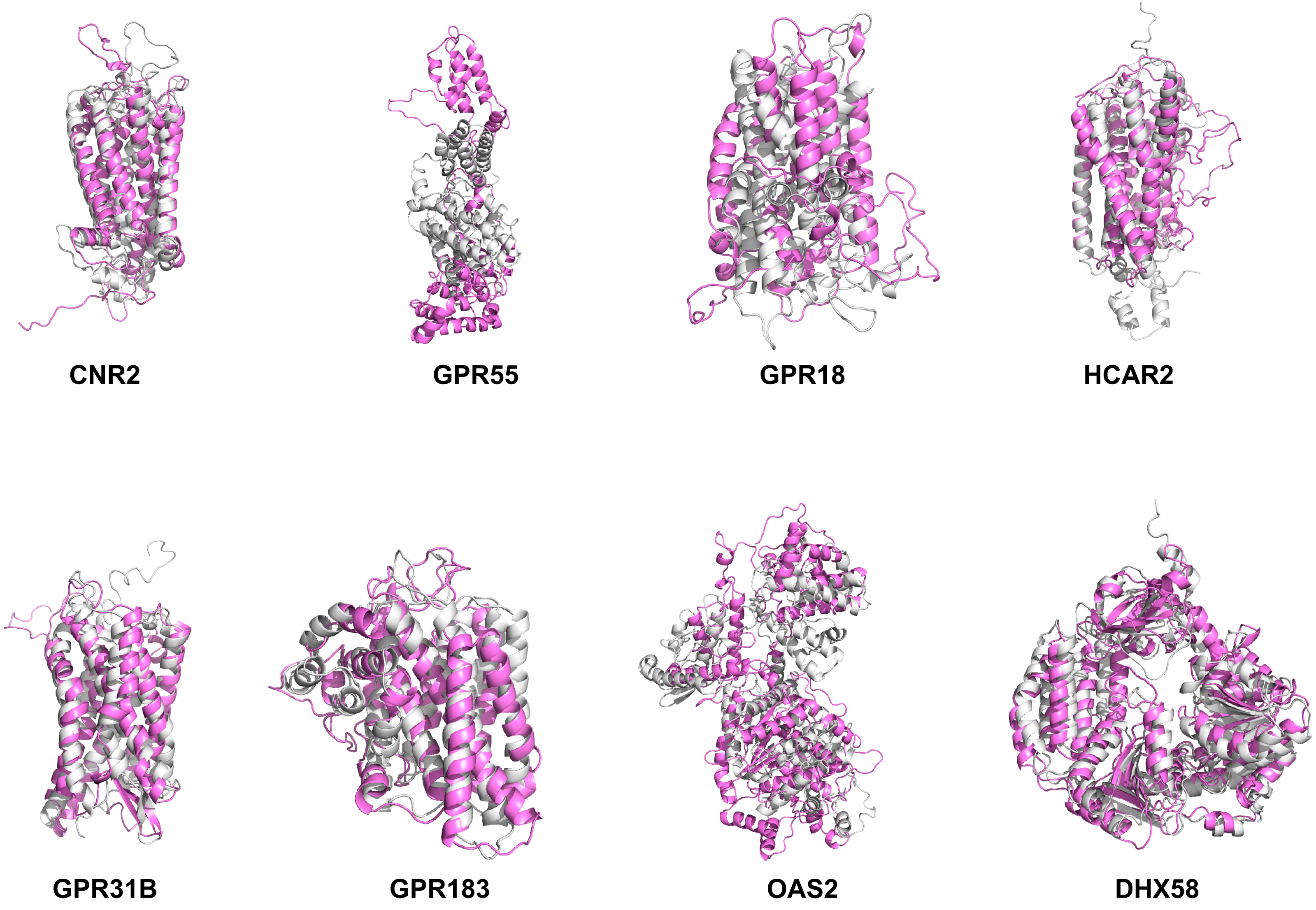
CNR2
GPR55
GPR18
HCAR2
GPR31B
GPR183
OAS2
DHX58

Figure S5. Superposition of the MD-optimized protein structure (violet) and the primarily modeled structure (gray). The picture was produced with the Pymol software (Delano, W.L. The Pymol Molecular Graphics System (2002) DeLano Scientific, SanCarlos, CA, USA. http://www.pymol.org).
