# Supplementary material for: Transcriptomic Changes in Mouse Bone Marrow-Derived Macrophages Exposed to Neuropeptide FF
Source: Genes (Basel). 2021 May 9;12(5):705. doi: 10.3390/genes12050705 (PMC8151073; doi:10.3390/genes12050705)
Supplement: Supplementary file 1 [file genes-12-00705-s001.zip › genes-1147651-supplementary/Figure S6 RMSD.pdf]

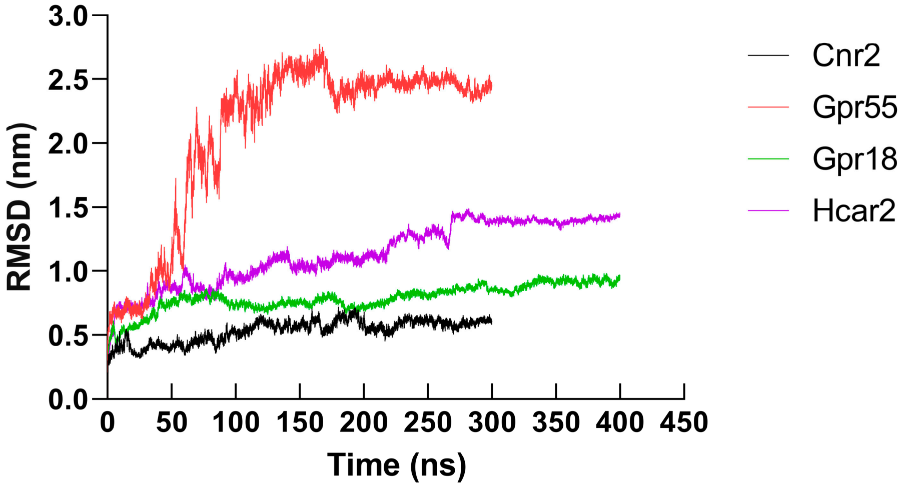

(A)

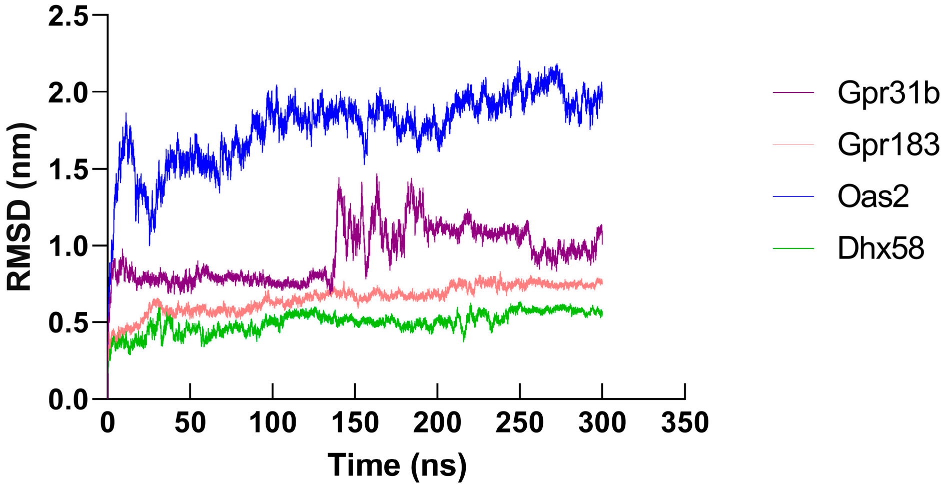

(B)

Figure S6. RMSD plots of backbone Cα atoms in molecular dynamics simulation. To show the deviations of DEGs protein clearly, RMSD plots were presented (CNR2, GPR55, GPR18, HCAR2, GPR31B, GPR183, OAS2, and DHX58).
