# Supplementary material for: Transcriptomic Changes in Mouse Bone Marrow-Derived Macrophages Exposed to Neuropeptide FF
Source: Genes (Basel). 2021 May 9;12(5):705. doi: 10.3390/genes12050705 (PMC8151073; doi:10.3390/genes12050705)
Supplement: Supplementary file 1 [file genes-12-00705-s001.zip › genes-1147651-supplementary/Figure S7 RMSF.pdf]

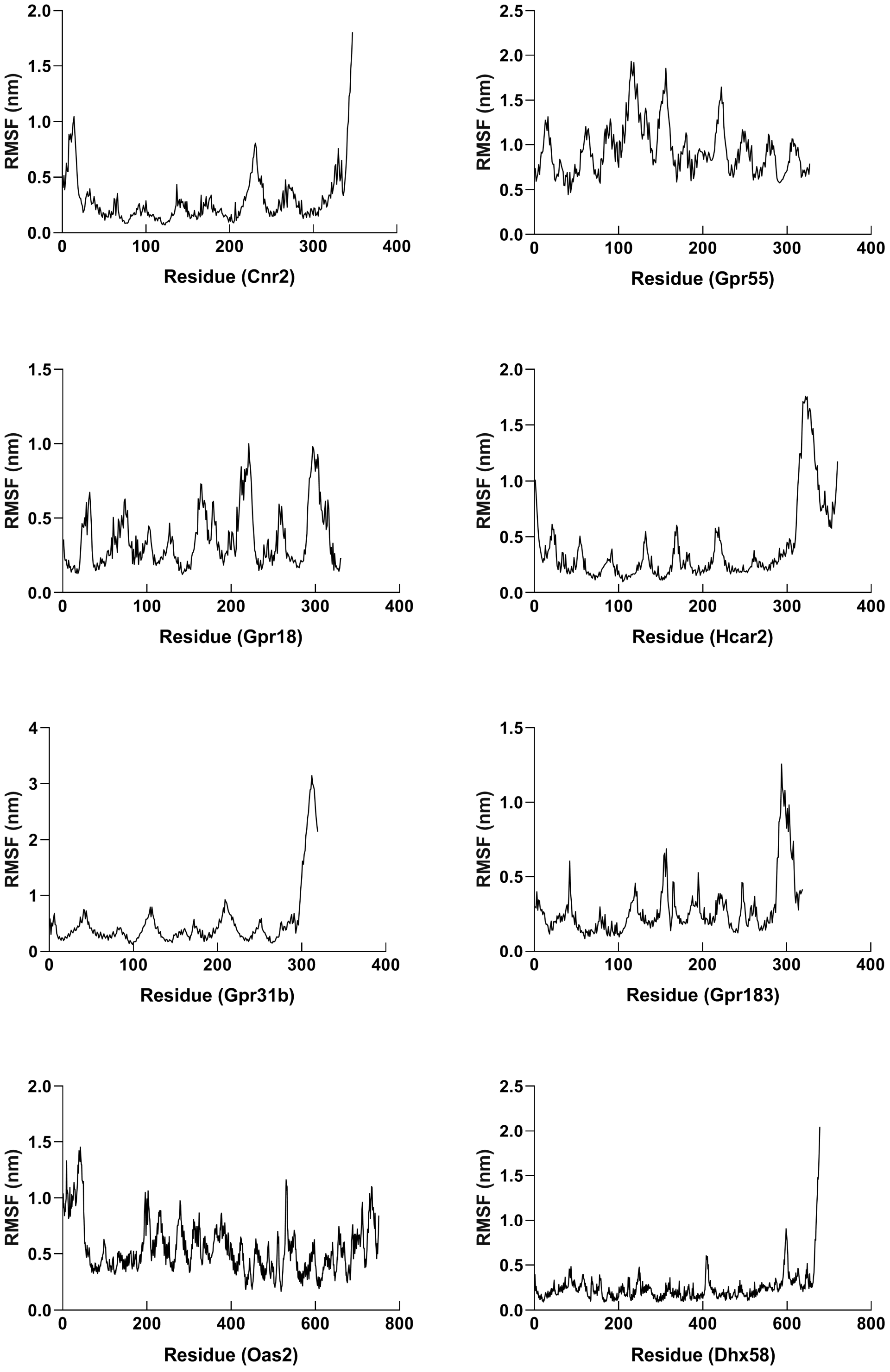

Figure S7. RMSF plots of hub proteins in molecular dynamics simulation. The RMSF of CNR2, GPR55, GPR18, HCAR2, GPR31B, GPR183, OAS2, and DHX58 were presented.
