# Supplementary material for: Transcriptomic Changes in Mouse Bone Marrow-Derived Macrophages Exposed to Neuropeptide FF
Source: Genes (Basel). 2021 May 9;12(5):705. doi: 10.3390/genes12050705 (PMC8151073; doi:10.3390/genes12050705)
Supplement: Supplementary file 1 [file genes-12-00705-s001.zip › genes-1147651-supplementary/Figure S9 3D protein.pdf]

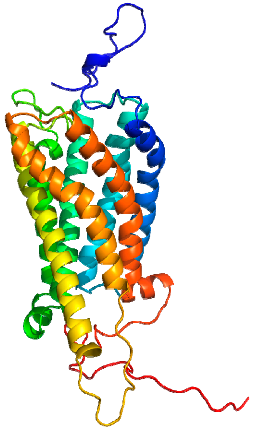

CNR2

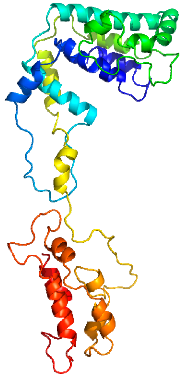

GPR55

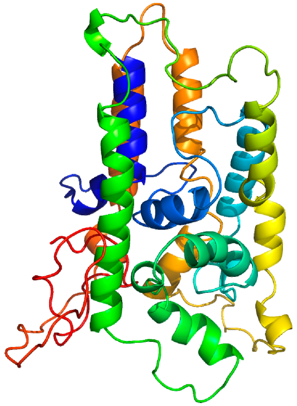

GPR18

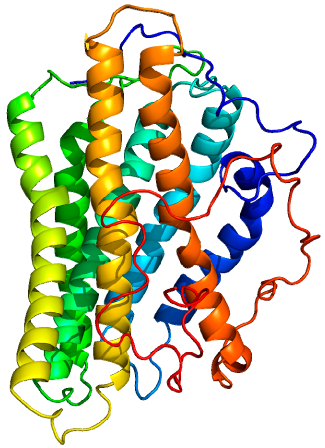

HCAR2

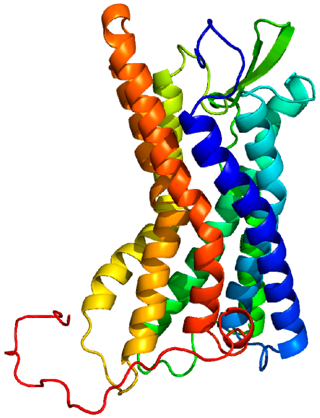

GPR31B

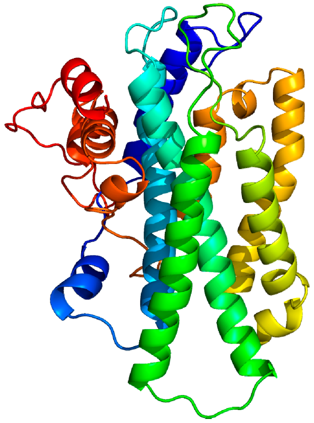

GPR183

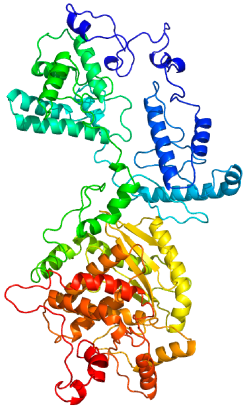

OAS2

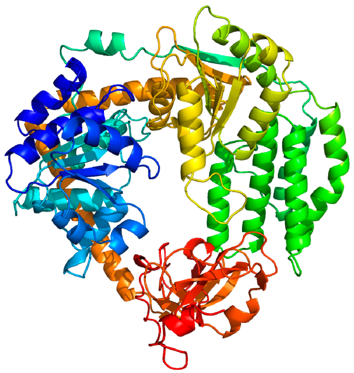

DHX58

Figure S9. The 3-D structure of hub proteins. There dimensional structures of MD-optimized hub proteins were presented, including CNR2, GPR55, GPR18, HCAR2, GPR31B, GPR183, OAS2, and DHX58. The picture was generated using the Pymol software (Delano, W.L. The Pymol Molecular Graphics System (2002) DeLano Scientific, SanCarlos, CA, USA. http://www.pymol.org).
