# Supplementary material for: Transcriptomic Changes in Mouse Bone Marrow-Derived Macrophages Exposed to Neuropeptide FF
Source: Genes (Basel). 2021 May 9;12(5):705. doi: 10.3390/genes12050705 (PMC8151073; doi:10.3390/genes12050705)
Supplement: Supplementary file 1 [file genes-12-00705-s001.zip › genes-1147651-supplementary/Supplementary file 1_Materials Manuscript.pdf]

Supplementary Materials

Transcriptomic Changes in Mouse Bone Marrow-Derived Macrophages Exposed to Neuropeptide FF

Yulong Sun [1]*, Yuanyuan Kuang [1], Zhuo Zuo[1]

Affiliations:

[1] School of Life Sciences, Northwestern Polytechnical University, Xi'an, Shaanxi Province, 710072, China.

[2] Key Laboratory for Space Biosciences & Biotechnology, Institute of Special Environmental Biophysics, School of Life Sciences, Northwestern Polytechnical University, Xi'an, Shaanxi Province, 710072, China.

***Corresponding author:**

Yulong Sun, Ph.D.

Address: Key Laboratory for Space Biosciences & Biotechnology, Institute of Special Environmental Biophysics, School of Life Sciences, Northwestern Polytechnical University, Xi'an, Shaanxi Province, 710072, China

Tel.: +86 29 88460332, Fax: +86 29 88460332, E-mail: yulongsun@nwpu.edu.cn

List of contents:

1. Supplementary Methods and Materials

2. Supplementary Results

1. Supplementary Methods and Materials

1.1 Homology Modeling of Hub Proteins

The three-dimensional structures of hub proteins were constructed via homology modeling (due to the lack of sufficient templates, the modeling of Oas2 was accomplished by Robetta *de novo* structure prediction program [5, 7]). In brief, protein sequences were retrieved from the NCBI nucleotide database (https://www.ncbi.nlm.nih.gov/protein/), and the blast module was used to align the hub protein sequence in the PDB database [2]. Protein templates were downloaded from the RCSB PDB Protein Data Bank (Table 10). Firstly, three templates (query cover > 45%) for each protein were employed for homology modeling. Subsequently, three-dimensional models of the hub protein were built with Modeller (9v23) [6]. Multiple template modeling modules were employed, such as Salign, Align2d, and Model. For each protein, one thousand candidate three-dimensional models were constructed, and the model with the lowest discrete optimized protein energy (DOPE) value was chosen. Finally, the quality of all models was examined by the online tool MolProbity (http://molprobity.biochem.duke.edu/) [4].

1.2 Molecular Dynamics (MD) Simulation

The molecular dynamics simulation of the 3-D model of the hub protein was carried out with Gromacs 2018.12 [1]. All simulations were conducted in the CHARMM36 force field [3].

1.2.1 Molecular Dynamic Simulation: Protein in Water

Molecular dynamics simulation was conducted in a similar condition with minor changes. The protein model was completely solvated in an octahedron box with TIP3P water models (1.0 nm). The whole simulate system was neutralized by adding Na^+ or Cl^- ions and periodic boundary conditions (PBC) were introduced in all directions. Energy minimization of the protein model was performed with the steepest descent (50,000 steps) with the max force (< 100 KJ/mol). Next, the whole system was simulated under equilibration phases with NVT (100 ps, 298.15 K) and NPT (300 ps, 298.15 K, 1.0 bar), respectively. Molecular dynamics simulation was performed at least 300 ns for each hub protein.

1.2.2 Molecular Dynamic Simulation: Analysis

The MD trajectory from MD simulation was interpreted with GROMACS utilities

to acquire the radius of gyration (Rg), RMSD (root mean square deviation), and RMSF (root mean square fluctuation). The three-dimensional protein structures of hub proteins were presented by using the Pymol software (Delano, W.L. The Pymol Molecular Graphics System (2002) DeLano Scientific, SanCarlos, CA, USA. http://www.pymol.org).

2. Supplementary Results

2.1 Protein Modeling of Hub Proteins

To further explore the protein structure of hub proteins, homology modeling was utilized to construct the three-dimensional structure of hub proteins with the multi-template-based protein modeling approach of Modeller (9v23). For each hub protein, a total of 1000 models were generated and the model with the lowest DOPE score was chosen for subsequent analysis (CNR2: -43164.54688; GPR55: -36958.76172; GPR18: -39193.30469; HCAR2: -44731.09766; GPR31B: -37279.40625; GPR183: -38402.71875; DHX58: -80242.11719) (Figure S3) (Table S10).

Next, the structure files of the protein models were subjected to the online tool MolProbity to assess the quality of the protein models. As demonstrated in Figure S2 and Table S11, the value of residues in the outlier region of proteins ranged from 0.01% to 0.04% (CNR2: 0.04%; GPR55: 0.03%; GPR18: 0.04%; HCAR2: 0.02%; GPR31B: 0.03%; GPR183: 0.02%; OAS2: 0.01%; and DHX58: 0.01%), hinting that the quality of these models was acceptable.

2.2 Molecular dynamics simulation

Molecular dynamics (MD) simulation (at least 300 ns) was conducted to investigate the behavior of the hub protein on a microscopic scale. The MD-optimized 3-D protein models were finally acquired (Figure 9). The structural convergence results of hub proteins were shown as RMSD (Figure S4), RMSF (Figure S5), and gyrate (Figure S6 and Table S12).

To explore the structures and dynamics of hub proteins, the RMSD of hub protein atoms was analyzed. As demonstrated in Figure S4, the backbone atom reached equilibrium state within 2.36 ns-6.11 ns from the initial stage (CNR2: 3.79

ns; GPR55: 4.28 ns; GPR18: 2.27 ns; HCAR2: 6.11 ns; GPR31B: 2.34 ns; GPR183: 2.36 ns; OAS2: 2.51 ns; and DHX58: 3.34 ns). Next, the structure began to converge at different time stages (CNR2: 9.40 ns; GPR55: 4.77 ns; GPR18: 12.05 ns; HCAR2: 37.96 ns; GPR31B: 2.52 ns; GPR183: 20.06 ns; OAS2: 68.16 ns; and DHX58: 3.52 ns). The structure of the hub proteins kept a stable conformation till the end of MD simulation (CNR2: 0.50-0.60; GPR55: 2.47-2.51; GPR18: 0.81-0.96; HCAR2: 1.40-1.44; GPR31B: 0.99-1.02; GPR183: 0.69 -0.77; OAS2: 1.85-1.99; and DHX58: 0.49-0.55).

In order to examine the flexibility of hub protein structure, the RMSF of the atoms of hub proteins was interpreted (Figure S5). Residues with high RMSF indicated large flexibility, while low RMSF values hinted few fluctuations between residues and average positions. As demonstrated in Figure S5 and Table S12, the average RMSF value varies from 0.2520 and 0.9451 (CNR2: 0.2949; GPR55: 0.9451; GPR18: 0.3580; HCAR2: 0.3893; GPR31B: 0.5039; GPR183: 0.2814; OAS2: 0.5474; and DHX58: 0.2520).

The radius of gyration (Rg) indicated the degree of compression of the protein structure, whereas a decrease in Rg represented a attenuation in the stability of the protein structure. As demonstrated in Figure S6, the average Rg value of hub protein varies from 2.100 to 3.516 (CNR2: 2.393; GPR55: 3.214; GPR18: 2.167; HCAR2: 2.274; GPR31B: 2.261; GPR183: 2.100; OAS2: 3.516; and DHX58: 2.768).

REFERENCES

[1] Abraham MJ, Murtola T, Schulz R, Páll S, Smith JC, Hess B, et al. GROMACS: High performance molecular simulations through multi-level parallelism from laptops to supercomputers. 2015;1:19-25.

[2] Berman HM, Westbrook J, Feng Z, Gilliland G, Bhat TN, Weissig H, et al. The Protein Data Bank. Nucleic Acids Res. 2000;28:235-42.

[3] Huang J, MacKerell AD, Jr. CHARMM36 all-atom additive protein force field: validation based on comparison to NMR data. Journal of computational chemistry. 2013;34:2135-45.

[4] Lovell SC, Davis IW, Arendall WB, 3rd, de Bakker PI, Word JM, Prisant MG, et al. Structure validation by Calpha geometry: phi,psi and Cbeta deviation. Proteins. 2003;50:437-50.
[5] Raman S, Vernon R, Thompson J, Tyka M, Sadreyev R, Pei J, et al. Structure prediction for CASP8 with all-atom refinement using Rosetta. Proteins. 2009;77 Suppl 9:89-99.
[6] Sali A, Blundell TL. Comparative protein modelling by satisfaction of spatial restraints. J Mol Biol. 1993;234:779-815.
[7] Song Y, DiMaio F, Wang RY, Kim D, Miles C, Brunette T, et al. High-resolution comparative modeling with RosettaCM. Structure. 2013;21:1735-42.
