# Supplementary material for: Transcriptomic Changes in Mouse Bone Marrow-Derived Macrophages Exposed to Neuropeptide FF
Source: Genes (Basel). 2021 May 9;12(5):705. doi: 10.3390/genes12050705 (PMC8151073; doi:10.3390/genes12050705)
Supplement: Supplementary file 1 [file genes-12-00705-s001.zip › genes-1147651-supplementary/Table S10 Proteins modelling.pdf]

Table S10. Protein modeling templates

Proteins	Species	Protein length (aa)	Model templates (query cover, identify)
Cnr2	Mus musculus	347	6KPF_R (2.90 Å) (100%, 82.71%)<br>6PT0_R (3.20 Å) (100%, 82.71%)<br>5ZTY_A (2.80 Å) (86%, 84.16%)
Gpr55	Mus musculus	327	5XSZ_A (3.20 Å) (59%, 35.44%)<br>4YAY_A (2.90 Å) (88%, 27.12%)<br>4ZUD_A (2.80 Å) (88%, 27.12%)
Gpr18	Mus musculus	331	5XSZ_A (3.20 Å) (63%, 31.39%)<br>6WWZ_R (3.34 Å) (88%, 27.39%)<br>6RZ6_A (2.43 Å) (66%, 27.13%)
Hcar2	Mus musculus	360	4XNV_A (2.20 Å) (84%, 24.59%)<br>6WWZ_R (3.34 Å) (86%, 27.36%)<br>6RZ6_A (2.43 Å) (45%, 36.36%)
Gpr31b	Mus musculus	319	6WWZ_R (3.34 Å) (93%, 26.95%)<br>6RZ6_A (2.43 Å) (56%, 30.77%)<br>4XNV_A (2.20 Å) (90%, 23.34%)
Gpr183	Mus musculus	357	5LWE_A (2.80 Å) (90%, 28.40%)<br>4YAY_A (2.90 Å) (86%, 27.80%)<br>4ZUD_A (2.80 Å) (85%, 27.42%)
Oas2	Mus musculus	751	*De novo* by Rosetta
Dhx58	Mus musculus	678	5JB2_A (2.20 Å) (99%, 51.47%)<br>5JAJ_A (1.50 Å) (99%, 51.47%)<br>5JC3_A (2.60 Å) (97%, 42.67%)
