# Supplementary material for: Transcriptomic Changes in Mouse Bone Marrow-Derived Macrophages Exposed to Neuropeptide FF
Source: Genes (Basel). 2021 May 9;12(5):705. doi: 10.3390/genes12050705 (PMC8151073; doi:10.3390/genes12050705)
Supplement: Supplementary file 1 [file genes-12-00705-s001.zip › genes-1147651-supplementary/Table S11 Ramachandran plot analysis.pdf]

Table S11. Ramachandran plot analysis

Proteins	Number of residues in favoured regions	Number of residues in allowed region	Number of outliers
Cnr2	328/345 (95.1%)	338/345 (98.0%)	7 (0.04%)
Gpr55	287/325 (88.3%)	314/325 (96.6%)	11 (0.03%)
Gpr18	296/329 (90.0%)	315/329 (95.7%)	14 (0.04%)
Hcar2	333/358 (93.0%)	352/358 (98.3%)	6 (0.02%)
Gpr31b	289/317 (91.2%)	306/317 (96.5%)	11 (0.03%)
Gpr183	298/317 (94.0%%)	310/317 (97.8%)	7 (0.02%)
Oas2	725/749 (96.8%)	745/749 (99.5%)	4 (0.01%)
Dhx58	656/676 (97.0%)	670/676 (99.1%)	6 (0.01%)

Note: part of the N-terminal random coil sequence of the hub proteins were removed for subsequent molecular dynamics simulation.
