# Supplementary material for: Transcriptomic Changes in Mouse Bone Marrow-Derived Macrophages Exposed to Neuropeptide FF
Source: Genes (Basel). 2021 May 9;12(5):705. doi: 10.3390/genes12050705 (PMC8151073; doi:10.3390/genes12050705)
Supplement: Supplementary file 1 [file genes-12-00705-s001.zip › genes-1147651-supplementary/Table S12_RMSD-RMSF-Rg.pdf]

Table S12. Average value of RMSD, RMSF, and Rg

Protein	RMSD	RMSF	Rg
CNR2	0.5307	0.2949	2.393
GPR55	2.074	0.9451	3.214
GPR18	0.7873	0.3580	2.167
HCAR2	1.137	0.3893	2.274
GPR31B	0.9397	0.5039	2.261
GPR183	0.6548	0.2814	2.100
OAS2	1.767	0.5474	3.516
DHX58	0.4979	0.2520	2.768
