# Supplementary material for: Transcriptomic Changes in Mouse Bone Marrow-Derived Macrophages Exposed to Neuropeptide FF
Source: Genes (Basel). 2021 May 9;12(5):705. doi: 10.3390/genes12050705 (PMC8151073; doi:10.3390/genes12050705)
Supplement: Supplementary file 1 [file genes-12-00705-s001.zip › genes-1147651-supplementary/Table S2 Details of DEGs-protein coding.pdf]

Table S2. Details of DEGs-protein_coding (up-regulated and down-regulated 24 protein_coding genes were shown)

Gene_symbol	log2FoldChange	Padj	Gene_chrosome	Change
Gabbr2	-8.295323959	1.68E-10	4	Down
Slco2b1	-6.877748635	6.15E-14	7	Down
Gprc5c	-6.19472407	3.13E-14	11	Down
Tgfb3	-6.093676657	5.35E-05	12	Down
Zbtb16	-6.046195216	1.41E-09	9	Down
Dkk2	-5.958083038	2.64E-13	3	Down
Myadml2	-5.257189141	0.003465987	11	Down
Hpgd	-5.12511186	9.48E-34	8	Down
Yipf7	-5.058485274	0.003829747	5	Down
Gpr34	-5.054495243	4.86E-05	X	Down
Nanos1	-5.005114351	8.82E-11	19	Down
Cd300lg	-4.994420895	0.009171368	11	Down
Arhgap32	-4.925683387	0.00013605	9	Down
Siglec15	-4.900925577	0.021239298	18	Down
Hmga2	-4.887729528	4.54E-48	10	Down
Cfap161	-4.836257651	0.008292963	7	Down
Pde1c	-4.836199321	0.000241315	6	Down
Tox2	-4.774848185	4.51E-59	2	Down
Chst3	-4.679708581	1.45E-12	10	Down
Rtn4rl1	-4.659066204	1.11E-05	11	Down
Rnf150	-4.543777816	6.24E-109	8	Down
Sox4	-4.516426906	4.28E-11	13	Down
Slc16a14	-4.473892336	0.023983569	1	Down
Mrap	-4.471313135	0.024001949	16	Down
Saa3	11.18204747	3.66E-190	7	Up
Cfb	10.96168234	1.69E-17	17	Up
Lad1	10.51630095	2.70E-17	1	Up
Nos2	10.31126139	9.84E-16	11	Up
Zfp811	10.05127524	1.18E-15	17	Up
Susd2	9.706574036	6.84E-45	10	Up
Acod1	9.459532943	0	14	Up
Cxcl3	9.435925205	2.01E-101	5	Up
Csf3	9.016380572	1.65E-12	11	Up
Ccl5	8.965357213	7.55E-154	11	Up
Lcn2	8.847988836	4.76E-69	2	Up
Cxcl10	8.402048341	2.42E-107	5	Up
Il6	8.390286243	1.20E-10	5	Up
Fpr1	8.230297765	3.37E-176	17	Up
Fpr2	8.133897304	1.03E-183	17	Up
Marco	8.022149335	8.38E-203	1	Up
Apol9b	7.949950375	2.45E-12	15	Up
Cd38	7.904286175	5.13E-91	5	Up
Rasgrp1	7.899061259	1.86E-56	2	Up
Gfi1	7.700044001	5.84E-12	5	Up

Fpr3	7.698803818	1.21E-08	17	Up
Clec4e	7.675562787	0	6	Up
Cxcl1	7.670454709	5.16E-70	5	Up
Pde10a	7.660679317	2.73E-08	17	Up
