# Supplementary material for: Transcriptomic Changes in Mouse Bone Marrow-Derived Macrophages Exposed to Neuropeptide FF
Source: Genes (Basel). 2021 May 9;12(5):705. doi: 10.3390/genes12050705 (PMC8151073; doi:10.3390/genes12050705)
Supplement: Supplementary file 1 [file genes-12-00705-s001.zip › genes-1147651-supplementary/Table S3 Details of DEGs-antisense.pdf]

Table S3. Details of DEGs-antisense RNA

Gene_symbol	log2FoldChange	Padj	Gene_chrosome	Change
Gm20560	-4.477696393	0.02838732	6	Down
Gm48001	-4.031365657	0.034158753	12	Down
Gm13184	-3.878019472	0.048549888	2	Down
Gm16183	-3.732008185	0.014807924	8	Down
Gm42848	-3.396692793	0.000395032	5	Down
Gm47815	-3.070557542	9.80E-06	10	Down
5033421B08Rik	-3.046488446	8.32E-05	12	Down
Gm48837	-2.872108966	0.017640377	13	Down
Gm28800	-2.689669926	0.000173113	1	Down
Gm9888	-2.480965706	0.00052299	9	Down
Dnmt3aos	-2.47570737	2.80E-18	12	Down
Gm44710	-2.206127545	0.008721769	7	Down
4930539J05Rik	-2.137814059	0.015358942	3	Down
Gm21057	-2.055240025	0.023860499	7	Down
Gm10602	-1.998756748	0.001107735	7	Down
Tnfsf13os	-1.936374481	0.000163157	11	Down
1700008B11Rik	-1.866257764	0.044415835	5	Down
Far1os	-1.834662754	0.048197804	7	Down
Gm45449	-1.759760296	0.010465368	8	Down
F630028O10Rik	-1.73560559	1.01E-42	X	Down
Gm13963	-1.692248954	0.049854509	2	Down
Gm45820	-1.670446256	0.01101733	8	Down
Gm44206	-1.645266865	0.028506013	6	Down
Gm26947	-1.570075177	9.07E-16	10	Down
4930473A02Rik	-1.54872006	0.003456538	2	Down
Gm26740	-1.469030632	3.99E-05	10	Down
C920021L13Rik	-1.459186267	0.020840888	3	Down
Gm13091	-1.43528153	0.001984295	4	Down
Gm30329	-1.422826899	2.75E-09	8	Down
4930481A15Rik	-1.411291849	8.98E-05	19	Down
C230037L18Rik	-1.302542029	0.010522554	15	Down
6330562C20Rik	-1.302312567	0.005422527	3	Down
Tmem147os	-1.224910449	0.006126168	7	Down
Gm15545	-1.088971597	0.026191345	7	Down
Gm16685	7.323607967	1.13E-07	3	Up
Gm15987	7.072521673	3.77E-07	6	Up
Gm13822	6.91522192	2.61E-06	5	Up
Gm13571	6.746027348	5.21E-06	2	Up
Gm20655	5.681033575	0.00059409	10	Up
Gm16015	5.646085952	0.003802181	5	Up
Gm26693	5.252898344	0.009546542	17	Up
A630012P03Rik	5.14203699	0.004040299	X	Up
A730090N16Rik	4.488866005	0.037443696	3	Up
Gm12764	4.226445974	0.02719468	7	Up
Gm10552	4.160886344	0.0336598	1	Up
Taco1os	4.086733481	0.023402431	11	Up

Gm43221	4.077213772	0.027065588	3	Up
Gm44418	3.796206852	4.85E-05	6	Up
4930512H18Rik	3.603696858	1.12E-05	8	Up
AC113595.1	2.723430583	2.84E-10	15	Up
Gm15523	2.609735929	0.027556865	12	Up
Gm20234	2.379881272	0.000739913	11	Up
Gm15512	2.370590804	0.003706703	14	Up
Gm4117	2.284853636	0.006518447	13	Up
Gm15133	2.265397889	0.0034075	7	Up
Gm20658	2.173694888	9.38E-25	12	Up
1700058P15Rik	2.086355402	7.84E-09	7	Up
Gm11131	2.060568074	1.56E-06	17	Up
Gm11772	1.891805161	0.000105395	11	Up
A530040E14Rik	1.846959476	1.15E-07	1	Up
Gm15601	1.824517555	6.02E-09	14	Up
Gm10425	1.804278209	4.04E-07	12	Up
Gm16174	1.796568511	0.043264517	7	Up
9530082P21Rik	1.748347512	0.00018501	17	Up
1700071M16Rik	1.472811487	0.035532138	17	Up
Gm17017	1.371005961	0.036156061	1	Up
Gm13205	1.234863846	0.039889331	4	Up
Gm17435	1.117559582	0.022453251	8	Up
Gm37787	1.106567929	0.016016273	1	Up
AC168220.3	1.064416591	0.021879086	16	Up
