# Supplementary material for: Transcriptomic Changes in Mouse Bone Marrow-Derived Macrophages Exposed to Neuropeptide FF
Source: Genes (Basel). 2021 May 9;12(5):705. doi: 10.3390/genes12050705 (PMC8151073; doi:10.3390/genes12050705)
Supplement: Supplementary file 1 [file genes-12-00705-s001.zip › genes-1147651-supplementary/Table S4 Details of DEGs-miRNA.pdf]

Table S4. Details of DEGs-miRNA

Gene_symbol	log2FoldChange	Padj	Gene_chrosome	Change
Mir7678	4.784597165	0.02063689	2	Up
Mir5107	2.847311685	0.025899382	18	Up
Gm27514	2.770965467	0.049432846	6	Up
Gm22655	-5.014771744	7.43E-09	7	Down
