# Supplementary material for: Transcriptomic Changes in Mouse Bone Marrow-Derived Macrophages Exposed to Neuropeptide FF
Source: Genes (Basel). 2021 May 9;12(5):705. doi: 10.3390/genes12050705 (PMC8151073; doi:10.3390/genes12050705)
Supplement: Supplementary file 1 [file genes-12-00705-s001.zip › genes-1147651-supplementary/Table S5 Details of DEGs-lincRNA.pdf]

Table S5. Details of DEGs-lincRNA

Gene_symbol	log2FoldChange	Padj	Gene_chrosome	Change
Gm16701	-5.126604061	0.003623101	1	Down
F730035P03Rik	-4.250150125	0.04175541	7	Down
Gm45847	-3.666042933	0.003083492	7	Down
Gm32098	-3.652095939	0.000990913	8	Down
Prr33	-3.562961825	0.000104425	7	Down
C230035I16Rik	-3.520884207	0.004410299	13	Down
Gm14321	-3.382586061	0.000209103	2	Down
9230111E07Rik	-3.285892655	0.011217421	2	Down
C030034L19Rik	-3.164875214	4.71E-13	3	Down
Gm29291	-3.029453064	0.005975423	1	Down
Gm26669	-2.964902562	0.004284723	12	Down
Gm37168	-2.653557551	2.56E-08	1	Down
Gm5086	-2.634093104	1.08E-18	13	Down
Gm33103	-2.490533994	0.000849217	8	Down
Gm26890	-2.480858758	0.015855368	7	Down
Gm14221	-2.434502701	2.42E-06	2	Down
D630033O11Rik	-2.353592699	0.000297175	9	Down
AC125351.1	-2.312379431	3.31E-08	12	Down
Gm6634	-2.107551092	0.002323299	3	Down
2810455O05Rik	-2.069891009	0.007164369	8	Down
Gm26581	-2.063437421	0.029599787	10	Down
F730311O21Rik	-2.051282806	8.15E-12	1	Down
Gm13391	-2.028096446	5.74E-16	2	Down
CT030636.2	-2.008808634	0.025784211	17	Down
Gm14168	-1.788496039	0.029089616	2	Down
5033430I15Rik	-1.725782766	0.027019502	13	Down
Gm16907	-1.721117406	0.002643347	13	Down
Gm44053	-1.70633157	0.00155334	6	Down
Gm43672	-1.633535781	8.01E-05	3	Down
Gm38399	-1.599187123	0.020340894	1	Down
5031425F14Rik	-1.543963507	0.001285135	2	Down
2610035D17Rik	-1.500671555	3.30E-06	11	Down
Gm26912	-1.462380696	0.031340758	12	Down
Gm12708	-1.430923154	0.012597571	4	Down
Gm14461	-1.421249816	7.42E-12	2	Down
A230028O05Rik	-1.415214623	0.000911635	16	Down
Gm28875	-1.378293266	0.004856812	12	Down
4930480K23Rik	-1.354841253	0.025864473	14	Down
Gm45752	-1.351829107	0.000584949	8	Down
Gm15706	-1.304220026	0.028651432	6	Down
Zfp469	-1.265636393	0.000402742	8	Down
Mirt1	-1.208652324	0.000545404	19	Down
AI839979	-1.190280118	3.62E-06	5	Down
4933404O12Rik	-1.133674402	0.003168001	5	Down
Gm20342	-1.078435468	0.046859885	1	Down
AU022793	-1.04132826	1.71E-05	15	Down

Malat1	-1.000935105	0.027019502	19	Down
Mir155hg	8.003872703	6.53E-13	16	Up
AW112010	6.431920186	2.55E-76	19	Up
Gm34643	5.559101476	7.85E-64	14	Up
Gm47903	5.479951	0.00201844	10	Up
1700003M07Rik	5.472100782	0.003018506	4	Up
Gm1720	5.257889984	1.91E-07	X	Up
Gm32089	5.040831793	1.44E-06	13	Up
Gm47242	5.018134255	6.99E-06	19	Up
Gm13814	4.947074144	0.014512935	2	Up
Gm36723	4.853195458	0.017384271	12	Up
4930440I19Rik	4.749320785	0.004559454	2	Up
Gm47896	4.649036342	0.021331969	10	Up
Gm30918	4.543592315	0.035894281	13	Up
A930003A15Rik	4.448165521	0.001611213	16	Up
Gm28935	4.40785057	0.012410559	19	Up
Gm11707	4.173852513	0.000761993	11	Up
Ptgs2os2	4.099847131	0.000153995	1	Up
A330074K22Rik	3.851686632	1.84E-13	8	Up
4933432I03Rik	3.783570836	7.89E-05	14	Up
1110002J07Rik	3.396416925	1.75E-61	10	Up
Gm15675	3.330630066	3.87E-06	1	Up
Gm7644	2.991405781	0.028122236	14	Up
Gm14005	2.740490974	2.75E-12	2	Up
Gm37336	2.642118335	0.02520462	1	Up
Gm36161	2.270064125	1.26E-26	13	Up
Bvht	2.182040101	2.13E-06	18	Up
Gm41409	2.066416691	0.011298259	16	Up
Gm26981	2.054532574	0.010961957	7	Up
Gm33929	2.052764637	0.019775556	12	Up
Gm26797	1.734768763	3.40E-05	9	Up
Gm17705	1.708861448	0.000240355	17	Up
D330050G23Rik	1.665411171	0.04650436	2	Up
Gm48653	1.62818063	2.53E-09	12	Up
4833445I07Rik	1.494226188	5.73E-06	9	Up
Gm44751	1.473263173	0.013512926	7	Up
Gm46224	1.392726741	1.33E-05	10	Up
Gm11110	1.375876387	0.001689757	17	Up
Trp53cor1	1.348895792	0.042675013	17	Up
AI427809	1.316168185	0.013503108	4	Up
9330175E14Rik	1.175473937	0.000106819	8	Up
Snhg6	1.148544826	0.005884782	1	Up
Gm26637	1.103747863	0.00034153	17	Up
Gm7160	1.071486617	0.000406581	1	Up
AI504432	1.015445503	6.12E-05	3	Up
