# Supplementary material for: Transcriptomic Changes in Mouse Bone Marrow-Derived Macrophages Exposed to Neuropeptide FF
Source: Genes (Basel). 2021 May 9;12(5):705. doi: 10.3390/genes12050705 (PMC8151073; doi:10.3390/genes12050705)
Supplement: Supplementary file 1 [file genes-12-00705-s001.zip › genes-1147651-supplementary/Table S6 KEGG pathway.pdf]

Table S6. KEGG pathway analysis of DEGs

	Pathway ID	Name	Gene count	PValue	%	Genes
1	mmu05169	Epstein-Barr virus infection-Mus musculus (mouse)	24	2.34E-02	8.91	*H2-M2, Irf7, Tlr2, Tnf, H2-Q6, Nfkbie, Nfkbia, Tapbp, Tap1, Cdkn1a, Cxcl10, Cd40, H2-Q7, Relb, Icam1, H2-Q5, Isg15, Ddx58, Entpd1, Nfkb2, Oas1g, H2-Q4, Oas2, Oas3, Oas1a, Fas, Ikbke, H2-T24, H2-T22, Tnfaip3, Ccnd1, Stat2, Stat1, H2-K1, H2-Ab1, Oas1b, H2-T23, Irf9, Itgal, Tap2, H2-Aa, Nfkbib, Traf2, Jun, Cdk6, Myc, H2-M3, Calr, Mapk14, Bcl2l11, H2-DMa, Bak1, H2-Eb1, Psmd11, Eif2ak2, H2-T10, Myd88, Ccna2, Gm9574, Rela, Il6, Cd247, Sap30, Cycs, Gm10053, E2f2, Mdm2, H2-DMb1, B2m, Psmd2, Hdac1, Skp2, Psmd3, Mapk11, Rbpj, Gm11131, Adrm1, H2-D1, Cdkn1b, Psmc2, Tbk1, Ccne1, Casp9, Psmc1, Gadd45b, Psmd7, H2-Oa, Traf5, Psmc5, Syk, Map2k6, Bax, Bid, Nfkb1, Psmc4, Lyn, Psmd14, Psmd12, Psmd4, Psmc3, Psmd6, Map2k3, H2-Q10, Psmd13, Pdia3, Psmc6, Cdk2, Tyk2, Gm10093, Ccnd2, Gm9575, Ifnar2, Mapk9, Akt2, E230016M11Rik, Ikbkg, H2-Eb2, Rb1, Polk, Sem1, Stat3, Mapk12, H2-Ob, Pik3r2, Trp53, Gm7030, Ncor2*
2	mmu03050	Proteasome-Mus musculus (mouse)	127	1.29E-10	9.86	*Psme2, Psme2b, Psmb8, Psmb10, Psmd11, Psme1, Psmb9, Psmd2, Psmb5, Psma5, Gm12989, Psmd3, Psma7, Gm3375, Gm8394, Adrm1, Psma6, Psmb6, Psmc2, Psmc1, Psmb7, Psmd7, Psma3, Psmc5, Psmb3, Psma4, Psma2, Psmc4, AC114990.3, Gm13835, Pomp, Psmd14, Psmd12, Psmd4, Psma1, Psmb2, Psmc3, Psmb4, Psmd6, Psmd13, Gm6542, Psmc6, Gm9575, Psmb1, Gm4950, Sem1*
3	mmu05330	Allograft rejection-Mus musculus (mouse)	46	1.94E-08	3.57	*H2-M2, Tnf, H2-Q6, Cd40, Cd28, H2-Q7, H2-Q5, H2-Q4, Fas, H2-T24, H2-T22, H2-K1, H2-Ab1, H2-T23, H2-Aa, H2-M3, H2-DMa, H2-Eb1, H2-T10, Gm9574, Il12b, H2-DMb1, Il12a, Gm11131, H2-D1, H2-Oa, Gzmb, Cd86, Il4, H2-Q10, H2-Eb2, Cd80, Il10, H2-Ob, Gm7030*
4	mmu05167	Kaposi sarcoma-	35	6.13E-08	2.72	*H2-M2, Irf7, Cxcl2, H2-Q6, Nfkbia, C3, Cdkn1a, Cxcl3, Mapkapk2, Src, Ptgs2,*

		associated herpesvirus infection-Mus musculus (mouse)				*H2-Q7, Icam1, H2-Q5, Cxcl1, Hck, H2-Q4, Fas, Ikbke, H2-T24, H2-T22, Jak2, Ccnd1, Stat2, Pdgfb, Pik3r6, Stat1, Vegfa, H2-K1, Hif1a, H2-T23, Gng12, Irf9, Traf2, Fos, Ccr1, Jun, Cdk6, Myc, H2-M3, Mapk14, Tlr3, Bak1, Eif2ak2, H2-T10, Gm9574, Rela, Il6, Gng2, Cycs, Gngt2, Gm10053, Rcan1, Ppp3cc, E2f2, Itpr3, Mapk11, Map2k1, Ccr3, Cd200r1, Calm1, Gm11131, H2-D1, Calm3, Tbk1, Casp9, Il6st, Gnb1, Syk, Map2k6, Ubb, Bax, Bid, Nfkb1, Nfatc2, Lyn, Ppp3ca, Nfatc1, Tcf7l2, Plcg1, Map2k2, Cd86, Zhx3, H2-Q10, Gnb2, Pik3c3, Hras, Tyk2, Atg14, Ifnar2, Ppp3r1, Gm1821, Mapk9, Ppp3cb, Akt2, Ikbkg, Gm11808, Rb1, Pik3r5, Stat3, Mapk12, Atg3, Zfp36, Gng7, Ubc, Pik3r2, Trp53, Gsk3b, Gm7030, Angpt2*
5	mmu05320	Autoimmune thyroid disease-Mus musculus (mouse)	110	1.75E-07	8.54	*H2-M2, H2-Q6, Cd40, Cd28, H2-Q7, H2-Q5, H2-Q4, Fas, H2-T24, H2-T22, H2-K1, H2-Ab1, H2-T23, H2-Aa, H2-M3, H2-DMa, H2-Eb1, H2-T10, Gm9574, H2-DMb1, Gm11131, H2-D1, H2-Oa, Gzmb, Cd86, Il4, H2-Q10, H2-Eb2, Cd80, Il10, H2-Ob, Gm7030*
6	mmu04621	NOD-like receptor signaling pathway-Mus musculus (mouse)	32	4.53E-07	2.48	*Gbp2, Irf7, Gbp5, Gbp3, Tnf, Cxcl2, Ccl5, Ifi206, Nfkbia, Cxcl3, Birc3, Il1b, Nampt, Gbp7, Cxcl1, Oas1g, Mefv, Oas2, Oas3, Oas1a, Ikbke, Tank, Tnfaip3, Ripk2, Stat2, Stat1, Antxr1, Ifi204, Casp4, Oas1b, Irf9, Cybb, Nlrp3, Gsdmd, Nlrp1b, Hsp90ab1, Nod1, Nod2, Nfkbib, Traf2, Antxr2, Jun, Mapk14, Camp, Panx1, Rnasel, Ccl2, Myd88, Rela, Il6, Rnf31, Txnip, Txn1, Itpr3, Gm7019, Plcb2, Birc2, Atg5, Mapk11, Hsp90aa1, Vdac3-ps1, Tbk1, Vdac3, Trpm2, Nek7, Traf5, Naip5, Cyba, Casp1, Prkcd, Nfkb1, Pstpip1, Ywhae, Bcl2l1, Ctsb, Vdac2, Tlr4, Brcc3, Naip1, P2rx7, Gm7591, Tyk2, Naip2, Ifnar2, Mapk9, Rhoa, Plcb4, Gbp2b, Ikbkg, Trp53bp1, Tmem173, Trpv2, Naip3, Mapk12, Nlrp1c-ps*
7	mmu04940	Type I diabetes mellitus-Mus musculus (mouse)	95	4.53E-07	7.38	*H2-M2, Tnf, H2-Q6, Cd28, Il1b, H2-Q7, H2-Q5, Il1a, H2-Q4, Fas, H2-T24, H2-T22, H2-K1, H2-Ab1, H2-T23, H2-Aa, H2-M3, H2-DMa, H2-Eb1, H2-T10, Gm9574, Il12b, H2-DMb1, Il12a, Gm11131, H2-D1, Lta, H2-Oa, Hspd1, Gzmb, Cd86, H2-Q10, H2-Eb2, Ica1, Cd80, H2-Ob, Cpe, Gm7030*
8	mmu05020	Prion disease-Mus	38	6.07E-07	2.95	*Tnf, Ccl5, Il1b, Il1a, Rac2, Ncf1, Fyn, Cav1, Cybb, Stip1, Klc4, Ncf4, Mapk14,*

		musculus (mouse)				*Psmd11, Prnp, Tuba4a, Il6, Hspa5, Atp5b, Cycs, mt-Cytb, Gm10053, Cacna1d, Tubb4b, Ppp3cc, Itpr3, Psmd2, Psmb5, Cox5a, Psma5, Gm12989, Atf6b, Psmd3, Psma7, Hspa8, Tubb5, Gm3375, Mapk11, Gm8394, Adrm1, Psma6, Psmb6, Vdac3-ps1, Psmc2, Atf4, Casp9, Creb5, Psmc1, Psmb7, Vdac3, Psmd7, Cyc1, Prkacb, Kif5c, Psma3, Cyba, Cacna1b, Psmc5, Psmb3, Psma4, Prkcd, Eif2s1, Bax, Psma2, 4930481A15Rik, Psmc4, Uqcrq, AC114990.3, Ppp3ca, Gm13835, Slc25a5, Hspa1b, Psmd14, Sdhb, Psmd12, Psmd4, Cox7a2, Creb3l2, Psma1, Psmb2, Atp5g3, Psmc3, Psmb4, Csnk2b, Ndufb6, Psmd6, Ndufa11, Kif5a, Vdac2, C1qa, Atp5g1, Uqcrc2, Bad, Hspa2, Ndufb9, Psmd13, Ndufa5, Gm16418, Ndufa3, Gm6542, Psmc6, mt-Nd2, Atp5a1, Cox7b, Ndufs6, Tubb4a, Cox6a1, Ndufc1, Uqcrb, Uqcrc1, Ndufa2, Gm7591, Tuba1b, mt-Nd4l, Cox8a, Atp5e, Gm5529, Gm9575, Ppp3r1, Cox6b1, Mapk9, Ppp3cb, Ddit3, Gm8355, Ndufs4, Psmb1, Gm4950, Ndufb8, Ndufa9, Atp5f1, Ndufab1, Ndufa13, Cav2, Ndufa10, Uqcr10, Sem1, Ndufab1-ps, mt-Nd5, Atp5d, Gm10039, Ndufv2, Ndufv1, Creb3, Ndufs3, Tuba1c, Ndufb3, Mapk12, Ndufa1, Ndufb7, Tubb4b-ps1, Klc2, Atp5h, Ndufs7, Uqcr11, 4930447K03Rik, Pik3r2, Ndufa12, Ndufa8, Cox4i1, Gsk3b, Ndufs8, Cox6a2, Sdhd, Atp5j*
9	mmu05332	Graft-versus-host disease-Mus musculus (mouse)	164	1.30E-06	12.74	*H2-M2, Tnf, H2-Q6, Cd28, Il1b, H2-Q7, H2-Q5, Il1a, H2-Q4, Fas, H2-T24, H2-T22, H2-K1, H2-Ab1, H2-T23, H2-Aa, H2-M3, H2-DMa, H2-Eb1, H2-T10, Gm9574, Il6, H2-DMb1, Gm11131, H2-D1, H2-Oa, Gzmb, Cd86, H2-Q10, H2-Eb2, Cd80, H2-Ob, Gm7030*
10	mmu05012	Parkinson disease-Mus musculus (mouse)	33	2.04E-06	2.56	*Adora2a, Ube2l6, Daxx, Uba7, Pink1, Klc4, Uchl1, Psmd11, Tuba4a, Nfe2l2, Hspa5, Atp5b, Cycs, mt-Cytb, Gm10053, Tubb4b, Txn1, Itpr3, Psmd2, Psmb5, Cox5a, Psma5, Gm12989, Psmd3, Dusp1, Psma7, Tubb5, Gm3375, Calm1, Gm8394, Sncaip, Adrm1, Psma6, Psmb6, Vdac3-ps1, Calm3, Psmc2, Slc18a1, Atf4, Casp9, Psmc1, Psmb7, Vdac3, Psmd7, Cyc1, Atf6, Prkacb, Kif5c, Psma3, Psmc5, Psmb3, Psma4, Eif2s1, Ubb, Bax, Psma2, 4930481A15Rik, Psmc4, Uqcrq, AC114990.3, Gm13835, Slc25a5, Psmd14, Sdhb, Psmd12, Psmd4, Cox7a2, Plcg1, Psma1, Psmb2, Atp5g3, Xbp1, Psmc3, Psmb4, Bcl2l1, Ndufb6,*

						Psmd6, Ndufa11, Kif5a, Vdac2, Zhx3, Map3k5, Atp5g1, Uqcrc2, Ndufb9, Psmd13, Ndufa5, Gm16418, Ndufa3, Gm6542, Psmc6, mt-Nd2, Camk2b, Ube2j2, Atp5a1, Cox7b, Ndufs6, Tubb4a, Cox6a1, Ube2j1, Ndufc1, Uqcrb, Uqcrc1, Ndufa2, Gm7591, Tuba1b, mt-Nd4l, Cox8a, Atp5e, Gm5529, Gm9575, Cox6b1, Gm1821, Mapk9, Ddit3, Ndufs4, Psmb1, Gm4950, Uba1, Ndufb8, Ndufa9, Ube2g2, Park7, Atp5f1, Ndufab1, Ndufa13, Drd1, Gm11808, Ndufa10, Uqcr10, Sem1, Ndufab1-ps, mt-Nd5, Atp5d, Gm10039, Ndufv2, Ndufv1, Ndufs3, Tuba1c, Ndufb3, Ndufa1, Ndufb7, Ubc, Tubb4b-ps1, Klc2, Atp5h, Ndufs7, Uqcr11, 4930447K03Rik, Trp53, Ndufa12, Ndufa8, Cox4i1, Gnas, Ndufs8, Cox6a2, Sdhd, Atp5j
11	mmu05321	Inflammatory bowel disease-Mus musculus (mouse)	158	2.38E-06	12.27	*Tlr2, Tnf, Il1b, Il1a, Il21r, Il2rg, Stat1, H2-Ab1, H2-Aa, Nod2, Jun, H2-DMa, H2-Eb1, Rela, Il6, Il12rb1, Il12b, Ifngr2, H2-DMb1, Il12a, Rora, Il23r, H2-Oa, Tgfb3, Nfkb1, Nfatc1, Il4ra, Il4, Smad2, Tlr4, Maf, H2-Eb2, Tbx21, Stat3, Il10, H2-Ob*
12	mmu04668	TNF signaling pathway-Mus musculus (mouse)	36	3.94E-06	2.80	*Mmp14, Tnf, Traf1, Cxcl2, Ccl5, Socs3, Nfkbia, Mmp9, Cxcl10, Ifi47, Cxcl3, Irf1, Birc3, Il1b, Ptgs2, Icam1, Bcl3, Mlkl, Cxcl1, Fas, Tnfaip3, Cflar, Csf1, Nod2, Traf2, Fos, Jun, Edn1, Vcam1, Mapk14, Tnfrsf1b, Cebpb, Ccl2, Rela, Gm5431, Il6, Rps6ka4, Gm7019, Birc2, Atf6b, Il15, Mapk11, Map2k1, Lta, Atf4, Creb5, Traf5, Casp7, Map2k6, Nfkb1, Vegfc, Creb3l2, Map2k3, Map3k5, Map3k8, Gm17334, Mapk9, Akt2, Lif, Jag1, Ikbkg, Mmp3, Creb3, Mapk12, Pik3r2*
13	mmu05163	Human cytomegalovirus infection-Mus musculus (mouse)	65	5.63E-06	5.05	*H2-M2, Tnf, Ccl5, H2-Q6, Nfkbia, Tapbp, Tap1, Cdkn1a, Src, Il1b, Ptgs2, H2-Q7, H2-Q5, H2-Q4, Rac2, Fas, Ccl3, H2-T24, H2-T22, Ccnd1, Cxcr4, Vegfa, H2-K1, H2-T23, Gng12, Tap2, Traf2, Gna12, Ccr1, Cdk6, Myc, Itgb3, H2-M3, Ccl4, Calr, Mapk14, Bak1, H2-T10, Ccl2, Gm9574, Rela, Il6, Gng2, Cycs, Gngt2, Gm10053, Adcy3, Ppp3cc, E2f2, Mdm2, Itpr3, B2m, Plcb2, Atf6b, Mapk11, Map2k1, Ccr3, Calm1, Gm11131, H2-D1, Calm3, Mb21d1, Tbk1, Atf4, Casp9, Creb5, Gnb1, Traf5, Prkacb, Itgav, Ptger4, Map2k6, Bax, Prkcb, Bid, Nfkb1, Nfatc2, Ppp3ca, Il6ra, Nfatc1, Creb3l2, Map2k2, Adcy7, Adcy9, H2-Q10, Gnb2, Pdia3, Gm12188, Hras, Bcar1, Ppp3r1, Ptk2, Ppp3cb, Rhoa, Akt2, Plcb4, Prkcg, Eif4ebp1, Ikbkg, Tmem173, Rb1, Tsc1, Adcy2, Creb3, Stat3, Mapk12, Gng7,*

						Pik3r2, Trp53, Pxn, Gsk3b, Gm7030, Gnas
14	mmu05010	Alzheimer disease-Mus musculus (mouse)	113	5.63E-06	8.77	*Tnf, Il1b, Ptgs2, Il1a, Lpl, Fas, Capn2, Cybb, Csf1, Traf2, Irs2, Nos2, Klc4, Fzd1, Fzd7, Psmd11, Dkk2, Tuba4a, Eif2ak2, Wnt6, Csnk1e, Rela, Aph1c, Il6, Atp5b, Adam17, Cycs, mt-Cytb, Gm10053, Cacna1d, Tubb4b, Ppp3cc, Lrp1, Itpr3, Psmd2, Psmb5, Cox5a, Frat2, Plcb2, Psma5, Gm12989, Psmd3, Psma7, Tubb5, Gm3375, Map2k1, Calm1, Gm8394, Adrm1, Psma6, Psmb6, Vdac3-ps1, Calm3, Psmc2, Atf4, Casp9, Psmc1, Psmb7, Vdac3, Ulk2, Psmd7, Cyc1, Atf6, Casp7, Kif5c, Atp2a3, Psma3, Psmc5, Psmb3, Psma4, Eif2s1, Psma2, Frat1, Bid, Nfkb1, 4930481A15Rik, Psmc4, Uqcrq, AC114990.3, Ppp3ca, Gm13835, Slc25a5, Psmd14, Csnk1a1, Sdhb, Psmd12, Psmd4, Dvl2, Lrp6, Cox7a2, Map2k2, Psma1, Psmb2, Atp5g3, Xbp1, Psmc3, Fzd4, Psmb4, Rtn3, Csnk2b, Hsd17b10, Ndufb6, Psmd6, Ndufa11, Kif5a, Vdac2, Aph1b, Map3k5, Pik3r4, Atp5g1, Uqcrc2, Bad, Ndufb9, Cdk5r1, Psmd13, Ndufa5, Gm16418, Ndufa3, Gm6542, Lrp5, Pik3c3, Psmc6, mt-Nd2, Atp5a1, Cox7b, Ndufs6, Tubb4a, Cox6a1, Rtn4, Ndufc1, Uqcrb, Hras, Uqcrc1, Wipi1, Ndufa2, Gm7591, Tuba1b, mt-Nd4l, Cox8a, Atp5e, Gm5529, Gm9575, Atg14, Ulk1, Ppp3r1, Cox6b1, Adam10, Mapk9, Ppp3cb, Akt2, Wnt2b, Plcb4, Ddit3, Ndufs4, Psmb1, Gm4950, Atg13, Ndufb8, Ndufa9, Atp5f1, Ndufab1, Ndufa13, Ndufa10, Uqcr10, Sem1, Ndufab1-ps, mt-Nd5, Atp5d, Gm10039, Ndufv2, Ndufv1, Ndufs3, Tuba1c, Rb1cc1, Ndufb3, Ndufa1, Insr, Ndufb7, Ide, Tubb4b-ps1, Klc2, Atp5h, Ndufs7, Uqcr11, 4930447K03Rik, Pik3r2, Ndufa12, Ndufa8, Cox4i1, Gsk3b, Ndufs8, Cox6a2, Sdhd, Atp5j, Gpr83*
15	mmu05166	Human T-cell leukemia virus 1 infection-Mus musculus (mouse)	195	9.29E-06	15.14	*H2-M2, Tnf, H2-Q6, Nfkbia, Cdkn1a, Cd40, H2-Q7, Relb, Icam1, H2-Q5, Ets2, Nfkb2, H2-Q4, Il15ra, H2-T24, H2-T22, Ccnd1, Il2rg, H2-K1, Egr2, H2-Ab1, H2-T23, Itgal, H2-Aa, Slc2a1, Fos, Jun, Myc, Cdkn2b, H2-M3, Calr, H2-DMa, H2-Eb1, Cdkn2c, H2-T10, Ccna2, Nrp1, Gm9574, Rela, Map3k1, Il6, Tspo, Adcy3, Ppp3cc, E2f2, H2-DMb1, B2m, Spi1, Il2rb, Atf6b, Tgfbr2, Il15, Ets1, Map2k1, Gm11131, H2-D1, Vdac3-ps1, Lta, Atf4, Ccne1, Creb5, Vdac3, H2-Oa, Bub1b, Prkacb, Bax, Tgfb3, Nfkb1, Nfatc2, Canx, Ppp3ca, Slc25a5, Anapc5,*

						Pttg1, Nfatc1, Creb3l2, Map2k2, Cdc20, Adcy7, Bcl2l1, Vdac2, Adcy9, Smad2, H2-Q10, Chek1, Ranbp1, Cdk2, Ccnb2, Crtc2, Ltbr, Hras, Gm7591, Tgfbr1, Ranbp3, Ccnd2, Gm5529, Ppp3r1, Mapk9, Ppp3cb, Akt2, Ikbkg, Bub3, H2-Eb2, Rb1, Adcy2, Creb3, Zfp36, H2-Ob, Pik3r2, Trp53, Gm7030, Polb
16	mmu04659	Th17 cell differentiation-Mus musculus (mouse)	112	1.29E-05	8.70	*Nfkbie, Nfkbia, Il1b, Il21r, Jak2, Il2rg, Stat1, Hif1a, H2-Ab1, Hsp90ab1, H2-Aa, Nfkbib, Fos, Jun, Mapk14, H2-DMa, H2-Eb1, Rela, Il6, Il12rb1, Cd247, Ppp3cc, Ifngr2, H2-DMb1, Il2rb, Rora, Tgfbr2, Mapk11, Il23r, Hsp90aa1, Il6st, Ahr, H2-Oa, Irf4, Nfkb1, Nfatc2, Ppp3ca, Il6ra, Nfatc1, Plcg1, Il4ra, Runx1, Zhx3, Il4, Smad2, Rxra, Tgfbr1, Tyk2, Ppp3r1, Mapk9, Ppp3cb, Il1rap, Ikbkg, H2-Eb2, Tbx21, Stat3, Mapk12, H2-Ob*
17	mmu04625	C-type lectin receptor signaling pathway-Mus musculus (mouse)	58	1.58E-05	4.50	*Clec4e, Tnf, Nfkbia, Irf1, Mapkapk2, Src, Il1b, Ptgs2, Relb, Bcl3, Nfkb2, Clec7a, Ikbke, Malt1, Lsp1, Stat2, Stat1, Egr2, Irf9, Nlrp3, Jun, Clec4n, Mapk14, Ccl22, Rela, Il6, Il12b, Ppp3cc, Clec4b1, Mdm2, Itpr3, Il12a, Rras, Mapk11, Fcer1g, Calm1, Plk3, Calm3, Casp1, Clec4d, Syk, Prkcd, Nfkb1, Nfatc2, Ppp3ca, Nfatc1, Bcl10, Cblb, Pak1, Gm17334, Hras, Ppp3r1, Mapk9, Ppp3cb, Rhoa, Akt2, Ikbkg, Mapk12, Il10, Cyld, Pik3r2*
18	mmu05200	Pathways in cancer-Mus musculus (mouse)	61	1.58E-05	4.74	*Traf1, Ednrb, Rasgrp3, Nfkbia, Il7r, Mmp9, Cdkn1a, Birc3, Pparg, Ptgs2, Lpar6, Nfkb2, Rasgrp1, Cebpa, Itga6, Il15ra, Rac2, Pim1, Fas, Jak2, Nqo1, Ccnd1, Il2rg, Stat2, Cxcr4, Pdgfb, Txnrd1, Stat1, Vegfa, Ralgds, Hif1a, Gsta3, Gng12, Met, Hsp90ab1, Epas1, Traf2, Slc2a1, Gna12, Fos, Igf1, Jun, Cdk6, Edn1, Lpar1, Myc, Nos2, Cdkn2b, Fgfr1, Fzd1, Bcl2l11, Keap1, Bak1, Fzd7, Kitl, Pim2, Wnt6, Bmp2, Ccna2, Rela, Nfe2l2, Csf2rb, Hmox1, Il6, Il12rb1, Gng2, Il12b, Cycs, Gngt2, Max, Zbtb16, Gm10053, Adcy3, Ifngr2, E2f2, Pdgfrb, Mdm2, Il12a, Spi1, Il2rb, Gm7019, Hgf, Hdac1, Frat2, Mmp2, Plcb2, Rad51, Birc2, Skp2, Msh2, Gstm2, Tgfbr2, Il13ra1, Il15, Rassf5, Ets1, Map2k1, Brca2, Calm1, Il23r, Hsp90aa1, Cdkn1b, Calm3, Pml, Ccne1, Casp9, Il6st, Gadd45b, Gnb1, Lpar2, Traf5, Pdgfa, Prkacb, Casp7, Col4a2, Gstt1, Itgav, Ptger4, Lpar5, Cul1, Zbtb17, Bax, Tgfb3, Hsp90b1, Mgst3, Prkcb, Lama3, Lamb2, Frat1, Bid, Nfkb1, Dapk3, Birc5, Vegfc, Il6ra, Jup, Dvl2, Tcf7l2, Lrp6, Plcg1, Map2k2, Igf1r, Adcy7, Msh6, Fzd4, Rasgrp4,*

					Pgf, Il4ra, Bcl2l1, Gstm1, Foxo1, Runx1, Il7, Rala, Zhx3, Vhl, Adcy9, Il4, Itgb1, Smad2, Gnb2, Bad, Fn1, Elob, Lrp5, Rxra, Camk2b, Abl1, Ncoa3, Hey1, Cdk2, Col4a5, Cul2, Hras, Tgfbr1, Gm10093, Ctnna1, Ccnd2, Egln1, Ifnar2, Ptk2, Rbx1, Mapk9, Plekhg5, Rhoa, Akt2, Wnt2b, Plcb4, E230016M11Rik, Ptch1, Jag1, Prkcg, Ikbkg, Ncoa1, Egln2, Mlh1, Rb1, Cks1b, Polk, Csf3r, Adcy2, Stat3, Gstm4, Gng7, Gsto1, Gstt3, Pik3r2, Trp53, Mgst1, Gsk3b, Gnas, Gstp1
19	mmu04141	Protein processing in endoplasmic reticulum-Mus musculus (mouse)	212	1.63E-05	16.46	*Ppp1r15a, Capn2, Hsp90ab1, Traf2, Calr, Herpud1, Vcp, Bak1, Eif2ak2, Man1c1, Nfe2l2, Dnajc10, Dnaja1, Hspa5, Rrbp1, P4hb, Vcp-rs, Atf6b, Ckap4, Hspa8, Dnajb11, Pdia6, Rad23b, Hsp90aa1, Nploc4, Dnajc1, Atf4, Sec13, Wfs1, Atf6, Ssr2, Edem1, Hsph1, Mbtps2, Cul1, Eif2s1, Dnaja2, Bax, Hsp90b1, Edem2, Canx, Nsfl1c, Selenos, Ubxn8, Atxn3, Hspa1b, Rpn1, Prkcsh, Dnajb1, Derl2, Fbxo6, Xbp1, Sec23b, Sar1a, Man1a, Ddost, March6, Gm5555, Hyou1, Map3k5, Svip, Ubqln2, Hspa2, Pdia3, Bag1, Rad23a, Ube2d3, Sec61b, Ube2j2, Edem3, Ubqln1, Rpn2, Ube2j1, Plaa, Eif2ak4, Rbx1, Sar1b, Mapk9, Ubxn1, Ube2d-ps, Gm15542, Ddit3, Gm8355, Ube2d2a, Ube2g2, Derl1, Ssr4, Lman1, Ufd1, Sil1, Ero1l, Mogs, Os9, Pdia4, Dnajb2*
20	mmu05323	Rheumatoid arthritis-Mus musculus (mouse)	95	3.65E-05	7.38	*Tlr2, Tnf, Cxcl2, Ccl5, Cxcl3, Cd28, Atp6v0d2, Il1b, Icam1, Cxcl1, Atp6v0a1, Il1a, Acp5, Ccl3, Vegfa, H2-Ab1, Itgal, Csf1, H2-Aa, Fos, Jun, H2-DMa, H2-Eb1, Ccl2, Atp6v1b2, Il6, H2-DMb1, Il15, H2-Oa, Atp6v1e1, Tnfrsf11a, Tnfsf13, Tgfb3, Ltb, Atp6v1a, Cd86, Atp6v0b, Atp6v1f, Tlr4, Atp6v1g1, H2-Eb2, Atp6v0c-ps2, Mmp3, Atp6v0d1, Cd80, H2-Ob, Atp6v1d*
21	mmu04145	Phagosome-Mus musculus (mouse)	47	5.08E-05	3.65	*H2-M2, Marco, Tlr2, H2-Q6, C3, Tap1, Atp6v0d2, H2-Q7, Fcgr2b, Itgb5, H2-Q5, C1ra, Atp6v0a1, H2-Q4, Cd14, Clec7a, Ncf1, Cd36, H2-T24, H2-T22, Rab7b, H2-K1, H2-Ab1, H2-T23, M6pr, Cybb, Tap2, Mrc1, H2-Aa, Fcgr3, Colec12, Itgb3, H2-M3, Calr, Ncf4, H2-DMa, H2-Eb1, Scarb1, Tuba4a, H2-T10, Atp6v1b2, Gm9574, Msr1, Tlr6, Tubb4b, H2-DMb1, Coro1a, Tubb5, Gm11131, Tfrc, H2-D1, Fcgr1, Olr1, H2-Oa, Itga5, Atp6v1e1, Cyba, Itgav, Rab5c, Canx, Actb, Dync1li1, Atp6v1a, Vamp1, Gm38317, Sec22b, Atp6v0b, Atp6v1f, Itgb1, H2-Q10, Tlr4, Pik3c3, Sec61b, Tubb4a, Fcgr4, Pikfyve, Tuba1b, Atp6v1g1, H2-*

						Eb2, Atp6v0c-ps2, Atp6v0d1, Tuba1c, Thbs1, H2-Ob, Atp6v1d, Tubb4b-ps1, Comp, 4930447K03Rik, Gm7030, Stx12
22	mmu05140	Leishmaniasis-Mus musculus (mouse)	90	6.66E-05	6.99	*Tlr2, Tnf, Marcksl1, Nfkbia, C3, Il1b, Ptgs2, Il1a, Ncf1, Jak2, Stat1, H2-Ab1, Cybb, H2-Aa, Nfkbib, Fos, Fcgr3, Jun, Nos2, Ncf4, Mapk14, H2-DMa, H2-Eb1, Gm6548, Ptpn6, Myd88, Rela, Il12b, Ifngr2, H2-DMb1, Il12a, Mapk11, Fcgr1, H2-Oa, Cyba, Tgfb3, Prkcb, Nfkb1, Il4, Itgb1, Tlr4, Fcgr4, H2-Eb2, Mapk12, Il10, H2-Ob*
23	mmu04514	Cell adhesion molecules-Mus musculus (mouse)	46	8.27E-05	3.57	*H2-M2, Sdc1, H2-Q6, Cd40, Cd28, H2-Q7, Icam1, H2-Q5, Vsir, H2-Q4, Itga6, Cd274, Icosl, Nectin2, Spn, H2-T24, H2-T22, Cadm1, Itgb8, H2-K1, H2-Ab1, H2-T23, Itgal, Alcam, H2-Aa, Mag, Ntng2, H2-M3, Vcam1, H2-DMa, H2-Eb1, Sdc4, H2-T10, Gm9574, Lrrc4, L1cam, H2-DMb1, Gm11131, H2-D1, Cd276, Vcan, H2-Oa, Itgav, Ptprm, 6530402F18Rik, Itgb7, Pvr, Nrcam, Cd86, Mpzl1, Itgb1, Selplg, H2-Q10, Siglec1, Madcam1, Glg1, Ptprc, H2-Eb2, Cd80, Igsf11, H2-Ob, Gm7030*
24	mmu05150	Staphylococcus aureus infection-Mus musculus (mouse)	62	0.000101356	4.81	*Fpr2, Fpr1, C3, Fcgr2b, Icam1, C1ra, C3ar1, H2-Ab1, Cfh, Itgal, H2-Aa, Fcgr3, Cfb, H2-DMa, H2-Eb1, Camp, C1s1, Fpr3, H2-DMb1, Fcgr1, H2-Oa, C5ar1, C1qa, Selplg, Fcgr4, C2, H2-Eb2, Il10, H2-Ob*
25	mmu05203	Viral carcinogenesis-Mus musculus (mouse)	29	0.000140902	2.25	*H2-M2, Irf7, Traf1, H2-Q6, Nfkbia, C3, Cdkn1a, Mapkapk2, Atp6v0d2, Src, H2-Q7, H2-Q5, Nfkb2, H2-Q4, H2-T24, H2-T22, Ccnd1, Sp100, H2-K1, Egr2, Hdac5, H2-T23, Irf9, Hdac9, Hpn, Traf2, Jun, Cdk6, Cdkn2b, H2-M3, Bak1, Eif2ak2, H2-T10, Ccna2, Gsn, Gm9574, Rela, Mdm2, Snd1, Hdac1, Pkm, Atf6b, Skp2, Rbpj, Ccr3, Gm11131, Hist2h2be, H2-D1, Vdac3-ps1, Cdkn1b, Atf4, Ccne1, Creb5, Psmc1, Il6st, Vdac3, Traf5, Prkacb, Gtf2e2, Syk, Bax, Ywhaz, Hdac10, Ywhah, Nfkb1, Mrps18b, Lyn, Ywhae, Creb3l2, Cdc20, Gtf2a1, H2-Q10, Bad, Chek1, Ranbp1, Cdk2, Gm6560, Ltbr, Hras, Hdac4, Gm7591, Gm10093, Ccnd2, Gm9575, Ddx3x, Actn1, Rhoa, Gm13552, Ikbkg, Gtf2h2, Rb1, Atp6v0d1, Creb3, Stat3, Actn4, Pik3r2, Trp53, Pxn, Ywhab, Gm7030, Hist3h2ba, Polb*

26	mmu05416	Viral myocarditis-Mus musculus (mouse)	102	0.000140902	7.92	*H2-M2, H2-Q6, Cd40, Cd28, H2-Q7, Icam1, H2-Q5, H2-Q4, Rac2, H2-T24, H2-T22, Ccnd1, Fyn, Cav1, Cd55, H2-K1, H2-Ab1, H2-T23, Itgal, H2-Aa, Eif4g2, H2-M3, H2-DMa, H2-Eb1, H2-T10, Gm9574, Cycs, Gm10053, H2-DMb1, Gm11131, H2-D1, Casp9, H2-Oa, Bid, Dag1, Actb, Sgcb, Cd86, H2-Q10, Abl1, H2-Eb2, Cd80, H2-Ob, Gm7030*
27	mmu04612	Antigen processing and presentation-Mus musculus (mouse)	44	0.000140902	3.42	*H2-M2, Tnf, H2-Q6, Tapbp, Tap1, H2-Q7, H2-Q5, H2-Q4, H2-T24, H2-T22, H2-K1, Psme2, H2-Ab1, H2-T23, Psme2b, Tap2, Hsp90ab1, H2-Aa, H2-M3, Calr, H2-DMa, H2-Eb1, Psme1, H2-T10, Gm9574, Hspa5, H2-DMb1, B2m, Lgmn, Ciita, Cd74, Hspa8, Gm11131, Hsp90aa1, H2-D1, H2-Oa, Canx, Hspa1b, Ctsb, H2-Q10, Hspa2, Hspa4, Pdia3, Gm8355, Rfx5, H2-Eb2, H2-Ob, Gm7030*
28	mmu04060	Cytokine-cytokine receptor interaction-Mus musculus (mouse)	48	0.000189153	3.73	*Tnf, Cxcl2, Ccl5, Il7r, Cxcl10, Cd40, Cxcl3, Il1b, Cxcl1, Il1rn, Il1a, Il21r, Il15ra, Fas, Ccl3, Il2rg, Il27, Cxcr4, Tnfsf9, Il1f9, Tnfsf10, Cxcr3, Csf1, Cxcl16, Ccr1, Cxcl9, Gdf15, Ccl9, Ccl4, Il16, Tnfrsf1b, Tnfsf4, Ccl22, Csf3, Ccl2, Bmp2, Lifr, Csf2rb, Il6, Il12rb1, Tnfrsf21, Il12b, Inhba, Tnfrsf8, Ifngr2, Pf4, Il12a, Il2rb, Il20rb, Ccl7, Tnfsf15, Stard5, Tnfrsf12a, Tgfbr2, Il13ra1, Il15, Relt, Ccr3, Il23r, Lta, Il6st, Tnfrsf14, Ccr7, Tnfrsf11a, Bmpr2, Tnfsf13, Tnfrsf9, Cxcl14, Tgfb3, Ltb, Acvrl1, Il6ra, Gdf3, Ccr2, Osm, Il4ra, Xcl1, Il7, Ccl6, Il4, Ltbr, Tnfsf12, Tgfbr1, Ifnar2, Il1rap, Ccl27a, Lif, Acvr1b, Tnfrsf13b, Csf3r, Il10, Crlf2*
29	mmu04218	Cellular senescence-Mus musculus (mouse)	92	0.000240041	7.14	*H2-M2, H2-Q6, Cdkn1a, Mapkapk2, H2-Q7, H2-Q5, Sqstm1, Il1a, H2-Q4, H2-T24, H2-T22, Ccnd1, H2-K1, Capn2, H2-T23, Cdk6, Zfp36l1, Myc, Cdkn2b, H2-M3, Mapk14, H2-T10, Ccna2, Gm9574, Rela, Il6, Cacna1d, Ppp3cc, E2f2, Mdm2, Foxo3, Itpr3, Ppp1cb, Rras, Tgfbr2, Rassf5, Ets1, Mapk11, Map2k1, Calm1, Gm11131, H2-D1, Vdac3-ps1, Calm3, Ccne1, Vdac3, Gadd45b, Map2k6, Trpv4, Foxm1, Tgfb3, Cdc25a, Nfkb1, Nfatc2, Mybl2, Ppp3ca, Sirt1, Slc25a5, Nfatc1, Map2k2, Zfp36l2, Serpine1, Foxo1, Vdac2, Map2k3, Smad2, H2-Q10, Chek1, Cdk2, Ppp1ca, Ccnb2, Gm12188, Hras, Gm7591, Tgfbr1, Ccnd2, Gm5529, Ppp3r1, Ppp3cb, Akt2, E230016M11Rik, Rad50, E2f4, Eif4ebp1, Rb1, Tsc1, Mapk12, Pik3r2, Trp53, Gm7030*

30	mmu05162	Measles-Mus musculus (mouse)	90	0.000380204	6.99	*Irf7, Tlr2, Mx1, Nfkbia, Cd28, Il1b, Fcgr2b, Ifih1, Ddx58, Mx2, Oas1g, Il1a, Oas2, Oas3, Oas1a, Fas, Ikbke, Tnfaip3, Ccnd1, Il2rg, Stat2, Stat1, Oas1b, Irf9, Nfkbib, Fos, Jun, Cdk6, Bak1, Eif2ak2, Myd88, Rela, Il6, Il12b, Cycs, Gm10053, Il12a, Il2rb, Adar, Hspa8, Slamf1, Cdkn1b, Tbk1, Ccne1, Casp9, Eif2s1, Bax, Bid, Nfkb1, Hspa1b, Csnk2b, Bcl2l1, Cblb, Bad, Hspa2, Tlr4, Rchy1, Cdk2, Tyk2, Ccnd2, Ifnar2, Eif2ak4, Mapk9, Akt2, Gm8355, Ikbkg, Stat3, Msn, Pik3r2, Trp53, Gsk3b*
31	mmu05170	Human immunodeficiency virus 1 infection-Mus musculus (mouse)	71	0.000380204	5.51	*H2-M2, Tlr2, Tnf, H2-Q6, Nfkbia, Tapbp, Tap1, H2-Q7, H2-Q5, Apobec3, H2-Q4, Trim30d, Rac2, Fas, Samhd1, H2-T24, H2-T22, Cxcr4, H2-K1, Bst2, H2-T23, Gng12, Tap2, Ap1s2, Traf2, Fos, Jun, H2-M3, Calr, Mapk14, Wee1, Tnfrsf1b, Bak1, Ap1b1, Trim12c, H2-T10, Myd88, Gm9574, Rela, Cd247, Gng2, Cycs, Gngt2, Gm10053, Ppp3cc, Itpr3, B2m, Trim5, Mapk11, Map2k1, Calm1, Gm11131, H2-D1, Calm3, Mb21d1, Tbk1, Casp9, Gnb1, Traf5, Map2k6, Cul1, Cfl1, Bax, Prkcb, Bid, Nfkb1, Nfatc2, Ppp3ca, Nfatc1, Plcg1, Map2k2, Ap1s1, Bcl2l1, Zhx3, Map2k3, H2-Q10, Gnb2, Bad, Pak1, Chek1, Tlr4, Elob, Pdia3, Ccnb2, Hras, Ppp3r1, Ptk2, Rbx1, Mapk9, Ppp3cb, Akt2, Prkcg, Ikbkg, Tmem173, Pak4, Mapk12, Gng7, Pik3r2, Pxn, Gm7030*
32	mmu04658	Th1 and Th2 cell differentiation-Mus musculus (mouse)	100	0.000385196	7.77	*Nfkbie, Nfkbia, Jak2, Il2rg, Stat1, H2-Ab1, H2-Aa, Nfkbib, Fos, Jun, Mapk14, H2-DMa, H2-Eb1, Rela, Il12rb1, Cd247, Il12b, Ppp3cc, Ifngr2, H2-DMb1, Il12a, Il2rb, Mapk11, Rbpj, Maml3, H2-Oa, Nfkb1, Nfatc2, Ppp3ca, Nfatc1, Plcg1, Il4ra, Zhx3, Il4, Maf, Tyk2, Maml2, Ppp3r1, Mapk9, Ppp3cb, Jag1, Ikbkg, H2-Eb2, Tbx21, Mapk12, H2-Ob*
33	mmu05202	Transcriptional misregulation in cancer-Mus musculus (mouse)	46	0.000460702	3.57	*Traf1, Mmp9, Cdkn1a, Cd40, Birc3, Pparg, Nfkbiz, Cebpa, Cd14, Nupr1, Bcl2a1a, Mef2c, Hpgd, Bcl2a1d, Met, Igf1, Eya1, Myc, Etv5, Bak1, Fli1, Cebpb, Plau, Cdkn2c, Ccna2, Rela, Il6, Max, Zbtb16, Hhex, Mdm2, Spi1, Il2rb, Gm7019, Hdac1, Jmjd1c, Birc2, Tgfbr2, Cdkn1b, Pml, Nsd2, Cdk14, Fcgr1, Gadd45b, Pbx1, Bmi1, Pdgfa, Dot1l, Six1, Zbtb17, Uty, Bax, Nfkb1, H3f3a, Fus, Klf3, Jup, Gzmb, Runx2, Bmp2k, Igf1r, Itgb7, Bcl2l1, Foxo1, Cd86, Runx1, Lmo2, Meis1, Rxra, Maf, Etv6, Zeb1, Gm10093, Bcl2a1b, Ccnd2, Taf15, Ptk2, Ewsr1,*

						E230016M11Rik, Ddit3, Bcl6, Dusp6, Mmp3, Polk, Gm12657, Ldb1, H3f3a-ps1, Aspscr1, Trp53
34	mmu04064	NF-kappa B signaling pathway-Mus musculus (mouse)	89	0.000498396	6.91	*Tnf, Traf1, Cxcl2, Nfkbia, Cd40, Cxcl3, Birc3, Il1b, Ptgs2, Relb, Icam1, Ddx58, Nfkb2, Cxcl1, Cd14, Bcl2a1a, Malt1, Tnfaip3, Bcl2a1d, Cflar, Traf2, Card11, Ccl4, Vcam1, Plau, Myd88, Rela, Ticam2, Gm7019, Birc2, Lta, Gadd45b, Traf5, Tnfrsf11a, Syk, Ltb, Prkcb, Nfkb1, Card14, Lyn, Pias4, Plcg1, Edaradd, Csnk2b, Bcl2l1, Bcl10, Zhx3, Tlr4, Ltbr, Bcl2a1b, E230016M11Rik, Ikbkg, Cyld*
35	mmu05152	Tuberculosis-Mus musculus (mouse)	53	0.000678166	4.12	*Clec4e, Tlr2, Tnf, C3, Atp6v0d2, Src, Il1b, Fcgr2b, Tlr1, Atp6v0a1, Il1a, Cd14, Clec7a, Jak2, Malt1, Lsp1, Ripk2, Stat1, H2-Ab1, Mrc1, H2-Aa, Nod2, Irak2, Fcgr3, Nos2, Mapk14, H2-DMa, H2-Eb1, Cebpb, Camp, Myd88, Rela, Il6, Il12b, Cycs, Tlr6, Gm10053, Ppp3cc, Ifngr2, H2-DMb1, Itgax, Il12a, Coro1a, Ciita, Cd74, Mapk11, Fcer1g, Calm1, Plk3, Hspa9, Calm3, Casp9, Fcgr1, H2-Oa, Rab5c, Syk, Bax, Tgfb3, Bid, Nfkb1, Ppp3ca, Hspd1, Bcl10, Atp6v0b, Bad, Tlr4, Pik3c3, Camk2b, Fcgr4, Ppp3r1, Mapk9, Ppp3cb, Rhoa, Akt2, Rfx5, H2-Eb2, Atp6v0c-ps2, Atp6v0d1, Mapk12, Il10, H2-Ob*
36	mmu05145	Toxoplasmosis-Mus musculus (mouse)	81	0.000861527	6.29	*Tlr2, Tnf, Nfkbia, Cd40, Birc3, Igtp, Itga6, Irgm2, Jak2, Pik3r6, Stat1, Socs1, H2-Ab1, H2-Aa, Nfkbib, Nos2, Mapk14, H2-DMa, H2-Eb1, Myd88, Rela, Il12b, Cycs, Gm10053, Ifngr2, H2-DMb1, Il12a, Gm7019, Ciita, Birc2, Hspa8, Mapk11, Casp9, H2-Oa, Map2k6, Tgfb3, Lama3, Lamb2, Nfkb1, Hspa1b, Bcl2l1, Map2k3, Itgb1, Bad, Hspa2, Tlr4, Tyk2, Mapk9, Akt2, Gm8355, Ikbkg, H2-Eb2, Pik3r5, Stat3, Mapk12, Il10, H2-Ob*
37	mmu05016	Huntington disease-Mus musculus (mouse)	57	0.000943036	4.43	*Sod2, Pparg, Ift57, Hip1, Traf2, Klc4, Gpx3, Psmd11, Tuba4a, Atp5b, Cycs, mt-Cytb, Gm10053, Tubb4b, Psmd2, Psmb5, Hdac1, Cox5a, Plcb2, Psma5, Gm12989, Psmd3, Psma7, Tubb5, Gm3375, Gm8394, Adrm1, Psma6, Psmb6, Vdac3-ps1, Psmc2, Casp9, Creb5, Psmc1, Psmb7, Vdac3, Ulk2, Psmd7, Cyc1, Kif5c, Psma3, Cacna1b, Psmc5, Psmb3, Psma4, Bax, Psma2, 4930481A15Rik, Psmc4, Uqcrq, AC114990.3, Gm13835, Slc25a5, Polr2g, Psmd14, Sdhb, Rest, Psmd12, Psmd4, Cox7a2, Creb3l2, Psma1, Psmb2, Atp5g3, Psmc3, Psmb4,*

						Dnah17, Hap1, Ndufb6, Psmd6, Ndufa11, Kif5a, Actr1a, Vdac2, Map3k5, Pik3r4, Atp5g1, Uqcrc2, Ndufb9, Psmd13, Ndufa5, Gm16418, Ndufa3, Gm6542, Pik3c3, Psmc6, mt-Nd2, Atp5a1, Cox7b, Ndufs6, Tubb4a, Cox6a1, Ndufc1, Uqcrb, Uqcrc1, Wipi1, Ndufa2, Gm7591, Tuba1b, mt-Nd4l, Gm10093, Polr2c, Cox8a, Atp5e, Gm5529, Gm9575, Atg14, Ulk1, Polr2l, Cox6b1, Mapk9, Plcb4, Ndufs4, Psmb1, Gm4950, Slc1a2, Atg13, Ndufb8, Ndufa9, Actr1b, Ap2a2, Polr2k, Actr10, Atp5f1, Ndufab1, Ndufa13, Ndufa10, Uqcr10, Tgm2, Sem1, Ndufab1-ps, mt-Nd5, Atp5d, Gm10039, Ndufv2, Taf4, Ndufv1, Gm20560, Creb3, Ndufs3, Tuba1c, Rb1cc1, Ndufb3, Tfam, Polr2i, Ndufa1, Ndufb7, Dctn3, Tubb4b-ps1, Klc2, Lyrm1, Atp5h, Ndufs7, Uqcr11, 4930447K03Rik, Trp53, Rcor1, Ndufa12, Ndufa8, Cox4i1, Polr2f, Ndufs8, Cox6a2, Sdhd, Atp5j
38	mmu04932	Non-alcoholic fatty liver disease-Mus musculus (mouse)	165	0.001380995	12.81	*Tnf, Socs3, Il1b, Il1a, Cebpa, Fas, Nr1h3, Traf2, Irs2, Fos, Jun, Bcl2l11, Prkag2, Rela, Il6, Cycs, mt-Cytb, Gm10053, Cox5a, Atf4, Cyc1, Casp7, Adipor1, Eif2s1, Bax, Bid, Nfkb1, Mlx, 4930481A15Rik, Uqcrq, Sdhb, Il6ra, Cox7a2, Xbp1, Ndufb6, Ndufa11, Map3k5, Uqcrc2, Ndufb9, Ndufa5, Gm16418, Ndufa3, Rxra, Cox7b, Ndufs6, Cox6a1, Ndufc1, Uqcrb, Prkab2, Uqcrc1, Ndufa2, Cox8a, Cox6b1, Mapk9, Akt2, Ddit3, Ndufs4, Ndufb8, Ndufa9, Ndufab1, Ndufa13, Ndufa10, Uqcr10, Ndufab1-ps, Ndufv2, Ndufv1, Ndufs3, Ndufb3, Ndufa1, Insr, Ndufb7, Ndufs7, Uqcr11, Pik3r2, Ndufa12, Ndufa8, Cox4i1, Gsk3b, Ndufs8, Cox6a2, Sdhd, Adipor2*
39	mmu04010	MAPK signaling pathway-Mus musculus (mouse)	82	0.001401216	6.37	*Tnf, Rasgrp3, Mapkapk2, Il1b, Relb, Nfkb2, Il1a, Rasgrp1, Cd14, Daxx, Rac2, Fas, Mef2c, Pdgfb, Vegfa, Gng12, Dusp3, Met, Rasgrf1, Csf1, Traf2, Gna12, Fos, Nr4a1, Igf1, Jun, Myc, Pla2g4a, Mapk14, Fgfr1, Cacna1a, Kitl, Dusp7, Myd88, Rela, Map3k1, Cdc25b, Max, Mknk2, Cacna1d, Rps6ka4, Ppp3cc, Pdgfc, Pdgfrb, Dusp16, Hgf, Map3k12, Map3k20, Rps6ka3, Efna2, Cacng8, Rras, Tgfbr2, Cacnb1, Dusp1, Rapgef2, Dusp9, Hspa8, Mapk11, Map2k1, Stmn1, Map4k3, Atf4, Gadd45b, Pdgfa, Prkacb, Cacna1b, Rap1b, Rps6ka2, Map2k6, Tgfb3, Prkcb, Nfkb1, Ppp3ca, Cacnb3, Hspa1b, Vegfc, Nfatc1, Map2k2, Igf1r,*

						Rasa1, Dusp8, Rasgrp4, Taok3, Pgf, Map2k3, Map3k5, Map3k8, Pak1, Hspa2, Ppp5c, Hras, Tgfbr1, Ppm1b, Ppp3r1, Mapk9, Mknk1, Ppp3cb, Akt2, Il1rap, E230016M11Rik, Ddit3, Gm8355, Gm16183, Prkcg, Dusp2, Ikbkg, Gm11223, 1700029I15Rik, Dusp5, Dusp6, Kdr, Mapk12, Insr, Abhd15, Trp53, Angpt2
40	mmu04622	RIG-I-like receptor signaling pathway-Mus musculus (mouse)	117	0.001590608	9.09	*Irf7, Tnf, Dhx58, Nfkbia, Cxcl10, Ifih1, Isg15, Ddx58, Ikbke, Tank, Azi2, Nfkbib, Traf2, Mapk14, Otud5, Rela, Map3k1, Il12b, Il12a, Atg5, Mapk11, Tbk1, Nfkb1, Ddx3x, Mapk9, Ikbkg, Pin1, Tmem173, Mapk12, Tbkbp1, Cyld*
41	mmu05310	Asthma-Mus musculus (mouse)	31	0.001813254	2.41	*Tnf, Cd40, H2-Ab1, H2-Aa, H2-DMa, H2-Eb1, H2-DMb1, Fcer1g, H2-Oa, Il4, H2-Eb2, Il10, H2-Ob*
42	mmu04216	Ferroptosis-Mus musculus (mouse)	13	0.002024135	1.01	*Slc7a11, Acsl1, Slc40a1, Cp, Gclm, Gss, Acsl5, Steap3, Cybb, Prnp, Slc39a14, Hmox1, Slc39a8, Acsl4, Slc11a2, Atg5, Tfrc, Vdac3-ps1, Vdac3, Fth1, Atg7, Slc3a2, Pcbp1, Vdac2, Gm7591, Sat1, Acsl3, Gpx4-ps2, Trp53*
43	mmu05017	Spinocerebellar ataxia-Mus musculus (mouse)	29	0.002199131	2.25	*Traf2, Cacna1a, Psmd11, Cycs, Gm10053, Itpr3, Psmd2, Psmb5, Rora, Plcb2, Psma5, Gm12989, Psmd3, Psma7, Gm3375, Rbpj, Atxn1, Gm8394, Adrm1, Psma6, Psmb6, Vdac3-ps1, Psmc2, Psmc1, Psmb7, Vdac3, Ulk2, Psmd7, Atp2a3, Psma3, Psmc5, Psmb3, Psma4, Psma2, Prkcb, Psmc4, AC114990.3, Gm13835, Slc25a5, Atxn3, Psmd14, Psmd12, Psmd4, Psma1, Afg3l1, Psmb2, Xbp1, Psmc3, Psmb4, Psmd6, Vdac2, Map3k5, Pik3r4, Nop56, Psmd13, Gm6542, Pik3c3, Psmc6, Wipi1, Gm7591, Gm5529, Gm9575, Atg14, Ulk1, Mapk9, Akt2, Plcb4, Psmb1, Gm4950, Atg13, Gm16183, Prkcg, Sem1, Rb1cc1, Gm27219, Pik3r2*
44	mmu04066	HIF-1 signaling pathway-Mus musculus (mouse)	76	0.002199131	5.90	*Cdkn1a, Pfkl, Vegfa, Hif1a, Cybb, Pdk1, Slc2a1, Igf1, Edn1, Nos2, Rela, Hmox1, Il6, Mknk2, Ifngr2, Aldoa, Map2k1, Tfrc, Cdkn1b, Eif4e, Eno2, Prkcb, Nfkb1, Ldhb, Pgk1, Il6ra, Plcg1, Map2k2, Aldoc, Igf1r, Serpine1, Pfkp, Zhx3, Vhl, Tlr4, Elob, Eno1, Camk2b, Ltbr, Cul2, Gm12188, Egln1, Pdhb, Rbx1, Mknk1, Gm4735, Hk2, Akt2, AC122217.3, Prkcg, Eif4ebp1, Pfkfb3, Egln2, Stat3, Eif4e2, Insr, Pik3r2, Angpt2*
45	mmu05144	Malaria-Mus musculus	58	0.002780005	4.50	*Sdc1, Tlr2, Tnf, Cd40, Il1b, Icam1, Cd36, Itgal, Met, Vcam1, Csf3, Ccl2, Myd88,*

		(mouse)			*Il6, Lrp1, Il12a, Hgf, Gypc, Tgfb3, Cd81, Klrk1, Tlr4, Thbs1, Klrb1b, Il10, Comp*
46	mmu04061	Viral protein interaction with cytokine and cytokine receptor-Mus musculus (mouse)	26	0.003016266	2.02	*Tnf, Cxcl2, Ccl5, Cxcl10, Cxcl3, Cxcl1, Ccl3, Il2rg, Cxcr4, Tnfsf10, Cxcr3, Csf1, Ccr1, Cxcl9, Ccl9, Ccl4, Tnfrsf1b, Ccl22, Ccl2, Il6, Pf4, Il2rb, Il20rb, Ccl7, Ccr3, Lta, Il6st, Tnfrsf14, Ccr7, Cxcl14, Il6ra, Ccr2, Xcl1, Ccl6, Ltbr, Ccl27a, Il10*
47	mmu04660	T cell receptor signaling pathway-Mus musculus (mouse)	37	0.00341206	2.87	*Tnf, Nfkbie, Nfkbia, Cd28, Lcp2, Rasgrp1, Vav3, Malt1, Fyn, Nck1, Nfkbib, Fos, Jun, Card11, Mapk14, Tec, Vav1, Ptpn6, Rela, Nck2, Cd247, Ppp3cc, Mapk11, Map2k1, Nfkb1, Nfatc2, Ppp3ca, Nfatc1, Plcg1, Map2k2, Bcl10, Zhx3, Cblb, Il4, Map3k8, Pak1, Hras, Ppp3r1, Mapk9, Ppp3cb, Rhoa, Akt2, Ikbkg, Ptprc, Grap2, Pak4, Mapk12, Il10, Pik3r2, Gsk3b*
48	mmu04672	Intestinal immune network for IgA production-Mus musculus (mouse)	50	0.003509177	3.88	*Cd40, Cd28, Il15ra, Icosl, Cxcr4, H2-Ab1, H2-Aa, H2-DMa, H2-Eb1, Il6, H2-DMb1, Il15, H2-Oa, Tnfsf13, Itgb7, Cd86, Il4, Ltbr, Madcam1, Tnfrsf13b, H2-Eb2, Cd80, Il10, H2-Ob*
49	mmu04620	Toll-like receptor signaling pathway-Mus musculus (mouse)	24	0.003595628	1.86	*Irf7, Tlr2, Tnf, Ccl5, Nfkbia, Cxcl10, Cd40, Il1b, Tlr1, Cd14, Ikbke, Ccl3, Stat1, Fos, Cxcl9, Jun, Ccl4, Mapk14, Tlr3, Myd88, Rela, Il6, Il12b, Tlr6, Ticam2, Il12a, Mapk11, Map2k1, Tbk1, Map2k6, Spp1, Nfkb1, Tlr8, Map2k2, Cd86, Map2k3, Map3k8, Tlr4, Ifnar2, Mapk9, Akt2, Ikbkg, Cd80, Mapk12, Pik3r2*
50	mmu04062	Chemokine signaling pathway-Mus musculus (mouse)	45	0.003774071	3.49	*Cxcl2, Ccl5, Nfkbia, Cxcl10, Cxcl3, Src, Cxcl1, Hck, Vav3, Rac2, Ncf1, Ccl3, Jak2, Stat2, Cxcr4, Pik3r6, Stat1, Fgr, Gng12, Cxcr3, Cxcl16, Nfkbib, Ccr1, Cxcl9, Ccl9, Ccl4, Vav1, Ccl22, Ccl2, Rela, Gng2, Gngt2, Adcy3, Tiam1, Shc4, Foxo3, Pf4, Shc2, Ccl7, Plcb2, Map2k1, Ccr3, Gnb1, Ccr7, Prkacb, Cxcl14, Rap1b, Prkcd, Elmo1, Prkcb, Nfkb1, Lyn, Plcg1, Adcy7, Ccr2, Xcl1, Zhx3, Ccl6, Adcy9, Gnb2, Bad, Pak1, Dock2, Hras, Bcar1, Grk2, Ptk2, Rhoa, Akt2, Plcb4, Ccl27a, Ikbkg, Adcy2, Pik3r5, Stat3, Gng7, Pik3r2, Pxn, Gsk3b*
51	mmu04380	Osteoclast differentiation-Mus musculus (mouse)	79	0.003774071	6.13	*Tnf, Socs3, Nfkbia, Pparg, Il1b, Fcgr2b, Relb, Nfkb2, Lcp2, Sqstm1, Il1a, Acp5, Ncf1, Stat2, Fyn, Stat1, Socs1, Irf9, Csf1, Traf2, Fos, Fcgr3, Jun, Itgb3, Ncf4, Mapk14, Tec, Rela, Gm15931, Ppp3cc, Ifngr2, Spi1, Tgfbr2, Mapk11, Map2k1,*

						Fcgr1, Tnfrsf11a, Cyba, Syk, Map2k6, Nfkb1, Nfatc2, Ppp3ca, Trem2, Lilrb4a, Nfatc1, Sirpa, Pirb, Lilra6, Fcgr4, Gm14548, Tgfbr1, Tyk2, Ifnar2, Ppp3r1, Mapk9, Ppp3cb, Akt2, Ikbkg, Mapk12, Cyld, Pik3r2
52	mmu05164	Influenza A-Mus musculus (mouse)	62	0.00380682	4.81	*Irf7, Rsad2, Tnf, Ccl5, Mx1, Socs3, Nfkbia, Cxcl10, Il1b, Icam1, Ifih1, Ddx58, Mx2, Oas1g, Il1a, Oas2, Oas3, Oas1a, Fas, Ikbke, Jak2, Stat2, Stat1, H2-Ab1, Oas1b, Tnfsf10, Irf9, Nlrp3, H2-Aa, Nfkbib, Cdk6, Tlr3, H2-DMa, Bak1, H2-Eb1, Eif2ak2, Rnasel, Ccl2, Myd88, Rela, Il6, Il12b, Cycs, Gm10053, Ifngr2, H2-DMb1, Il12a, Ciita, Adar, Map2k1, Kpna2, Tbk1, Pml, Casp9, Tmprss4, H2-Oa, Casp1, Eif2s1, Bax, Prkcb, Bid, Nfkb1, Slc25a5, Actb, Dnajb1, Map2k2, Tlr4, Tyk2, Gm5529, Ifnar2, Akt2, Nxt1, Ikbkg, H2-Eb2, H2-Ob, Pik3r2, Gm48788*
53	mmu05014	Amyotrophic lateral sclerosis-Mus musculus (mouse)	77	0.003818234	5.98	*Tnf, Sqstm1, Daxx, Tank, Traf2, Pink1, Nos2, Klc4, Mapk14, Vcp, Tnfrsf1b, Gpx3, Psmd11, Tuba4a, Hspa5, Atp5b, Cycs, mt-Cytb, Gm10053, Tubb4b, Ppp3cc, Itpr3, Psmd2, Psmb5, Rab8a, Vcp-rs, Cox5a, Psma5, Gm12989, Psmd3, Psma7, Pfn1, Tubb5, Gm3375, Mapk11, Gm8394, Adrm1, Psma6, Psmb6, Psmc2, Tbk1, Atf4, Casp9, Sec13, Psmc1, Psmb7, Ulk2, Psmd7, Cyc1, Atf6, Kif5c, Psma3, Psmc5, Psmb3, Casp1, Psma4, Hnrnpa1, Map2k6, Ang, Eif2s1, Bax, Psma2, C9orf72, Bid, 4930481A15Rik, Psmc4, Uqcrq, AC114990.3, Ppp3ca, Gm13835, Optn, Fus, Psmd14, Sdhb, Psmd12, Psmd4, Actb, Cox7a2, Psma1, Psmb2, Atp5g3, Xbp1, Psmc3, Psmb4, Dnah17, Hap1, Bcl2l1, Gm2260, Ndufb6, Gm10052, Psmd6, Ndufa11, Kif5a, Actr1a, Map2k3, Map3k5, Tomm40, Pik3r4, Atp5g1, Uqcrc2, Nup62, Srsf7, Ubqln2, Bad, Ndufb9, Psmd13, Ndufa5, Gm16418, Nup54, Ndufa3, Gm6542, Pik3c3, Rab1a, Psmc6, mt-Nd2, Ubqln1, Atp5a1, Cox7b, Ndufs6, Tubb4a, Cox6a1, Ndufc1, Uqcrb, Uqcrc1, Wipi1, Ndufa2, Nup88, Tuba1b, mt-Nd4l, Cox8a, Atp5e, Gm9575, Srsf3, Atg14, Ulk1, Ppp3r1, Cox6b1, Ppp3cb, Chchd10, Gm6793, Ddit3, Ndufs4, Psmb1, Gm4950, Slc1a2, Nxt1, Atg13, Ndufb8, Ndufa9, Actr1b, Anxa11, Actr10, Seh1l, Atp5f1, Ndufab1, Ndufa13, Derl1, Ndufa10, Uqcr10, Sem1, Ndufab1-ps, mt-Nd5, Atp5d, Gm10039, Ndufv2, Ndufv1, Gm20560, Wdr41, Ndufs3, Tuba1c, Fig4, Rb1cc1, Ndufb3, Mapk12, Ndufa1, Ndufb7, Gm9761, Dctn3, Tubb4b-ps1,*

						Klc2, Lyrm1, Atp5h, Ndufs7, Uqcr11, 4930447K03Rik, Trp53, Ndufa12, Ndufa8, Cox4i1, Ndufs8, Cox6a2, Sdhd, Atp5j
54	mmu05160	Hepatitis C-Mus musculus (mouse)	193	0.003827733	14.99	*Irf7, Ifit1, Rsad2, Tnf, Mx1, Socs3, Nfkbia, Cdkn1a, Cxcl10, Ddx58, Mx2, Oas1g, Oas2, Oas3, Oas1a, Fas, Ikbke, Nr1h3, Ccnd1, Stat2, Stat1, Ifit1bl1, Oas1b, Irf9, Cflar, Traf2, Cdk6, Myc, Tlr3, Bak1, Scarb1, Eif2ak2, Rnasel, Rela, Cycs, Gm10053, E2f2, Map2k1, Tbk1, Casp9, Ppp2r2a, Eif2s1, Bax, Ywhaz, Ywhah, Bid, Nfkb1, Cd81, Ywhae, Ifit1bl2, Map2k2, Ppp2cb, Ppp2ca, Pias1, Bad, Rxra, Cdk2, Hras, Tyk2, Ifnar2, Eif2ak4, Akt2, Ikbkg, Ppp2r2d, Rb1, Stat3, Pik3r2, Trp53, Ywhab, Gsk3b*
55	mmu05206	MicroRNAs in cancer-Mus musculus (mouse)	70	0.003894502	5.44	*Mmp9, Cdkn1a, Ptgs2, Fscn1, Pim1, Kif23, Ccnd1, Pdgfb, Vegfa, Socs1, Hdac5, Met, Ezr, Irs2, Cdk6, Myc, Itgb3, Bcl2l11, Bak1, Plau, Abcb1a, Sox4, Hmox1, Cdc25b, Abcb1b, Pdcd4, E2f2, Shc4, Pdgfrb, Mdm2, Spry2, Dnmt3a, Hdac1, Efna2, Map2k1, Stmn1, Cdkn1b, Ccne1, Bmf, Bmi1, Pdgfa, Itga5, Bmpr2, Prkcb, Cdc25a, Nfkb1, Brca1, St14, Sirt1, Abcc1, Plcg1, Map2k2, Dnmt1, Zhx3, Abl1, Zeb1, Hras, Hdac4, Gm10093, Ccnd2, Rhoa, Dnmt3b, Prkcg, Slc7a1, Gm11223, Marcks, Ccng1, Foxp1, Rdx, Stat3, Pak4, Thbs1, Cdca5, Pik3r2, Trp53*
56	mmu05133	Pertussis-Mus musculus (mouse)	75	0.004679437	5.82	*Tnf, C3, Irf1, Il1b, C1ra, Il1a, Cd14, Nlrp3, Nod1, Fos, Jun, Nos2, Mapk14, Myd88, Rela, Irf8, Il6, C1s1, Il12b, Ticam2, Il12a, Mapk11, Calm1, Calm3, Itga5, Casp7, Casp1, Cfl1, Nfkb1, C1qa, Itgb1, Tlr4, Gm17334, C2, Mapk9, Rhoa, Mapk12, Il10*
57	mmu05165	Human papillomavirus infection-Mus musculus (mouse)	38	0.004819548	2.95	*H2-M2, Oasl1, Tnf, Mx1, H2-Q6, Oasl2, Cdkn1a, Irf1, Atp6v0d2, Ptgs2, H2-Q7, Itgb5, H2-Q5, Isg15, Mx2, Atp6v0a1, H2-Q4, Itga6, Fas, Ikbke, H2-T24, H2-T22, Ccnd1, Itgb8, Stat2, Stat1, Vegfa, H2-K1, H2-T23, Irf9, Cdk6, Itgb3, H2-M3, Fzd1, Tlr3, Bak1, Fzd7, Eif2ak2, H2-T10, Wnt6, Atp6v1b2, Ccna2, Gm9574, Rela, Lfng, Vwf, Pdgfrb, Mdm2, Hdac1, Tubg1, Pkm, Map2k1, Rbpj, Gm11131, Maml3, H2-D1, Cdkn1b, Tbk1, Ccne1, Creb5, Psmc1, Slc9a3r1, Itga5, Atp6v1e1, Prkacb, Col4a2, Itgav, Ptger4, Ppp2r2a, Spp1, Bax, Lama3, Lamb2, Nfkb1, Csnk1a1, Dvl2, Tcf7l2, Creb3l2, Map2k2, Fzd4, Itgb7, Ppp2cb, Ppp2ca,*

					Atp6v1a, Mpp5, Ppp2r5a, Gm38317, Foxo1, Atp6v0b, Gm2446, Atp6v1f, Itgb1, H2-Q10, Bad, Fn1, Hey1, Cdk2, Gm6560, Col4a5, Gm12188, Gm17334, Hras, Dlg3, Tyk2, Gm10093, Maml2, Ccnd2, Gm9575, Ifnar2, Ppp2r5c, Ptk2, Akt2, Wnt2b, Jag1, Eif4ebp1, Ikbkg, Atp6v1g1, Ppp2r2d, Rfng, Atp6v0c-ps2, Rb1, Tsc1, Atp6v0d1, Creb3, Thbs1, Atp6v1d, Comp, Prkci, Pik3r2, Trp53, Pxn, Gsk3b, Gm7030, Gnas, Itga1
58	mmu05418	Fluid shear stress and atherosclerosis-Mus musculus (mouse)	135	0.00619419	10.48	*Sdc1, Tnf, Ass1, Mmp9, Src, Il1b, Icam1, Sqstm1, Il1a, Gm5424, Rac2, Ncf1, Mef2c, Nqo1, Pdgfb, Cav1, Vegfa, Gsta3, Hsp90ab1, Fos, Jun, Edn1, Itgb3, Vcam1, Mapk14, Keap1, Thbd, Sdc4, Ccl2, Rela, Nfe2l2, Hmox1, Txn1, Mmp2, Gstm2, Dusp1, Mapk11, Calm1, Hsp90aa1, Calm3, Pdgfa, Bmpr2, Gstt1, Cyba, Itgav, Map2k6, Trpv4, Hsp90b1, Mgst3, Nfkb1, Sumo3, Mef2a, Pias4, Actb, Gstm1, Map3k5, Sumo1, Gpc1, Ptk2, Mapk9, Rhoa, Akt2, Ikbkg, Gm10241, Cav2, Kdr, Mapk12, Gstm4, Gsto1, Gstt3, Pik3r2, Trp53, Mgst1, Gstp1*
59	mmu05161	Hepatitis B-Mus musculus (mouse)	74	0.00619419	5.75	*Irf7, Tlr2, Tnf, Nfkbia, Mmp9, Cdkn1a, Src, Ifih1, Ddx58, Fas, Ikbke, Jak2, Stat2, Stat1, Egr2, Fos, Jun, Myc, Mapk14, Tlr3, Pcna, Myd88, Ccna2, Rela, Map3k1, Il6, Cycs, Gm10053, E2f2, Ticam2, Atf6b, Tgfbr2, Mapk11, Map2k1, Vdac3-ps1, Tbk1, Atf4, Ccne1, Casp9, Creb5, Vdac3, Map2k6, Bax, Ywhaz, Tgfb3, Prkcb, Bid, Nfkb1, Nfatc2, Birc5, Nfatc1, Creb3l2, Map2k2, Map2k3, Bad, Tlr4, Pcna-ps2, Cdk2, Hras, Gm7591, Tgfbr1, Tyk2, Ddx3x, Mapk9, Akt2, Prkcg, Ikbkg, Rb1, Creb3, Stat3, Mapk12, Pik3r2, Trp53, Ywhab*
60	mmu04211	Longevity regulating pathway-Mus musculus (mouse)	74	0.007418734	5.75	*Sod2, Pparg, Sesn1, Irs2, Igf1, Prkag2, Rela, Adcy3, Foxo3, Atf6b, Atg5, Atf4, Creb5, Prkacb, Eif4e, Adipor1, Bax, Nfkb1, Sirt1, Creb3l2, Igf1r, Adcy7, Foxo1, Adcy9, Stk11, Prkab2, Gm12188, Hras, Ehmt2, Ulk1, Akt2, Atg13, Eif4ebp1, Tsc1, Sesn2, Adcy2, Creb3, Rb1cc1, Eif4e2, Insr, Pik3r2, Trp53, Adipor2*
61	mmu04933	AGE-RAGE signaling pathway in diabetic complications-Mus musculus (mouse)	43	0.007418734	3.34	*Tnf, Il1b, Icam1, Il1a, Pim1, Jak2, Ccnd1, Stat1, Vegfa, Cybb, Jun, Edn1, Vcam1, Mapk14, Thbd, Ccl2, Rela, Il6, Mmp2, Plcb2, Tgfbr2, Mapk11, Cdkn1b, Col4a2, Prkcd, Bax, Tgfb3, Prkcb, Nfkb1, Vegfc, Nfatc1, Plcg1, Serpine1, Foxo1, Zhx3, Smad2, Fn1, Col4a5, Hras, Tgfbr1, Mapk9, Akt2, Plcb4, Stat3, Mapk12, Plcd1,*

						Pik3r2
62	mmu05222	Small cell lung cancer-Mus musculus (mouse)	47	0.012002408	3.65	*Traf1, Nfkbia, Cdkn1a, Birc3, Ptgs2, Itga6, Ccnd1, Traf2, Cdk6, Myc, Nos2, Cdkn2b, Bak1, Rela, Cycs, Max, Gm10053, E2f2, Gm7019, Birc2, Skp2, Cdkn1b, Ccne1, Casp9, Gadd45b, Traf5, Col4a2, Itgav, Zbtb17, Bax, Lama3, Lamb2, Nfkb1, Bcl2l1, Itgb1, Fn1, Rxra, Cdk2, Col4a5, Ptk2, Akt2, E230016M11Rik, Ikbkg, Rb1, Cks1b, Polk, Pik3r2, Trp53*
63	mmu04110	Cell cycle-Mus musculus (mouse)	48	0.012974115	3.73	*Cdkn1a, Ccnd1, Mcm7, Cdk6, Mcm2, Myc, Mcm6, Cdkn2b, Wee1, Cdkn2c, Pcna, Mcm5, Ccna2, Cdc25b, E2f2, Mdm2, Hdac1, Skp2, Plk1, Tfdp2, Cdkn1b, Ccne1, Gadd45b, Fzr1, Bub1b, Cdc6, Cul1, Zbtb17, Ywhaz, Tgfb3, Ywhah, Cdc25a, Mcm4, Anapc5, Pttg1, Ywhae, Ttk, Cdc20, Cdc14b, Mad2l2, Cdk7, Smad2, Chek1, Pcna-ps2, Abl1, Cdk2, Ccnb2, Gm10093, Ccnd2, Mcm3, Cdc14a, Rbx1, Cdc7, E230016M11Rik, Orc1, E2f4, Cdc45, Bub1, Bub3, Smc1a, Rb1, Stag2, Rad21, Trp53, Ywhab, Gsk3b*
64	mmu04151	PI3K-Akt signaling pathway-Mus musculus (mouse)	66	0.012974115	5.13	*Tlr2, Il7r, Cdkn1a, Itgb5, Lpar6, Itga6, Sgk1, Jak2, Ccnd1, Il2rg, Itgb8, Pdgfb, Pik3r6, Vegfa, Gng12, Met, Hsp90ab1, Csf1, Nr4a1, Igf1, Cdk6, Lpar1, Myc, Itgb3, Fgfr1, Bcl2l11, Kitl, Csf3, Rela, Il6, Gng2, Gngt2, Pdgfc, Vwf, Pdgfrb, Mdm2, Pck2, Foxo3, Il2rb, Hgf, Efna2, Atf6b, Map2k1, Hsp90aa1, Cdkn1b, Atf4, Ccne1, Casp9, Creb5, Gnb1, Lpar2, Pdgfa, Itga5, Col4a2, Eif4e, Itgav, Lpar5, Ppp2r2a, Syk, Spp1, Ywhaz, Hsp90b1, Ywhah, Lama3, Lamb2, Nfkb1, Brca1, Eif4b, Vegfc, Il6ra, Ywhae, Cdc37, Creb3l2, Myb, Map2k2, Igf1r, Itgb7, Ppp2cb, Ppp2ca, Osm, Pgf, Il4ra, Ppp2r5a, Bcl2l1, Gm38317, Il7, Gm2446, Il4, Itgb1, Gnb2, Bad, Sgk3, Fn1, Tlr4, Rxra, Cdk2, Crtc2, Col4a5, Stk11, Gm12188, Hras, Ccnd2, Ifnar2, Ppp2r5c, Ptk2, Akt2, Eif4ebp1, Ikbkg, Ppp2r2d, Tsc1, Csf3r, Creb3, Pik3r5, Kdr, Eif4e2, Insr, Thbs1, Gng7, Mtcp1, Comp, Pik3r2, Trp53, Ywhab, Gsk3b, Itga1, Angpt2*
65	mmu04657	IL-17 signaling pathway-Mus musculus (mouse)	126	0.014294628	9.78	*Tnf, Cxcl2, Nfkbia, Mmp9, Cxcl10, Cxcl3, Il1b, Ptgs2, Cxcl1, Lcn2, Ikbke, Tnfaip3, Hsp90ab1, Traf2, Fos, Jun, Mapk14, Cebpb, Csf3, Ccl2, Rela, Il6, S100a8, Ccl7, Mapk11, Hsp90aa1, Tbk1, Traf5, Hsp90b1, Nfkb1, Anapc5, Elavl1, Mmp13, Il4,*

						Usp25, Mapk9, Ikbkg, Mmp3, Mapk12, Srsf1, Gsk3b
66	mmu04662	B cell receptor signaling pathway-Mus musculus (mouse)	41	0.015422981	3.18	*Nfkbie, Rasgrp3, Nfkbia, Fcgr2b, Vav3, Rac2, Malt1, Nfkbib, Fos, Jun, Card11, Vav1, Ptpn6, Rela, Gm15931, Ppp3cc, Inppl1, Map2k1, Syk, Prkcb, Inpp5d, Nfkb1, Nfatc2, Lyn, Ppp3ca, Lilrb4a, Cd81, Nfatc1, Map2k2, Bcl10, Pirb, Lilra6, Gm14548, Hras, Ppp3r1, Ppp3cb, Akt2, Ikbkg, Ifitm1, Pik3r2, Gsk3b*
67	mmu04020	Calcium signaling pathway-Mus musculus (mouse)	41	0.015422981	3.18	*Ednrb, Adora2a, Mcoln2, Cd38, Phka2, Orai2, Cxcr4, Pdgfb, Tpcn2, Tpcn1, Hrh2, Nos2, Asph, Cacna1a, Mcoln3, Adora2b, Adcy3, Cacna1d, Ppp3cc, Pdgfc, Pde1b, Pdgfrb, Itpr3, Cysltr1, Plcb2, Adrb2, Calm1, P2rx4, Vdac3-ps1, Calm3, Vdac3, Pdgfa, Prkacb, Atp2a3, Cacna1b, P2rx5, Prkcb, Ppp3ca, Slc25a5, Pde1c, Plcg1, Stim2, Adcy7, Atp2b1, Vdac2, Zhx3, Adcy9, Htr2b, P2rx7, Camk2b, Htr2a, Gm7591, Gm5529, Hrc, Ppp3r1, Orai1, Ppp3cb, Plcb4, Gm16183, Phkb, Prkcg, Itpkb, Adra1a, Drd1, Slc8a1, Adcy2, Stim1, Plcd1, Gnas*
68	mmu05235	PD-L1 expression and PD-1 checkpoint pathway in cancer-Mus musculus (mouse)	69	0.017876821	5.36	*Tlr2, Nfkbie, Nfkbia, Cd28, Batf2, Rasgrp1, Cd274, Jak2, Batf, Stat1, Hif1a, Nfkbib, Fos, Jun, Mapk14, Ptpn6, Myd88, Rela, Cd247, Ppp3cc, Ifngr2, Ticam2, Mapk11, Map2k1, Map2k6, Nfkb1, Nfatc2, Ppp3ca, Nfatc1, Plcg1, Map2k2, Csnk2b, Zhx3, Map2k3, Tlr4, Hras, Ppp3r1, Ppp3cb, Akt2, Ikbkg, Stat3, Mapk12, Pik3r2*
69	mmu04630	JAK-STAT signaling pathway-Mus musculus (mouse)	43	0.018759207	3.34	*Socs3, Il7r, Cdkn1a, Il21r, Il15ra, Pim1, Jak2, Ccnd1, Il2rg, Stat2, Pdgfb, Stat1, Socs1, Irf9, Myc, Ptpn6, Csf3, Lifr, Csf2rb, Il6, Il12rb1, Aox1, Il12b, Ifngr2, Pdgfrb, Il12a, Il2rb, Il20rb, Il13ra1, Il15, Il23r, Il6st, Pdgfa, Pias4, Ptpn2, Il6ra, Osm, Il4ra, Bcl2l1, Il7, Cish, Pias1, Il4, Hras, Tyk2, Ccnd2, Ifnar2, Akt2, Lif, Pias3, Csf3r, Stat3, Il10, Pik3r2, Crlf2*
70	mmu05142	Chagas disease-Mus musculus (mouse)	55	0.021840297	4.27	*Tlr2, Tnf, Ccl5, Nfkbia, C3, Il1b, Fas, Ccl3, Cflar, Fos, Jun, Nos2, Calr, Mapk14, Ccl2, Myd88, Rela, Il6, Cd247, Il12b, Tlr6, Ifngr2, Il12a, Plcb2, Tgfbr2, Mapk11, Ppp2r2a, Tgfb3, Nfkb1, Ppp2cb, Ppp2ca, Serpine1, C1qa, Smad2, Tlr4, Tgfbr1, Mapk9, Akt2, Plcb4, Ikbkg, Ppp2r2d, Mapk12, Il10, Pik3r2, Gnas*
71	mmu05205	Proteoglycans in cancer-Mus musculus	45	0.022222264	3.49	*Sdc1, Tlr2, Tnf, Mmp9, Cdkn1a, Src, Itgb5, Hcls1, Vav3, Fas, Ccnd1, Cav1, Vegfa, Hif1a, Met, Ezr, Igf1, Myc, Itgb3, Ppp1r12b, Hpse, Mapk14, Fgfr1, Fzd1,*

		(mouse)				*Fzd7, Vav1, Plau, Ptpn6, Sdc4, Wnt6, Il12b, Pdcd4, Plaur, Tiam1, Mdm2, Itpr3, Hgf, Ppp1cb, Mmp2, Rras, Mapk11, Map2k1, Itga5, Prkacb, Itgav, Tfap4, Prkcb, Iqgap1, Eif4b, Actb, Plcg1, Map2k2, Igf1r, Fzd4, Gm38317, Zhx3, Itgb1, Smad2, Pak1, Fn1, Tlr4, Camk2b, Ppp1ca, Hras, Gpc1, Ptk2, Rhoa, Akt2, Wnt2b, Ptch1, Prkcg, Cav2, Rdx, Stat3, Kdr, Mapk12, Thbs1, Msn, Pik3r2, Trp53, Pxn*
72	mmu04979	Cholesterol metabolism-Mus musculus (mouse)	81	0.022222264	6.29	*Lpl, Ldlrap1, Nceh1, Cd36, Angptl4, Cyp27a1, Soat1, Scarb1, Pltp, Stard3, Sort1, Tspo, Lipa, Lrp1, Soat2, Vdac3-ps1, Vdac3, Lrpap1, Vdac2, Vapa, Gm7591, Npc1, Lipg*
73	mmu01521	EGFR tyrosine kinase inhibitor resistance-Mus musculus (mouse)	23	0.02327786	1.79	*Gas6, Src, Jak2, Pdgfb, Vegfa, Met, Igf1, Bcl2l11, Il6, Pdgfc, Shc4, Pdgfrb, Foxo3, Shc2, Hgf, Map2k1, Pdgfa, Eif4e, Bax, Prkcb, Il6ra, Plcg1, Map2k2, Igf1r, Bcl2l1, Zhx3, Bad, Axl, Gm12188, Hras, Akt2, Prkcg, Eif4ebp1, Stat3, Kdr, Eif4e2, Pik3r2, Gsk3b*
74	mmu04750	Inflammatory mediator regulation of TRP channels-Mus musculus (mouse)	38	0.027432009	2.95	*Src, Il1b, Igf1, Pla2g4a, Mapk14, Prkch, Adcy3, Itpr3, Ppp1cb, Plcb2, Mapk11, Calm1, Calm3, Prkacb, Ptger4, Prkcd, Map2k6, Trpv4, Prkcb, Plcg1, Adcy7, Zhx3, Map2k3, Adcy9, P2ry2, Htr2b, Camk2b, Ppp1ca, Htr2a, Mapk9, Il1rap, Plcb4, Prkcg, Trpv2, Adcy2, Mapk12, Alox12, Pik3r2, Gnas*
75	mmu00564	Glycerophospholipid metabolism-Mus musculus (mouse)	39	0.03003575	3.03	*Plpp1, Lpcat2, Gpd2, Pgs1, Pla2g15, Plpp3, Lpin1, Pla2g4a, Agpat1, Pla2g16, Dgkd, Dgka, Agpat4, Pisd-ps1, Cept1, AC149090.1, Gpat4, Dgkz, Gpd1l, Zfp652, Pnpla7, Pcyt1a, Dgkg, Abi3, Agpat3, Adprm, Plpp2, Dgkh, Cds2, Lpcat1, Etnk1, Lpgat1, Mboat1, Gpcpd1, Ptdss2, Pla1a, Lypla2, Agpat5, Plpp5, Pcyt2, Lpcat4, Gnpat*
76	mmu04015	Rap1 signaling pathway-Mus musculus (mouse)	42	0.031253442	3.26	*Vasp, Fpr1, Rasgrp3, Adora2a, Src, Lcp2, Vav3, Rac2, Pdgfb, Vegfa, Ralgds, P2ry1, Itgal, Met, Csf1, Igf1, Lpar1, Itgb3, Mapk14, Fgfr1, Vav1, Kitl, Adora2b, Adcy3, Tiam1, Pdgfc, Arap3, Pdgfrb, Hgf, Efna2, Plcb2, Sipa1l2, Rras, Rassf5, Rapgef2, Rapgef3, Pfn1, Mapk11, Map2k1, Calm1, Rapgef6, Calm3, Lpar2, Pdgfa, Lpar5, Rap1b, Map2k6, Prkcb, Apbb1ip, Vegfc, Actb, Plcg1, Map2k2, Igf1r, Adcy7, Prkd2, Pgf, Prkd3, Rala, Zhx3, Map2k3, Adcy9, Itgb1, Id1, Hras, Bcar1, Evl, Rapgef5, Rhoa, Akt2, Plcb4, Prkcg, Magi3, Afdn, Sipa1l1, Adcy2, Kdr,*

						Mapk12, Insr, Thbs1, Prkci, Pik3r2, Gnas, Angpt2
77	mmu05134	Legionellosis-Mus musculus (mouse)	84	0.031817565	6.52	*Tlr2, Tnf, Cxcl2, Nfkbia, C3, Cxcl3, Il1b, Nfkb2, Cxcl1, Cd14, Vcp, Gm6548, Myd88, Rela, Il6, Il12b, Cycs, Gm10053, Il12a, Vcp-rs, Hspa8, Casp9, Naip5, Casp7, Casp1, Arf2, Nfkb1, Hspa1b, Hspd1, Sar1a, Sec22b, Arf1, Hspa2, Tlr4, Rab1a, Naip1, Naip2, Sar1b, Gm8355, Clk1, Hbs1l, Gm4366, Naip3*
78	mmu04723	Retrograde endocannabinoid signaling-Mus musculus (mouse)	43	0.033226328	3.34	*Ptgs2, Gng12, Daglb, Mapk14, Cacna1a, Gng2, Gngt2, Adcy3, Cacna1d, Itpr3, Plcb2, Dagla, Mapk11, Gnb1, Prkacb, Cacna1b, Prkcb, Adcy7, Mgll, Ndufb6, Ndufa11, Adcy9, Gnb2, Ndufb9, Ndufa5, Ndufa3, mt-Nd2, Ndufs6, Ndufc1, Ndufa2, Kcnj6, mt-Nd4l, Mapk9, Plcb4, Ndufs4, Gm16183, Ndufb8, Ndufa9, Prkcg, Kcnj9, Ndufab1, Ndufa13, Ndufa10, Ndufab1-ps, mt-Nd5, Ndufv2, Ndufv1, Adcy2, Ndufs3, Ndufb3, Mapk12, Ndufa1, Ndufb7, Gng7, Ndufs7, Ndufa12, Ndufa8, Ndufs8*
79	mmu00062	Fatty acid elongation-Mus musculus (mouse)	58	0.034420584	4.50	*Acot1, Acot7, Hadhb, Mecr, Hacd4, Gm13910, Ppt2, Hsd17b12, Elovl5, Acot2, Elovl6, Elovl1, Hadha, Hadh, Acaa2, Ppt1, Hacd3*
80	mmu04917	Prolactin signaling pathway-Mus musculus (mouse)	17	0.034420584	1.32	*Socs3, Irf1, Src, Jak2, Ccnd1, Stat1, Socs1, Fos, Mapk14, Rela, Shc4, Foxo3, Shc2, Mapk11, Map2k1, Tnfrsf11a, Nfkb1, Map2k2, Cish, Gm17334, Hras, Ccnd2, Mapk9, Akt2, Stat3, Mapk12, Pik3r2, Gsk3b*
81	mmu04623	Cytosolic DNA-sensing pathway-Mus musculus (mouse)	28	0.048004289	2.17	*Irf7, Zbp1, Ccl5, Nfkbia, Cxcl10, Il1b, Ddx58, Ikbke, Nfkbib, Ccl4, Rela, Il6, Adar, Polr3d, Mb21d1, Tbk1, Polr3g, Casp1, Nfkb1, Polr2l, Polr3c, Ikbkg, Polr2k, Tmem173, Polr3gl, Polr2f*
