# Supplementary material for: Transcriptomic Changes in Mouse Bone Marrow-Derived Macrophages Exposed to Neuropeptide FF
Source: Genes (Basel). 2021 May 9;12(5):705. doi: 10.3390/genes12050705 (PMC8151073; doi:10.3390/genes12050705)
Supplement: Supplementary file 1 [file genes-12-00705-s001.zip › genes-1147651-supplementary/Table S7 GO analysis of hub genes.pdf]

Table S7. GO analysis of hub genes

**Gene**	**GO analysis**[1, 2]
Cnr2	MF: signaling receptor activity<br>BP: immune system process; response to stimulus; signaling<br>CC: cell projection; endoplasmic reticulum; plasma membrane
Gpr55	MF: signaling receptor activity<br>BP: cell differentiation; immune system process; response to stimulus; signaling; system development<br>CC: plasma membrane
Gpr18	MF: signaling receptor activity<br>BP: cell differentiation; homeostatic process; immune system process; response to stimulus; signaling; system development<br>CC: cytoplasmic vesicle; plasma membrane
Hcar2	MF: carbohydrate derivative binding; signaling receptor activity<br>BP: cell death; establishment of localization; homeostatic process; immune system process; lipid metabolic process; response to stimulus; signaling<br>CC: plasma membrane
Gpr31b	MF: lipid binding; signaling receptor activity<br>BP: İmmune system process; response to stimulus; signaling<br>CC: plasma membrane
	MF: lipid binding; signaling receptor activity

Gpr183	BP: cell differentiation; cell population proliferation; homeostatic process; immune system process; response to stimulus; signaling; system development<br><br>CC: plasma membrane
Oas2	MF: carbohydrate derivative binding; RNA binding; transferase<br><br>BP: establishment of localization; immune system process; response to stimulus; signaling; system development<br><br>CC: cytosol; nucleus; organelle lumen
Dhx58	MF: carbohydrate derivative binding; DNA binding; hydrolase; RNA binding<br><br>BP: immune system process; response to stimulus<br><br>CC: none

Note: Cellular Component; MF: Molecular Function; CC: BP: Biological Process. This information was acquired from the Mouse Genome Database (MGD) at the Mouse Genome Informatics website, the Jackson Laboratory, Bar Harbor, Maine (URL: http://www.informatics.jax.org) [40, 41] [date of retrieving data: December 28, 2020]

[1] Smith CM, Hayamizu TF, Finger JH, Bello SM, McCright IJ, Xu J, Baldarelli RM, Beal JS, Campbell J, Corbani LE, Frost PJ, Lewis JR, Giannatto SC, Miers D, Shaw DR, Kadin JA, Richardson JE, Smith CL, Ringwald M. Nucleic Acids Res 2019;47(D1):D774-d779.

[2] Bult CJ, Blake JA, Smith CL, Kadin JA, Richardson JE. Nucleic Acids Res 2019;47(D1):D801-d806.
