# Supplementary material for: Transcriptomic Changes in Mouse Bone Marrow-Derived Macrophages Exposed to Neuropeptide FF
Source: Genes (Basel). 2021 May 9;12(5):705. doi: 10.3390/genes12050705 (PMC8151073; doi:10.3390/genes12050705)
Supplement: Supplementary file 1 [file genes-12-00705-s001.zip › genes-1147651-supplementary/Table S8 transcriptor factors-up DEGs.pdf]

Table S8. Transcriptional factors tied to the up-regulated DEGs

#	Key TF	Description	# of overlapped genes	P value	FDR
1	*Nfkb1*	nuclear factor of kappa light polypeptide gene enhancer in B cells 1, p105	13	1.06e-16	2.55e-15
2	*Stat1*	signal transducer and activator of transcription 1	5	7.79e-09	6.97e-08
3	*Jun*	jun proto-oncogene	7	8.71e-09	6.97e-08
4	*Rela*	v-rel reticuloendotheliosis viral oncogene homolog A (avian)	6	1.72e-07	9.23e-07
5	*Irf1*	interferon regulatory factor 1	4	1.92e-07	9.23e-07
6	*Cebpb*	CCAAT/enhancer binding protein (C/EBP), beta	4	1.66e-06	6.09e-06
7	*Ikbkb*	inhibitor of kappaB kinase beta	3	1.78e-06	6.09e-06
8	*Fos*	FBJ osteosarcoma oncogene	4	5.33e-06	1.6e-05
9	*Irf8*	interferon regulatory factor 8	3	1.41e-05	3.75e-05

10	*Hdac1*	histone deacetylase 1	3	2.59e-05	5.64e-05
11	*Rel*	reticuloendotheliosis oncogene	3	2.59e-05	5.64e-05
12	*Ep300*	E1A binding protein p300	3	0.000258	0.000516
13	*Egr1*	early growth response 1	3	0.000424	0.000783
14	*Pou2f2*	POU domain, class 2, transcription factor 2	2	0.000555	0.000928
15	*Stat3*	signal transducer and activator of transcription 3	3	0.00058	0.000928
16	*Klf4*	Kruppel-like factor 4 (gut)	2	0.000696	0.00104
17	*Ahr*	aryl-hydrocarbon receptor	2	0.000936	0.00132
18	*Cebpa*	CCAAT/enhancer binding protein (C/EBP), alpha	2	0.00112	0.00149
19	*Spi1*	spleen focus forming virus (SFFV) proviral integration oncogene	2	0.00309	0.00389
20	*Crebbp*	CREB binding protein	2	0.00324	0.00389
21	*Ppara*	peroxisome proliferator activated receptor alpha	2	0.00407	0.00465

22	*Sp3*	trans-acting transcription factor 3	2	0.0127	0.0139
23	*Trp53*	transformation related protein 53	2	0.0466	0.0486
24	*Sp1*	trans-acting transcription factor 1	2	0.107	0.107

Note: data acquired from TRRUST (version 2) (https://www.grnpedia.org/trrust/)
