# Supplementary material for: Transcriptomic Changes in Mouse Bone Marrow-Derived Macrophages Exposed to Neuropeptide FF
Source: Genes (Basel). 2021 May 9;12(5):705. doi: 10.3390/genes12050705 (PMC8151073; doi:10.3390/genes12050705)
Supplement: Supplementary file 1 [file genes-12-00705-s001.zip › genes-1147651-supplementary/Table S9 transcriptor factors-down DEGs.pdf]

Table S9. Transcriptional factors tied to the down-regulated DEGs

#	Key TF	Description	# of overlapped genes	P value	FDR
1	*Sp3*	trans-acting transcription factor 3	7	0.000239	0.00812
2	*Esr1*	estrogen receptor 1 (alpha)	4	0.000854	0.012
3	*Atf2*	activating transcription factor 2	3	0.00159	0.012
4	*Gtf2i*	general transcription factor II I	3	0.00159	0.012
5	*Esr2*	estrogen receptor 2 (beta)	3	0.00191	0.012
6	*Sp1*	trans-acting transcription factor 1	11	0.0025	0.012
7	*Nr0b2*	nuclear receptor subfamily 0, group B, member 2	3	0.00266	0.012
8	*Smad4*	SMAD family member 4	4	0.00289	0.012
9	*Ehmt2*	euchromatic histone lysine N-methyltransferase 2	2	0.00319	0.012
10	*Egr1*	early growth response 1	5	0.00414	0.0141

11	*Sp4*	trans-acting transcription factor 4	2	0.00584	0.018
12	*Ncoa3*	nuclear receptor coactivator 3	2	0.00743	0.021
13	*Gfi1*	growth factor independent 1	2	0.0092	0.024
14	*Ppard*	peroxisome proliferator activator receptor delta	2	0.0111	0.027
15	*Ep300*	E1A binding protein p300	4	0.0122	0.0277
16	*Xbp1*	X-box binding protein 1	2	0.0155	0.0329
17	*Cebpb*	CCAAT/enhancer binding protein (C/EBP), beta	3	0.0245	0.049
18	*Stat3*	signal transducer and activator of transcription 3	4	0.0303	0.0573
19	*Foxo1*	forkhead box O1	3	0.0364	0.0619
20	*Smad3*	SMAD family member 3	3	0.0364	0.0619
21	*Pparg*	peroxisome proliferator activated receptor gamma	3	0.0384	0.0621
22	*Pitx2*	paired-like homeodomain transcription factor 2	2	0.0492	0.0761

23	*Creb1*	cAMP responsive element binding protein 1	2	0.0775	0.115
24	*Rela*	v-rel reticuloendotheliosis viral oncogene homolog A (avian)	4	0.107	0.152
25	*Spi1*	spleen focus forming virus (SFFV) proviral integration oncogene	2	0.119	0.162
26	*Crebbp*	CREB binding protein	2	0.124	0.163
27	*Ppara*	peroxisome proliferator activated receptor alpha	2	0.15	0.189
28	*E2f1*	E2F transcription factor 1	2	0.165	0.194
29	*Myc*	myelocytomatosis oncogene	2	0.165	0.194
30	*Nfkb1*	nuclear factor of kappa light polypeptide gene enhancer in B cells 1, p105	5	0.179	0.203
31	*Nfe2l2*	nuclear factor, erythroid derived 2, like 2	2	0.187	0.205
32	*Ets1*	E26 avian leukemia oncogene 1, 5' domain	2	0.219	0.233
33	*Ctnnb1*	catenin (cadherin associated protein), beta 1	2	0.236	0.243

34	*Trp53*	transformation related protein 53	2	0.714	0.714

Note: data obtained from TRRUST (version 2) (https://www.grnpedia.org/trrust/)
